# Resistance of Transmitted Founder HIV-1 to IFITM-Mediated Restriction

**DOI:** 10.1016/j.chom.2016.08.006

**Published:** 2016-10-12

**Authors:** Toshana L. Foster, Harry Wilson, Shilpa S. Iyer, Karen Coss, Katie Doores, Sarah Smith, Paul Kellam, Andrés Finzi, Persephone Borrow, Beatrice H. Hahn, Stuart J.D. Neil

**Affiliations:** 1 Department of Infectious Diseases, King’s College London Faculty of Life Sciences and Medicine, Guy’s Hospital, London SE1 9RT, UK; 2 Departments of Medicine and Microbiology, Perelman School of Medicine, University of Pennsylvania, Philadelphia, PA 19104, USA; 3 Wellcome Trust Sanger Centre, Hinxton, Cambridge CB10 1SA, UK; 4 Centre de Recherche du CHUM and Department of Microbiology, Infection, and Immunology, Université de Montréal, Montréal, QC H3T 1J4, Canada; 5 Nuffield Department of Medicine, University of Oxford, Oxford OX1 2JD, UK

**Keywords:** HIV-1, IFITM, type 1 interferon, antiviral restriction, CD4, co-receptor, transmitted founder virus, neutralization, escape

## Abstract

Interferon-induced transmembrane proteins (IFITMs) restrict the entry of diverse enveloped viruses through incompletely understood mechanisms. While IFITMs are reported to inhibit HIV-1, their in vivo relevance is unclear. We show that IFITM sensitivity of HIV-1 strains is determined by the co-receptor usage of the viral envelope glycoproteins as well as IFITM subcellular localization within the target cell. Importantly, we find that transmitted founder HIV-1, which establishes de novo infections, is uniquely resistant to the antiviral activity of IFITMs. However, viral sensitivity to IFITMs, particularly IFITM2 and IFITM3, increases over the first 6 months of infection, primarily as a result of neutralizing antibody escape mutations. Additionally, the ability to evade IFITM restriction contributes to the different interferon sensitivities of transmitted founder and chronic viruses. Together, these data indicate that IFITMs constitute an important barrier to HIV-1 transmission and that escape from adaptive immune responses exposes the virus to antiviral restriction.

## Highlights

• IFITM restriction of HIV-1 depends on virus co-receptor use and IFITM localization
• Transmitted founder viruses are resistant to IFITM restriction
• Escape from early neutralizing antibodies confers IFITM sensitivity
• IFITM restriction contributes to the increased IFN sensitivity of chronic viruses

## Graphical Abstract

**Figure:**
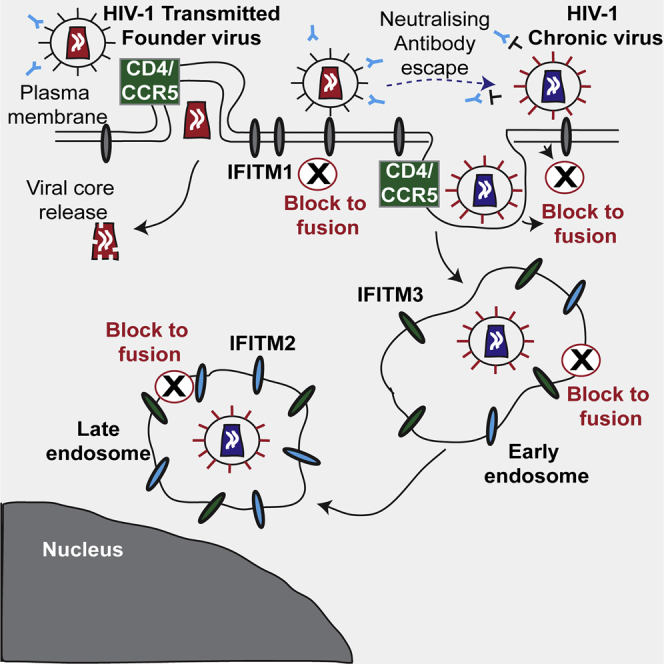

## Teaser

IFITMs are interferon-induced proteins with broad antiviral activity. Foster et al. demonstrate that HIV-1 sensitivity to IFITMs depends on the viral entry route into target cells. Strikingly, transmitted HIV-1 strains are IFITM resistant, but viral envelope mutations that escape neutralizing antibody responses after infection lead to IFITM and interferon sensitivity.

## Introduction

Robust systemic type 1 interferon (IFN-1) responses are among the earliest host innate immune defenses during acute SIV and HIV-1 infection (Abel et al., 2005, Stacey et al., 2009). In primary CD4^+^ T cells and macrophages, expression of IFN-induced genes (ISGs) restricts viral replication (Goujon and Malim, 2010), and treatment of rhesus macaques with IFN-1 increased the number of intrarectal challenges required to achieve systemic SIVmac infection and reduced the number of transmitted founder (TF) viruses (Sandler et al., 2014). While the virus encodes countermeasures against important ISGs, such as APOBEC3G and tetherin (BST2/CD317), other ISGs appear to restrict viral replication in cell culture with no obvious viral evasion mechanism, and thus their physiological relevance in the transmission and pathogenesis of HIV/AIDS remains unclear (Doyle et al., 2015).

One such family of ISGs, the IFN-induced transmembrane proteins 1–3 (IFITMs 1–3), has broad activity against diverse enveloped viruses, particularly influenza A virus (IAV) (reviewed in Smith et al., 2014). IFITMs are small (125–135 amino acids) membrane-spanning proteins whose topology is still a matter of debate. In the most favored conformation (Bailey et al., 2013, Ling et al., 2016, Weston et al., 2014), the N-terminal region is cytosolic, followed by a semi-transmembrane (TM) domain that remerges from the cytosolic face, which, after an intracellular loop containing essential palmitoylation sites (Yount et al., 2010), turns into a canonical TM helix that exposes the C terminus on the extracellular side. In humans IFITMs 2 and 3 are highly homologous with only ten amino acid differences between them. Both have longer N-terminal tails than IFITM1, in which an overlapping PPXY and YxxΦ site interacts with NEDD4 family ubiquitin ligases (Chesarino et al., 2015) and the clathrin adaptor AP-2, respectively (Jia et al., 2014). IFITM2 and 3 localize predominantly to different endosomal compartments at steady state (Weston et al., 2014). This is determined in part by the AP-2 binding, implying that they traffic via the cell surface (Jia et al., 2012, Jia et al., 2014, Weston et al., 2014). By contrast, IFITM1 lacks an obvious trafficking sequence and is primarily expressed at the plasma membrane. A human polymorphism defined by a SNP, rs12252-C, has been proposed to lead to an alternatively spliced variant of IFITM3 that truncates the N terminus after the YxxΦ motif, thereby reducing its antiviral activity against IAV and accounting for enhanced morbidity in the recent H1N1 swine flu pandemic (Everitt et al., 2012).

IFITM3 has been shown to restrict IAV entry at the stage of fusion in the endosome (Amini-Bavil-Olyaee et al., 2013, Desai et al., 2014, Li et al., 2013). Although the mechanism is not well understood, this restriction may be due to the effects of IFITM3 on membrane fluidity and/or cholesterol trafficking or biosynthesis (Amini-Bavil-Olyaee et al., 2013, Desai et al., 2014, Lin et al., 2013). In contrast to the effects of IFITMs on pH-dependent virus entry, their ability to restrict HIV-1 is less clear cut. The antiviral effects of IFITMs so far observed have been variously ascribed to virion incorporation during assembly (Compton et al., 2014, Tartour et al., 2014) or inhibition of processing of the HIV-1 envelope glycoprotein (Yu et al., 2015), with little consensus as to their mechanism and sites of action. It is, however, difficult to rationalize these mechanisms with the clear inhibitory effects in the target cell for all other enveloped viruses thus far examined. Moreover, the effect that IFITMs might have on HIV-1 tropism has not been studied.

HIV-1 enters cells through engagement of its envelope glycoprotein with CD4 and a chemokine receptor co-receptor, CCR5 (R5) or CXCR4 (X4) (Wilen et al., 2012). CD4 interaction induces conformational changes in the surface (SU) subunit of Env, gp120, which exposes the co-receptor-binding site. Upon co-receptor engagement, further conformational changes in the TM gp41 subunit activate its membrane fusion capacity. R5 usage is essential for viral transmission, with a switch to X4 observed in ∼50% of patients with subtype B infections but less common in other clades. Furthermore, while R5 and X4 viruses are both capable of infecting CD4^+^ T cells, tropism of R5 strains for macrophages is more complex. Most R5 viruses, including TF viruses, cannot efficiently infect macrophages. Macrophage tropism is almost exclusively R5 dependent, but also requires adaptation in gp120 to use very low levels of CD4 (Duenas-Decamp et al., 2010). It is also not clear why, despite constitutive expression of CXCR4 on macrophages, most X4 viruses cannot infect them (Simmons et al., 1998). Finally, there are examples of HIV-1 and SIV restriction in certain cell types that appear dependent on the route of viral entry (Pineda et al., 2007, Schmitz et al., 2004). Although generally thought of as a virus that fuses at the cell surface, some HIV-1 strains fuse in endosomes in some cell types (Miyauchi et al., 2009).

Given these observations, we set out to examine whether IFITMs could restrict HIV-1 strains with differing receptor tropisms. In doing so, we found that IFITM restriction of HIV-1 is modulated by co-receptor usage and subcellular localization of the IFITM, suggesting different entry pathways depending on Env/receptor interactions. Furthermore, we found that TF viruses are uniquely IFITM resistant, a property that is lost during chronic infection, in part due to escape mutations acquired in response to autologous neutralizing responses.

## Results

### IFITMs Differentially Restrict HIV-1 Isolates Depending on Co-receptor Tropism

**Figure 1:**
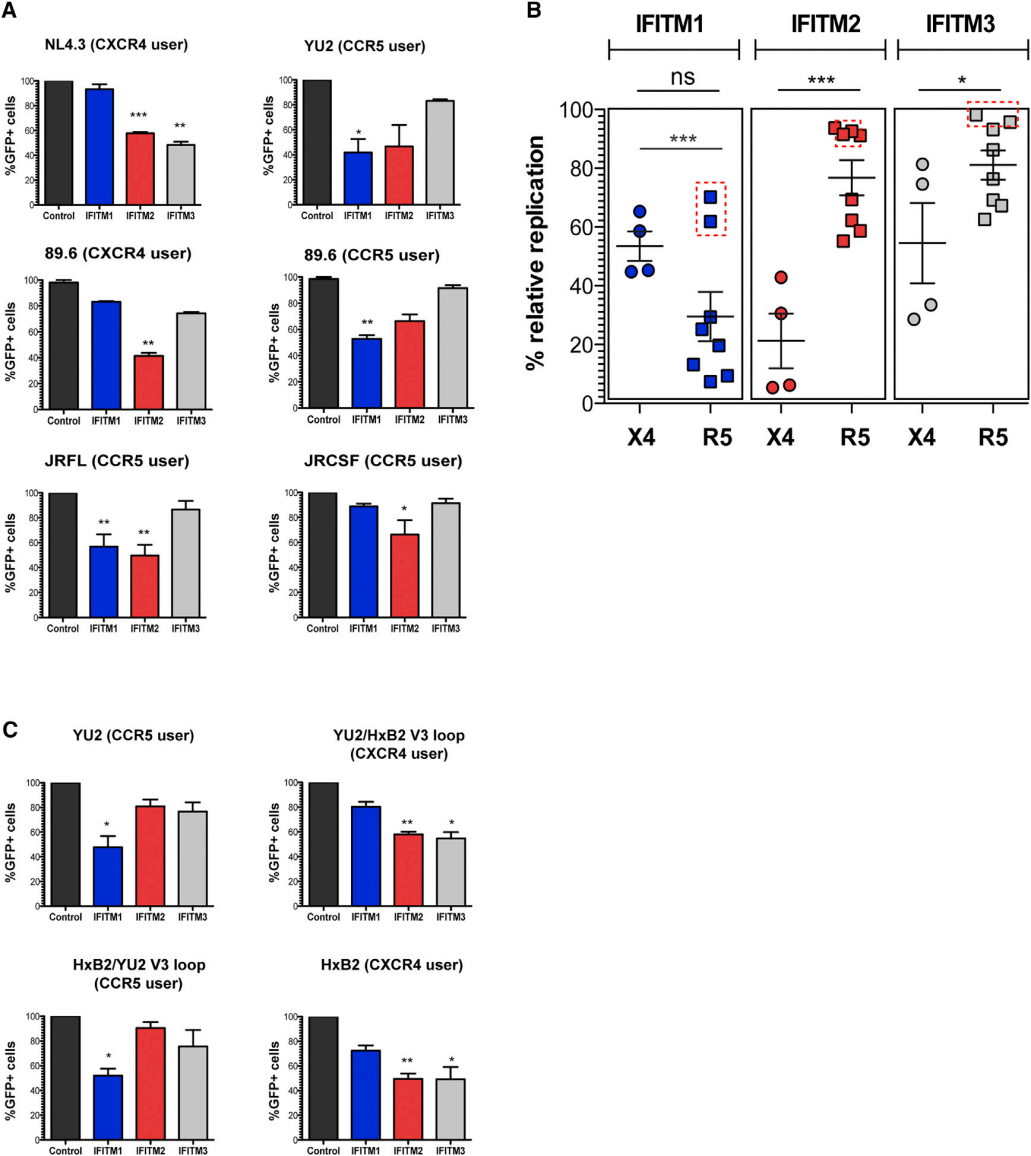
Sensitivity of HIV-1 to IFITM Inhibition Varies with Co-receptor Usage (A) GFP-encoding HIV-1 vectors pseudotyped with the indicated envelope glycoproteins were used to infect U87/CD4/CCR5 or U87/CD4/CXCR4 cells stably expressing IFITMs 1, 2, or 3 or empty vector (control). The percentage of GFP^+^ cells compared to control was determined by flow cytometry, and results shown represent a mean of independent experiments. Statistics were performed using an unpaired two-tailed t test (^∗∗∗^p < 0.001; ^∗∗^p < 0.01; ^∗^p < 0.05; ns, p > 0.05). (B) Cumulative viral replication at 96 hr in the presence of IFITMs differs for CXCR4-using or CCR5-using HIV-1 virus isolates. U87/CD4/CCR5 or U87/CD4/CXCR4 cells were infected at an MOI of 0.05, and replication was monitored by viral output on HeLa-TZMbl indicator cells (see also Figures S1D and S1E). Each virus point represents a mean of three independent experiments. Inset red dashed boxes: CCR5-using CH105 and RHPA are founder virus isolates that are resistant to IFITM inhibition. Removal of these outliers reveals data significance between CXCR4 and CCR5 users in the presence of IFITM1 (^∗∗∗^p < 0.001; ^∗∗^p < 0.01; ^∗^p < 0.05; ns, p > 0.05, based on an unpaired two-tailed t test). (C) Pseudotyped HIV vectors were produced with wild-type YU2 and Hxb2 envelopes or V3 loop swap variants and analyzed as in (A). All error bars represent ± SEM (n = 3). See also Figure S1.

To examine the role of IFITMs in restricting HIV-1 replication, we constructed U87 neuroblastoma cells (which do not express detectable IFITMs without IFN induction) encoding CD4 together with one of the two major co-receptors, CXCR4 or CCR5, to express IFITM1, IFITM2, and IFITM3, respectively (Figure S1A, available online). Importantly, the IFITM expression levels in these engineered cell lines were of a similar magnitude compared to those of monocyte-derived macrophages and CD4^+^ T cells in the presence and absence of IFN-1 (Figure S1B). IFITM expression did not alter CD4 or co-receptor expression in these cells (Figure S1C). We then analyzed the infectivity of HIV-1 Env pseudotypes as well as replication-competent molecular clones in these cells (Figures 1A, 1B, S1D, and S1E). Testing both single-round infectivity and cumulative 96-hr replication, we found that X4-using viruses displayed a significantly greater sensitivity to IFITMs 2 and 3 than did the R5 viruses (Figures 1, S1D, and S1E). In contrast, most R5 viruses tested were more sensitive to IFITM1 than X4 viruses. However, this difference achieved statistical significance only after the removal of two outlier R5 strains (CH105 and THRO), the reasons for which will be addressed below. Intriguingly the R5/X4 isolate 89.6 displayed distinct restriction patterns dependent on whether it entered target cells using CXCR4 or CCR5 (Figures 1A, 1B, and S1D). In the former case it had a singular sensitivity to IFITM2, whereas in the latter case this was relieved in favor of greater IFITM1 sensitivity.

To formally demonstrate that co-receptor usage influences IFITM sensitivity, we pseudotyped lentiviral vectors with envelopes from prototypic R5 (YU2) and X4 (HxB2) using strains in which the determinant of co-receptor use, the V3 loop (Sullivan et al., 1998), had been exchanged. This resulted in exchange of the restriction phenotype between the two envelope proteins (Figure 1C). This indicates that a given IFITM’s antiviral activity is modulated by the receptor requirements of the infecting virus. Importantly one-round infectivities and multiple-round replication results correlated, and we saw no differential effects of IFITM expression on envelope precursor processing to gp120 (Figure S1F), suggesting IFITM restriction only affected Env-mediated cell entry, in contrast to a previous report (Yu et al., 2015).

### Subcellular Localization of IFITMs Correlates with Differential HIV-1 Restriction

**Figure 2:**
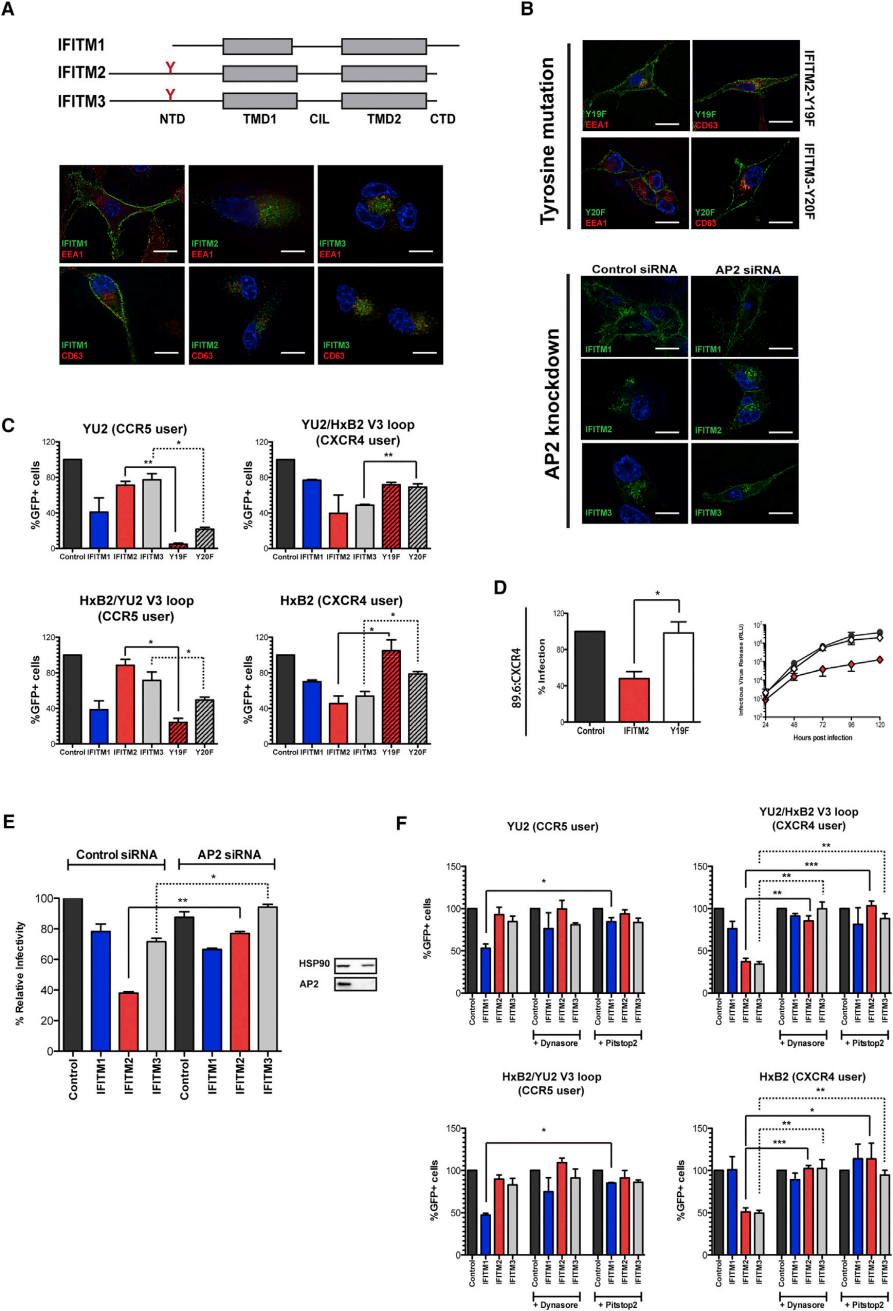
A Conserved N-Terminal Tyrosine Residue Is Crucial for IFITM Localization and Affects the IFITM Inhibition Phenotypes of HIV-1 (A) Schematic representation of human IFITM1, IFITM2, and IFITM3 proteins, indicating the localization of a conserved tyrosine at the N terminus of IFITMs 2 (Y19) and 3 (Y20). The localization of the IFITM proteins was assessed using U87 cells transduced with the indicated HA-tagged IFITM. Cells were stained with anti-HA (green) antibody and co-stained with early or late endosomal markers anti-EEA1 (red) or anti-CD63 (red). (B) Top: localization of HA-tagged IFITM2-Y19F or IFITM3-Y20F in U87 cells as in (A). Bottom: localization of HA-tagged IFITM2 or IFITM3 in U87, with or without small interfering RNA (siRNA)-mediated knockdown of AP-2, is shown. (C) As in Figure 1C, pseudotyped viruses were produced with wild-type YU2 and Hxb2 envelopes as well as V3 loop swaps. The percentage of infected cells, in the presence of either wild-type or mutant IFITM protein, was determined by flow cytometry (^∗∗∗^p < 0.001; ^∗∗^p < 0.01; ^∗^p < 0.05; ns, p > 0.05, unpaired two-tailed t test). (D) U87/CD4/CXCR4 cells expressing IFITM2 or IFITM2-Y19F were infected with 89.6 env-pseudotyped HIV-1 vector (left) or with full-length HIV-1 89.6 at an MOI of 0.05 (right), and they were analyzed as in Figures 1A and S1D, respectively (^∗∗∗^p < 0.001, unpaired two-tailed t test). (E) The effect of AP2 siRNA knockdown in U87/CD4/CXCR4 on the inhibition of 89.6 proviral replication. Cells were treated with control or AP2-specific SMARTpool siRNA and infected with 89.6. Then 48 hr post-infection, supernatants were assessed for viral production, and lysates were examined for the expression of AP2 and loading control HSP90 by western blot. Statistical significance was determined by using an unpaired two-tailed t test (^∗∗∗^p < 0.001; ^∗∗^p < 0.01; ^∗^p < 0.05; ns, p > 0.05). (F) Effect of endocytosis inhibitors dynasore and Pitstop2 on IFITM restriction of pseudotyped virus entry. U87/CD4/CoR cells expressing the indicated IFITM were exposed to dynasore (80 μM), Pitstop 2 (30 μM), or DMSO as a control, for 30 min prior to infection with the indicated env-pseudotyped viruses. The percentage of GFP^+^ cells was determined by flow cytometry (^∗∗∗^p < 0.001; ^∗∗^p < 0.01; ^∗^p < 0.05; ns, p > 0.05, unpaired two-tailed test). All error bars represent ± SEM (n = 3). See also Figure S2.

The differential restriction of X4 and R5 tropic HIV-1 prompted us to examine whether IFITM localization underlies these phenotypes. Available antibodies cannot distinguish between human IFITMs by immunofluorescence, so we used C-terminally HA-tagged proteins stably expressed in U87 cells. Unlike IFITM1, which is predominantly found at the cell surface, IFITMs 2 and 3 localize to endosomal compartments (Figure 2A) (Weston et al., 2014). Endosomal localization of IFITM2 and 3 depends on a YXXΦ-binding site for the clathrin adaptor AP-2. When the Y residue is mutated to a phenylalanine, both proteins readily localize to the cell surface (Figure 2B). Using the V3 loop-swap viral pseudotypes, we found that we could reverse the restriction patterns observed above: R5 tropism was sensitive to surface-expressed IFITM2 and 3, whereas X4 tropic restriction was relieved (Figure 2C). Similarly, the restriction of replication of the dual tropic 89.6 virus by IFITM2 in a CXCR4 context was completely abolished upon its relocalization to the cell surface (Figure 2D). We could mirror these findings by reversing 89.6’s IFITM2 sensitivity by knocking down AP-2 in CXCR4-expressing cells (Figure 2E). We also could show that the V3 swap variants that use X4 could be rescued from IFITM2 and 3 restriction by the inhibition of endocytosis with the dynamin inhibitor dynasore and the clathrin inhibitor Pitstop2 (Figure 2F). The differential restriction patterns depending on IFITM localization suggest that X4 HIV-1 isolates may fuse preferentially in different subcellular compartments compared to R5 viruses.

Recent publications suggest that IFITM incorporation into HIV-1 particles affects their infectivity and contributes to viral restriction (Compton et al., 2014, Tartour et al., 2014). We examined incorporation of IFITMs and mutants into CD45-depleted pelleted 89.6 virions (Coren et al., 2008). Despite the differential restriction of viral replication by endosomal IFITMs and mutants, we found no evidence that this correlated with virion incorporation, which remained constant (Figure S2B). Thus, restriction of HIV-1 entry depends on the localization of the IFITM in the target cell, and it suggests that viral sensitivity is the result of co-receptor-mediated targeting of the entry process to subcellular compartments where IFITMs are differentially expressed.

### TF HIV-1 Strains Are Resistant to IFITMs

**Figure 3:**
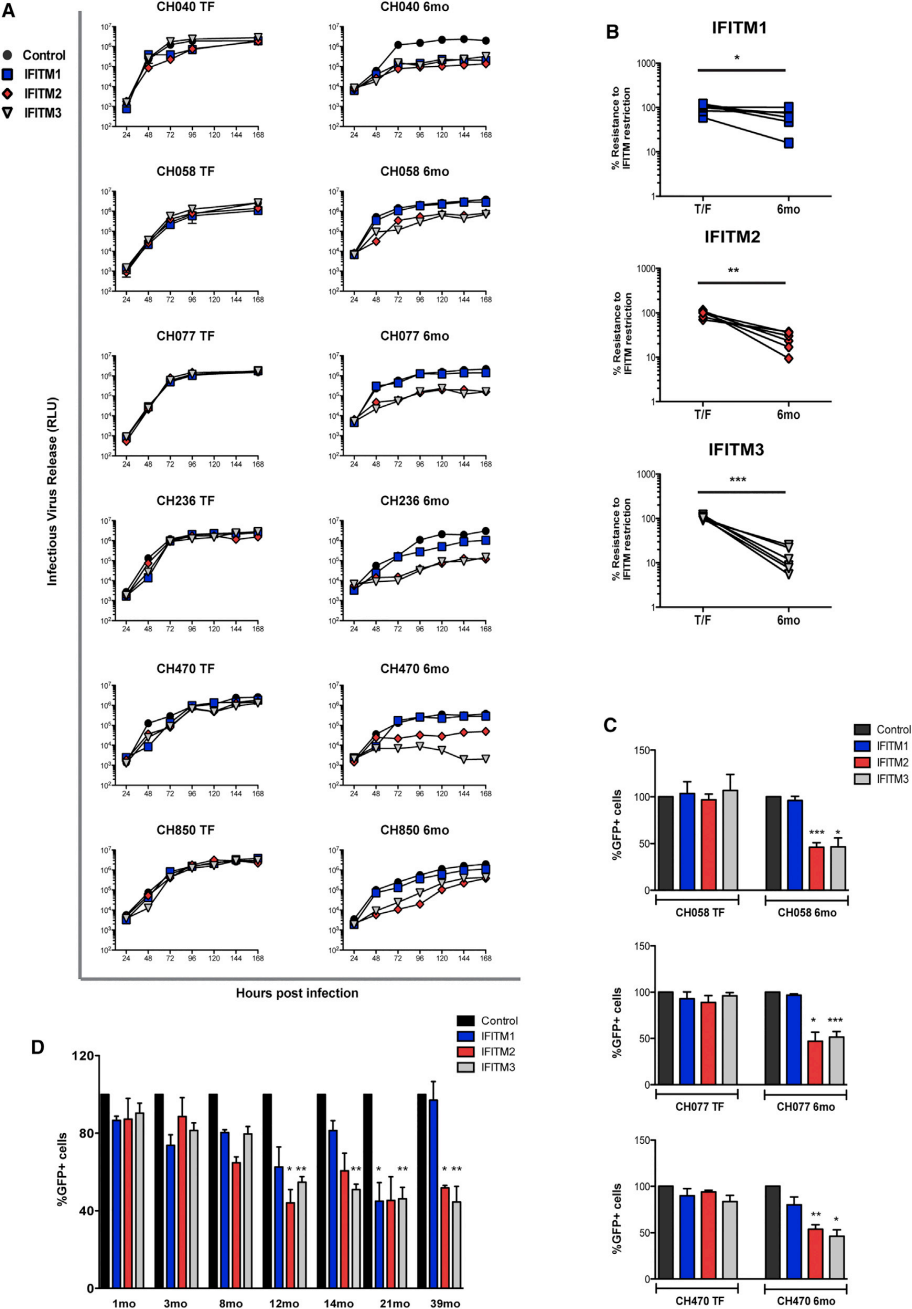
TF Virus Replication Is Resistant to IFITMs, with Sensitivity Arising at 6 Months (A) U87/CD4/CCR5 IFITM-expressing cells were infected with the indicated TF molecular clones or cognate 6-month variant at an MOI of 0.05, and infection was assayed with supernatants harvested every 24 hr for 7 days. Viral production was measured on HeLa-TZMbl indicator cells. Data represent a summary of three independent experiments. (B) Comparison of the relative cumulative replication in 3A at the 96-hr time point was analyzed by a paired Mann-Whitney test (^∗∗∗^p < 0.001; ^∗∗^p < 0.01; ^∗^p < 0.05; ns, p > 0.05). (C) HIV-1 vectors pseudotyped with envelopes of CH058, CH077, and CH470 TF and 6-month viruses were used to infect U87/CD4/CCR5 IFITM cells, analyzed as in Figure 1A. The percentage of infected cells was determined by flow cytometry, and results shown represent three independent infection experiments (^∗∗∗^p < 0.001; ^∗∗^p < 0.01; ^∗^p < 0.05; ns, p > 0.05, unpaired two-tailed test). (D) Sequential envelopes derived from the CAP256 patient (CAP256.1MO.C7J [1 month], CAP256.3MO.C9 [3 months], CAP256.8MO.31 [8 months], CAP256.12MO.1 [12 months], CAP256.14MO.5b [14 months], CAP256.21MO.A1 [21 months], and CAP256.39MO.10 [39 months]) were used to produce pseudotyped HIV-1 vectors and infect of U87/CD4/CCR5 IFITM cells (^∗∗^p < 0.01; ^∗^p < 0.05; ns, p > 0.05, unpaired two-tailed test). All error bars represent ± SEM (n = 3). See also Figure S3 and Table S1.

In the analysis shown in Figure 1, several viruses were almost completely resistant to IFITM restriction. In all cases these were TF viruses, which represent viruses that establish de novo infection following HIV-1 transmission (Salazar-Gonzalez et al., 2009). Using a panel of TF molecular clones (Figure 3A) as well as individual TF Envs (Figure S3A), we found that in U87CD4/CCR5 cells the great majority of these viruses were resistant to all three human IFITMs, with relocalization to the plasma membrane of IFITMs 2 and 3 having only minor effects (Figure S3B). For six TF viruses, matched 6-month consensus molecular clones from the same individual were available, all of which previously had been shown to be particularly sensitive to the antiviral effects of IFN-1 (Fenton-May et al., 2013). In all of these cases, we observed a striking increase in their sensitivities to IFITM2 and IFITM3 (Figures 3A and 3B), which was relieved upon IFITM2/3 relocalization to the plasma membrane (Figure S3B). All the TF/6-month pairs were singularly R5 tropic except for CH077, where the founder was able to use CXCR4 at a lower efficiency (not shown). The IFITM phenotypes could be transferred to lentiviral vectors pseudotyped with the respective envelope glycoproteins, indicating that amino acid changes in Env during the transition from acute to chronic infection were associated with IFITM-mediated restriction (Figure 3C). Furthermore, a panel of Envs derived from a clade C-infected patient, generated at the time of transmission through 39 months post-infection (Doria-Rose et al., 2014), exhibited progressive sensitization to IFITMs 2 and 3, with clear phenotypes appearing by 8 months (Figure 3D).

**Figure 4:**
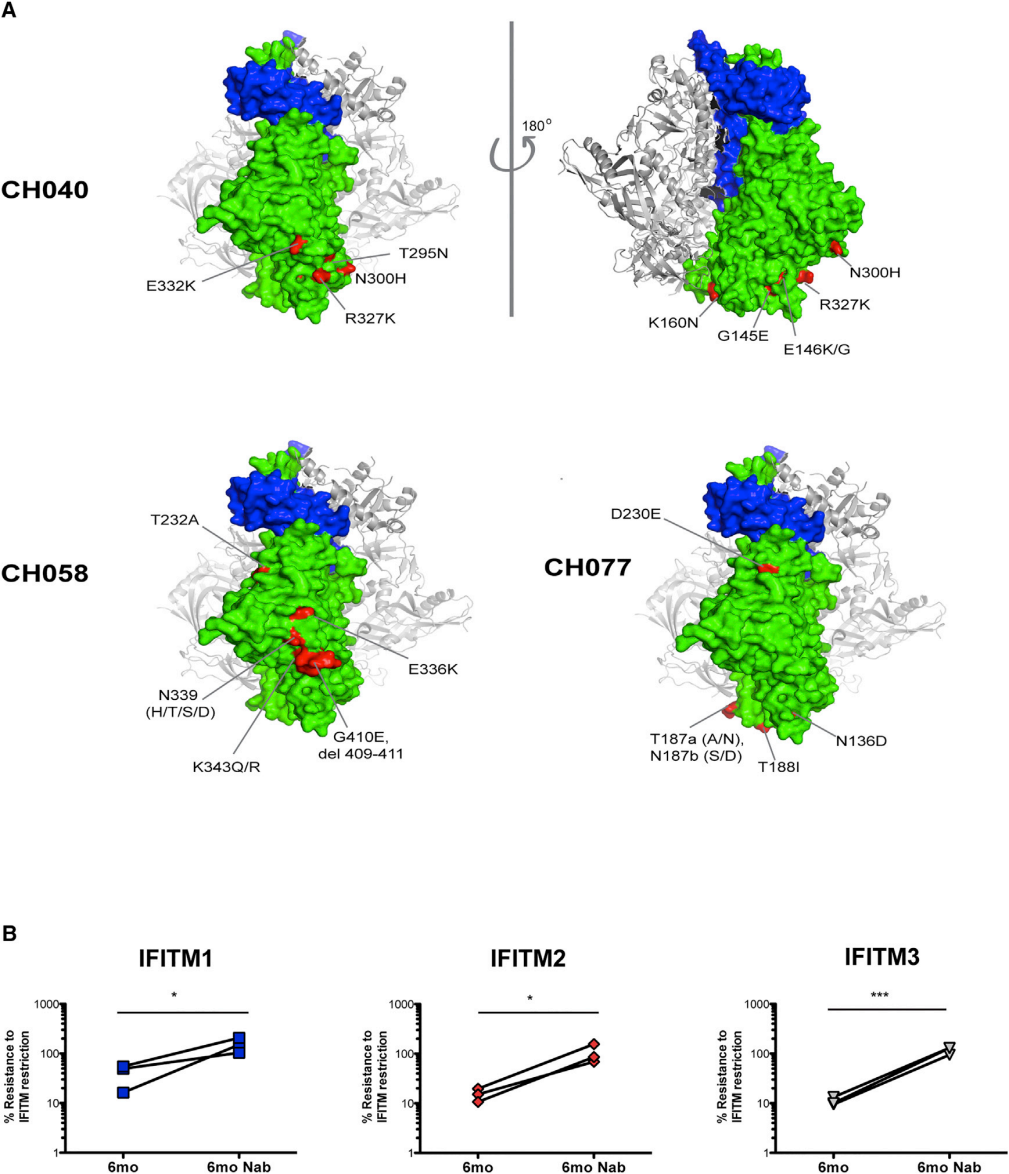
Reversion of Neutralization Escape Mutants in 6-Month Viruses Restores IFITM Resistance (A) The gp120:gp41 trimer structures show the NAb escape mutations of CH040, CH058, and CH077 in red. Gp120, green; gp41, blue. Images were drawn using PDB: 5ACO in Pymol. (B) U87/CD4/CCR5 IFITM cells were infected with the indicated 6-month chronic viruses or those containing NAb escape reversion mutations at an MOI of 0.05. Cumulative replication over a period of 96 hr was assayed for viral production on HeLa-TZMbl indicator cells as in Figures 3A and 3B. Statistics were performed using a paired Mann-Whitney test (^∗∗∗^p < 0.001; ^∗∗^p < 0.01; ^∗^p < 0.05; ns, p > 0.05). See also Table S2.

While there were no common amino acid changes in Env that were shared between the various TF/6-month pairs, most differences mapped to the external surfaces of the trimers and the variable loops (Figure S3C; Table S1). This raised the possibility that adaptive changes in Env that accrued in response to host immune responses during the course of the infection might explain the increased sensitivities to IFITMs 2 and 3. In three TF/6-month pairs, amino acid positions in the 6-month envelope previously have been identified to mediate evasion of early autologous neutralizing antibody (NAb) responses (Figure 4A; Table S2) (Bar et al., 2012). Importantly, experimental reversion of these amino acids in the 6-month molecular clones completely restored the IFITM resistance (Figure 4B).

**Figure 5:**
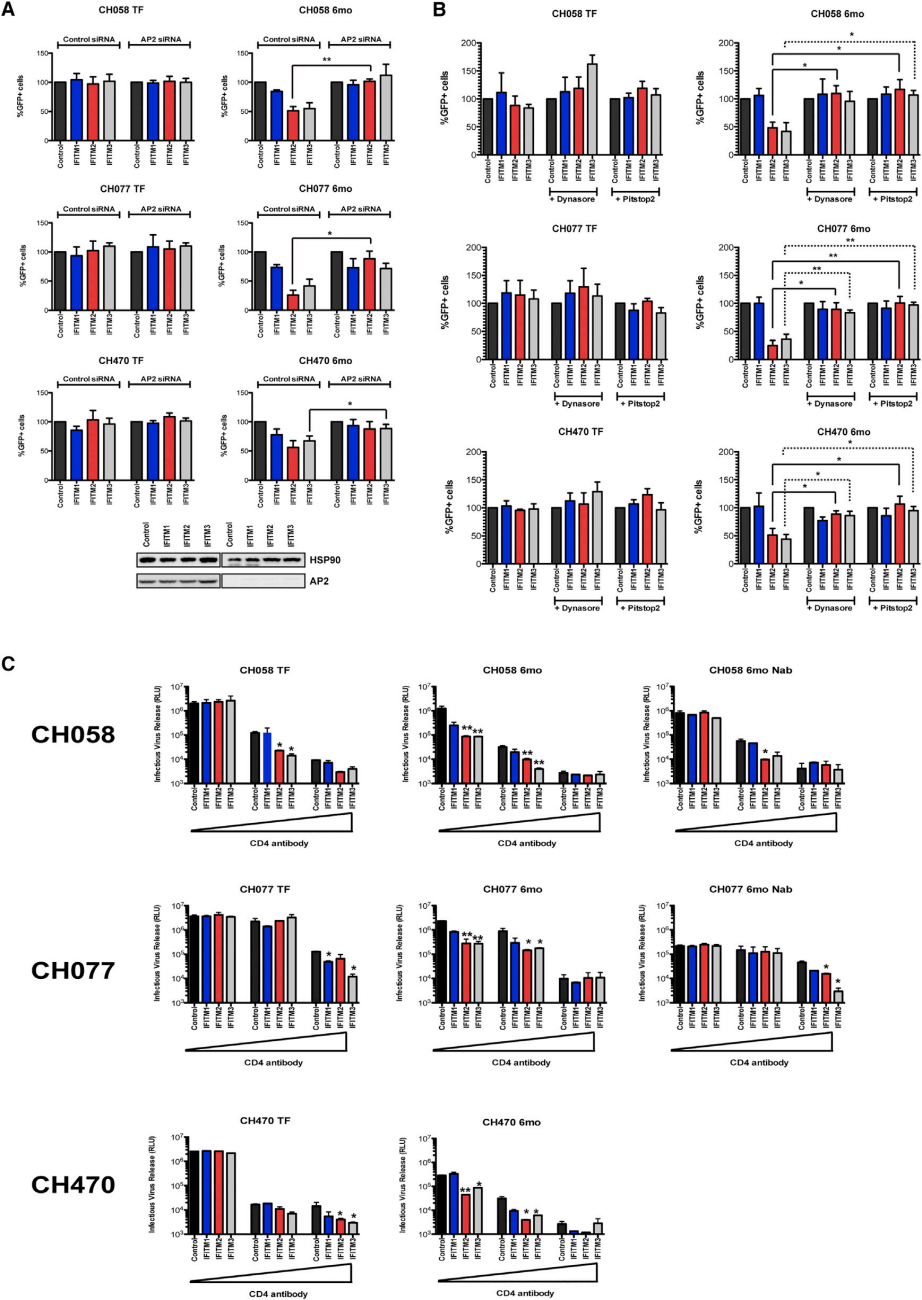
Effect of Endocytosis Inhibitors and Surface CD4 Levels on IFITM Restriction of TF and 6-Month Viral Entry (A) U87/CD4/CCR5 IFITM cells were treated with control or AP-2-specific siRNAs for 48 hr and then infected with the indicated HIV-1-pseudotyped vector. GFP^+^ cells were analyzed as in Figure 1A (^∗∗∗^p < 0.001; ^∗∗^p < 0.01; ^∗^p < 0.05; ns, p > 0.05, unpaired two-tailed test). Knockdown was assessed by western blot for AP-2μ. (B) As in (A) but the cells were pretreated with DMSO, dynasore, or Pitstop2. (C) U87/CD4/CCR5 IFITM-expressing cells were infected with TF viruses and their matched chronic pairs at an MOI of 0.5 in the presence of 0, 10, or 100 ng/mL of CD4-blocking antibody SK3 for 6 hr. Then 48 hr post-infection, virus production was measured by infection of HeLa-TZMbl indicator cells (^∗∗^p < 0.01; ^∗^p < 0.05; ns, p > 0.05, unpaired two-tailed t test). All error bars represent ± SEM (n = 3). See also Figure S4.

The resistance of the 6-month viruses to surface-retained IFITM2 or 3 (Figure S3B) prompted us to seek evidence that alterations in viral entry modulated by receptor engagement could explain the increased IFITM sensitivities of the 6-month variants. First we found that the sensitivity of the 6-month viruses to IFITM2 or 3 could be abolished by AP-2 depletion (Figures 5A and S4A) or treatment of the cells with endocytosis inhibitors (Figures 5B and S4B). Since all the viruses were R5 tropic, we then compared TF/6-month pairs in the presence of limiting surface CD4 density with a blocking antibody. Intriguingly, for CH077, CH058, and CH470 TF clones and the corresponding NAb revertants, an IFITM2/3 restriction phenotype similar to the 6-month variant could be induced by limiting CD4 levels (Figure 5C). Conversely, the 6-month variants themselves maintained their restriction profile irrespective of entry inhibition by CD4 neutralization. Together these data argue that the primary engagement with CD4 at the plasma membrane is a major determinant of IFITM resistance in the TF viruses and the accrual of amino acid variations under immune pressure impacts on the cellular route of entry, leading to IFITM2 and 3 restriction.

### IFITM Depletion in CD4^+^ T Cells Rescues 6-Month Viral Isolates from IFN-1

A major challenge with ISGs that restrict HIV-1 in cultured cells is demonstrating that they have relevance in primary targets. The reported acquisition of IFN-1 sensitivity in the 6-month viruses (Fenton-May et al., 2013) prompted us to determine whether IFITMs were contributing factors. We first determined the localization of IFITMs in Jurkat T cells transduced with the same HA-tagged constructs used for microscopy in our U87 cells (Figures S5A and S5B). As in the U87 cells, IFITM1 was almost exclusively at the plasma membrane. Aside from some minor surface labeling, IFITM3 was found in intracellular endosomes. IFITM2 could be detected at the plasma membrane, but, upon permeablization, 3- to 4-fold more was stained in flow cytometry, indicating that the majority localization was again intracellular.

**Figure 6:**
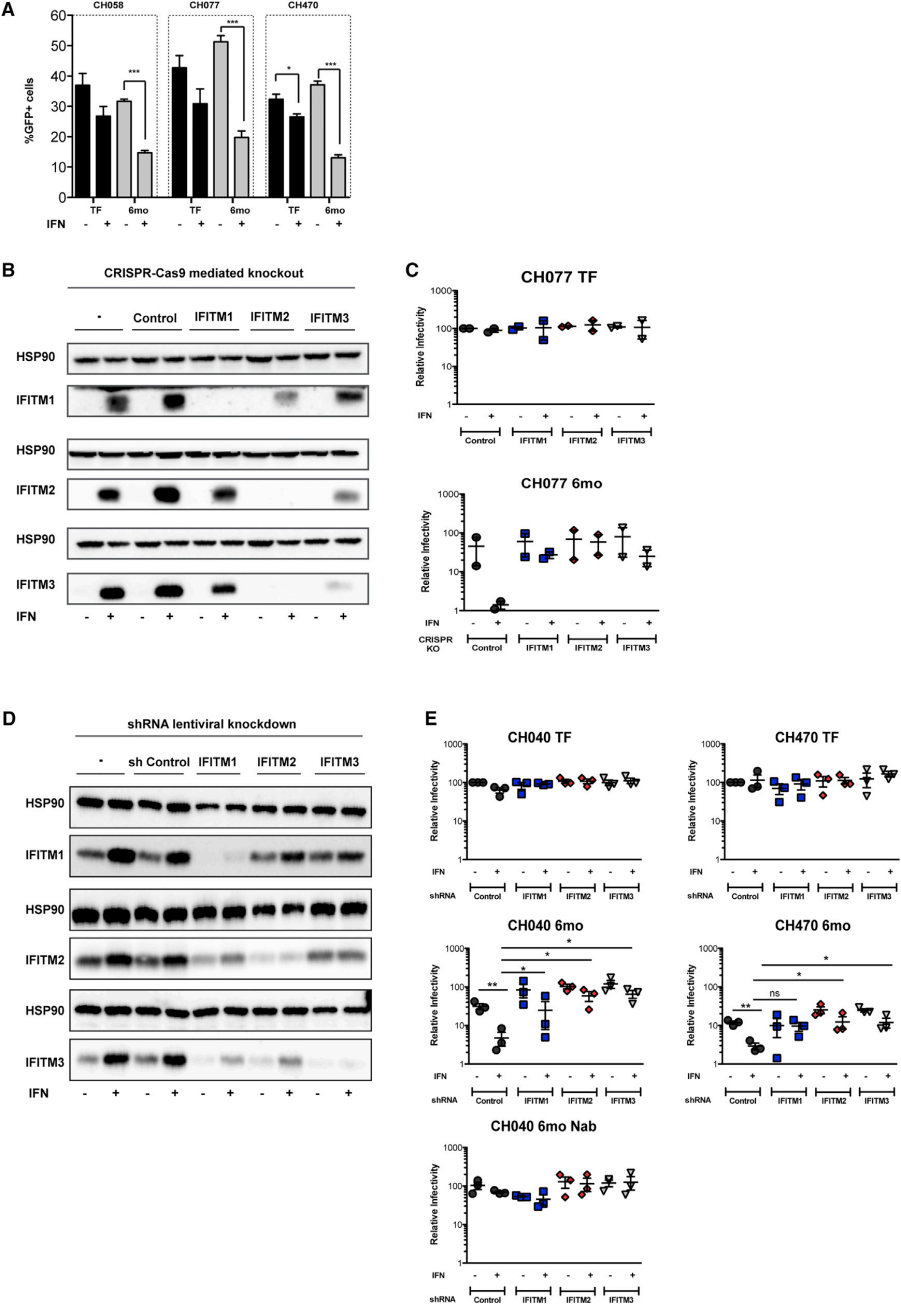
IFITM2 and IFITM3, in Particular, Contribute to the Inhibition of HIV-1 Replication in Primary CD4^+^ T Cells (A) HIV-1 vectors pseudotyped with TF and 6-month envelope variants were used to challenge U87/CD4/CCR5 cells that had been pretreated overnight with 1,000 U/mL universal IFN-1. Infected cells were analyzed by flow cytometry 48 hr later (^∗^p < 0.05; unpaired; unpaired two-tailed t test). (B) U87/CD4/CCR5 cells were transduced with IFITM-specific or control lentiCRISPR-p2A-GFP vectors sufficiently to achieve 80%–98% GFP^+^. The efficiency of CRISPR knockout of IFITM expression, in the absence or presence of IFN-1 (1,000 U/mL), was determined by western blotting. HSP90 served as a loading control. (C) Cells from (B) were challenged with CH077 at an MOI of 0.05 in the presence of absence of 1,000 U/mL IFN-1, and cumulative replication at 96 hr was determined as previously described. (D) Primary CD4^+^ T cells were transduced with shRNAs targeting IFITM1, 2, or 3 or a control shRNA and cultured for 72 hr. Transduced cells were cultured with or without 500 U/mL IFN-1 for 24 hr before determining the efficiency of IFITM knockdown by western blot analysis. HSP90 served as a loading control. (E) CD4^+^ T cells, expressing control or IFITM-specific shRNAs, were infected with the indicated TF and chronic matched-pair viruses at an MOI of 0.05. A time course of replication, in the presence or absence of 500 U/mL universal IFN-1, was assayed with supernatants harvested every 24 hr for a total of 5 days. Infectious virus was determined by infection of HeLa-TZMbl indicator cells. Data shown are representative of the replication values at 96 hr, and IFITM shRNA knockdown data are expressed relative to the virus-specific TF control sample. Data shown are representative of three independent donors and independent experiments. Statistical significance between indicated pairs was determined using an unpaired two-tailed t test (^∗∗^p < 0.01; ^∗^p < 0.05; ns, p > 0.05). All error bars represent ± SEM (n = 3). See also Figure S5.

To assess the contribution of IFITMs to HIV-1 IFN-1 sensitivity, lentiviral vectors pseudotyped with TF or 6-month envs were used to transduce U87/CD4/CCR5 after pretreatment overnight with 500 U IFN-1. The 6-month envs displayed a greater reduction in one-round infectivity after IFN-1 treatment, indicating that the envelopes contained an IFN sensitivity determinant (Figure 6A). We next employed CRISPR-Cas9 lentiviral vectors targeting individual IFITMs or an irrelevant control (luciferase). While each guide effectively knocked out the specific IFITM targeted, the degree of homology between them and, presumably, their chromosomal positioning and proximity resulted in effective deletion of IFITM2 by IFITM3 guides and vice versa, as well as reduced expression of both IFITMs 2 and 3 by the IFITM1 guide (Figure 6B). Using a TF/6-month pair, CH077, that only displayed IFITM2 and 3 sensitivity, we found that, in U87/CD4/CCR5 cells, only the 6-month variant displayed IFN-1 sensitivity (Figure 6C). Moreover, treatment with the IFITM CRISPR guides relieved this IFN-induced restriction, with no effect on the TF virus. This indicates that expression of IFITMs contributes to the different IFN sensitivities of TF and 6-month virus.

We next moved into primary human CD4^+^ T cells using lentivirally delivered small hairpin RNAs (shRNAs). Again, due to their high degree of homology, effective shRNAs displayed a degree of cross-knockdown, particularly between IFITMs 2 and 3 (Figure 6D). However, using cells from three independent donors, we examined cumulative replication of CH040 and CH470 after 5 days in the presence or absence of 500 U/mL universal IFN-1. The TF variants of both viruses displayed a weak sensitivity to IFN-1 treatment, with little or no effect of any of the shRNAs. In contrast, replication of the 6-month variants was markedly reduced by IFN (Figure 6E). In the case of CH040, this IFN sensitivity was rescued by shRNAs targeting IFITMs 2 or 3, but not IFITM1, consistent with the replication of this virus in our engineered cells (Figure 3). For CH470, knockdown of IFITM1 also contributed to the rescue. Importantly, for CH040, the NAb revertant virus behaved like the TF virus, which is IFN resistant and unresponsive to IFITM knockdown (Figure 6E). Taken together, these data demonstrate that IFITMs are relevant HIV-1 antiviral factors in primary human cells and substantially contribute to the IFN-1 sensitivity of chronic phase viruses in a manner dependent on escape mutations of the envelope to host immune responses. These data also suggest that IFITM resistance in TF viruses is an important attribute in vivo.

## Discussion

We have re-examined the roles of IFITMs in the restriction of HIV-1 entry, and we present evidence that is consistent with them having a role in the innate immune response against the virus during the earliest stages of acute HIV-1 infection. Specifically, we found that TF HIV-1 is IFITM resistant but that the virus acquires sensitivity progressively over time. This increased IFITM sensitivity correlates with the acquisition of Env mutations previously shown to mediate escape from early autologous neutralization (Bar et al., 2012), and, thus, it explains at least some of the IFN-1 sensitivity previously shown to emerge during chronic infection (Fenton-May et al., 2013). Additionally, we document that the patterns of IFITM sensitivity of chronic and lab-adapted isolates of HIV-1 are determined by their co-receptor use and the subcellular localization of the IFITM itself. These data strongly suggest that co-receptor usage affects the route of cellular entry such that the virus fuses in different subcellular compartments.

The mechanism of action of IFITMs remains poorly understood. For most pH-dependent viruses, IFITM3 in particular induces the accumulation of virions in late endosomes because of a lack of proper envelope-mediated fusion. This has been proposed to be as a result of IFITM3 interaction with VAMP-associated protein (VAPA) leading to the dysregulation of sterol trafficking (Amini-Bavil-Olyaee et al., 2013), although this has yet to be confirmed by others. In keeping with the notion of membrane modification, the antifungal drug amphotericin B, which modulates cholesterol synthesis, inhibits IFITM3 restriction of IAV (Lin et al., 2013). By contrast, the effects of IFITMs on HIV-1 have been less well defined. While all have been shown to have effects on viral infectivity (Lu et al., 2011), the small differences observed in one-round infections have led to speculations of altered envelope processing/incorporation in the producer cells (Yu et al., 2015) or viral membrane incorporation (Compton et al., 2014, Tartour et al., 2014). In our cell lines expressing physiologically relevant IFITM levels, we saw no envelope-processing defects, and, despite the IFITM incorporation into nascent virions, the infectivity phenotypes we observed correlate only with the expression/localization of the IFITM in the target cell.

The dependence of viral restriction on IFITM localization has important implications for HIV-1 entry and tropism. The sequential engagement of CD4 and co-receptor leads to complex conformational changes in Env that activate membrane fusion (Wilen et al., 2012). Since these events are pH independent, it is widely assumed that fusion takes place at the plasma membrane. However, it has been proposed that HIV-1 enters by dynamin-dependent endocytic processes (Miyauchi et al., 2009). This is countered by observations that, while such entry may be observable, the endocytosis is not necessary for productive infection (Herold et al., 2014, Pelchen-Matthews et al., 1995). The restriction of X4 and some R5 viruses by IFITM2 or IFITM3, which is dependent on the AP-2-binding site, suggests that the subcellular location of viral entry among different HIV-1 strains is variable. These observations do not necessarily mean that all IFITM2/3-sensitive viruses fuse in endosomes. Both IFITMs 2 and 3 accumulate in endosomes after AP-2-mediated internalization from the plasma membrane, and so there will always be a pool at the cell surface. However, they will be associated with endocytic domains such as clathrin-coated pits; by contrast, the tyrosine-mutated IFITM2/3 will not be. Therefore, the pattern of IFITM restriction implies that different viruses enter cells at spatially distinct localizations. That this is dependent on X4 usage or CD4 density (in the case of the 6-month viruses) suggests that receptor engagement and/or trafficking is a prime determinant of entry site. It is known that CCR5 and CXCR4 traffic differently when engaged in ligand-dependent signaling (Signoret et al., 1998), and there is much suggestive evidence that co-receptor engagement by incoming HIV-1 also induces signaling (Wu and Yoder, 2009). Interestingly, the entry of the R5 user YU2 could be rescued from IFITM1 by endocytic blockade, indicating that modulation of receptor trafficking in the plane of the plasma membrane may affect viral entry site at the cell surface. Our data therefore suggest that the patterns of IFITM restriction can be used to dissect the role of endocytic trafficking in HIV-1 entry, which has important implications for understanding cell tropism and co-receptor switching.

Unlike the restriction factors APOBEC3G, tetherin, and SAMHD1, which are directly counteracted by lentiviral-encoded accessory proteins, there is no direct evidence for the relevance of any other specific ISG-mediated restriction in vivo, despite abundant evidence that many can target specific stages of the replication cycle in cultured systems (Doyle et al., 2015). However, the acquisition of IFN-1 sensitivity by chronic viruses is highly suggestive that some must do so at physiological expression levels (Fenton-May et al., 2013, Parrish et al., 2013). ISGs that are directly antiviral often target viral structures (the cell-derived membrane) or processes (reverse transcription), which cannot simply be mutated (Doyle et al., 2015). As such, the entry pathway of the virus is a perfect target for the host.

The most striking observation in our study is that, while we see varying IFITM sensitivities for lab-adapted and chronic strains of HIV-1, the TF virus envelope proteins are almost uniformly resistant to their activities. The acquisition of sensitivity to IFITMs over the ensuing months strongly suggests that their avoidance is a requirement for HIV-1 to be successfully transmitted. The mapping to residues exposed on the outer faces of the envelope trimers and the timing of the acquisition itself suggest that immune escape mutations might be driving IFITM sensitivity. Broadly NAbs are generally observed only after several years of infection (Burton and Mascola, 2015). However, complement-fixing anti-Env antibodies are detectable coincident with the onset of T cell immunity (Aasa-Chapman et al., 2004, Aasa-Chapman et al., 2005). Furthermore, while exhibiting limited breadth, autologous neutralizing responses in the first 6 months exert sufficient pressure on the envelope to select for escape variants that become fixed in the viral population (Bar et al., 2012, Moody et al., 2015). Any adaptive mutation in a given viral protein may lead to a functional change. NAbs in particular can block HIV-1 receptor engagement (i.e., blocking the CD4-binding site) or can prevent receptor-binding-induced structural changes required for cell fusion (exemplified by MPER-binding Abs) (Burton and Mascola, 2015). Thus, escape from NAbs may impact on these receptor-driven rearrangements and, by extension, the route of cellular entry as we saw for the 6-month viruses.

In keeping with the above, Envs from chronic viruses have been shown to be less sensitive to CD4bs NAbs and to exhibit differences in CCR5 engagement compared to Envs from TF viruses (Wilen et al., 2011). Whether IFITM sensitivity is a general feature of NAb escape or specific to only certain epitopes and whether these immune pressures select for additional compensatory changes require further study. It has been demonstrated that TF viruses have ∼1.5-fold more virion-associated Env than chronic viral isolates (Parrish et al., 2013). Whether this is a reflection of increased virion incorporation or trimer stability is not clear. Since IFITM sensitivity is modulated by CD4 engagement, it is possible that Env density is a contributing factor to IFITM resistance. However, previous studies of fusion kinetics between TF and chronic envelopes have not found a significant difference (Wilen et al., 2011). Furthermore, while not analyzed here, we cannot exclude the possibility that Env escape mutations in response to CD8+ T cell pressure in the same patients (Liu et al., 2013) could not have also had a structural impact that led to IFITM sensitivity.

TF viruses entering a naive host do so in the absence of pre-existing adaptive immunity, but they must avoid innate responses to allow sufficient time to establish a systemic infection. Thus, efficient cell entry into activated mucosal CD4^+^ T cells is an absolute requirement for the virus. We suggest that viruses that successfully transmit are those that can fuse efficiently at the cell surface of cells expressing high CD4 levels. This would endow such viruses with a relative resistance against IFN-induced factors such as IFITMs, and it would particularly facilitate cell-to-cell spread in the face of the robust IFN-1 response that occurs during acute infection (Stacey et al., 2009). However, these requirements change during chronic infection when the virus has to escape from pressures by the adaptive immune system.

Amino acid changes in Env that allow escape from such adaptive responses may impact envelope function in such a way that viral entry routes and, thus, restriction by IFITMs may change. It is interesting to note that for three TF/6-month pairs used here, NAb escape mutations were associated with a reduced ability to replicate in CD4 T cells (Bar et al., 2012). Since activated CD4 T cells express at least some IFITMs, it may well be that fitness costs incurred by immune escape mutations reflect the associated loss of effectively counteracting antiviral restriction. In the case of the TF/6-month pairs tested here, IFITM restriction in primary CD4^+^ T cells correlates with the IFN sensitivity of the chronic virus. This would argue that, after the establishment of chronic infection, the selective pressure of factors such as IFITMs is no longer sufficient to impact viral replication or, more likely, the counter-selection by adaptive immune responses is so strong that the effects of IFN-1-induced factors like IFITMs on viral replication become tolerable.

Interestingly, there is evidence that IFN resistance increases again during the late stages of HIV-1 infection (Fenton-May et al., 2013). Understanding whether this inversely correlates with the strength of the adaptive immune response will be particularly interesting. We therefore propose that IFITM restriction and its inverse relationship with adaptive immune escape might be a paradigm for demonstrating that a given ISG that inhibits HIV-1 in vitro is likely to be of relevance in vivo.

Our data indicate that IFITMs represent major effectors of the innate immune response to HIV-1 in vivo. Because of their sequence similarity, we were unable to find potent shRNAs that could selectively knockdown endogenous IFITMs individually in primary cells. However, the patterns of restriction observed in ectopically expressing cell lines would suggest that the endosomal IFITMs 2 and 3 are the most contributing to the IFN sensitivity of chronic viruses. The rs12252-C polymorphism in IFITM3 is predicted to result in a variant lacking the tyrosine-based endocytic signal. This potentially would lead to surface expression of IFITM3 (Everitt et al., 2012). Recently this polymorphism was associated with faster disease HIV-1 progression in China (Zhang et al., 2015). Interestingly, there was no enhanced susceptibility to infection between different *Ifitm3* genotypes. This observation is entirely consistent with our data that both TF and 6-month viruses are resistant to surface IFITM3. We predict that the viruses that escape Env-directed adaptive immune responses in subjects carrying the rs12215-C polymorphism will be less susceptible to host innate restriction, enhancing their replication and therefore disease progression.

## Experimental Procedures

### Plasmids and Reagents

Full details of HIV-1 molecular clones, HIV-1 Env plasmids, and IFITM expression constructs are described in the Supplemental Experimental Procedures. Human IFITM1, IFITM2, and IFITM3 were cloned into pLHCX retroviral vector (Clontech Laboratories). Mutants IFITM2-Y19F and IFITM3-Y20F were generated by site-directed mutagenesis using the parental pLHCX IFITM1, 2, or 3 constructs as templates. IFITM1, IFITM2, IFITM3, and Y19F or Y20F mutants thereof were all HA tagged by PCR-based mutagenesis, again using the parental pLHCX-IFITM1, 2, or 3 as templates.

### Cell Culture

The 293T-, HeLa-TZMbl-, and U87-based cell lines were cultured, transfected, or infected as described in the Supplemental Experimental Procedures. Human primary CD4^+^ T cells were isolated from peripheral blood mononuclear cells (PBMCs) of healthy human donors and cultured and infected as outlined in the Supplemental Experimental Procedures.

### Virus and HIV-1 Env Pseudotyped Viral Vector Production

To generate virus and vector stocks, 293T cells were transfected with 10 μg HIV-1 molecular clones plasmid or three-plasmid mix of pCSGW (GFP-encoding vector genome), pCRV1-HIV-1 GagPol packaging vector, and pSVIII or pCRV1 plasmids encoding various HIV-1 envelope glycoproteins. Supernatants were harvested and filtered 48 hr post-transfection and titers were calculated by standard methods. Full details are given in the Supplemental Experimental Procedures.

### shRNA Lentiviral Knockdown and CRISPR Knockout of IFITM Expression

Silencing of IFITM expression was mediated either by lentiviral shRNA knockdown in primary human CD4^+^ T cells or CRISPR knockout in U87 CD4^+^ CCR5^+^ cells, as outlined in the Supplemental Experimental Procedures.

### Infections

U87/CD4/CXCR4^+^ or U87/CD4/CCR5 cells stably expressing IFITMs 1, 2, or 3 or mutants thereof were infected with the indicated HIV-1 molecular clone at an MOI of 0.05. Media were replaced 8 hr post-infection, and culture supernatants were harvested every 24 hr post-infection for a total of 120 hr. Infectious viral release was determined by infecting HeLa-TZMbl indicator cells and 48 hr post-infection assaying for virus release by measuring chemiluminescent β-galactosidase activity, using the Tropix Galacto-Star system (Applied Biosystems) according to the manufacturer’s instructions. For one-round virus release assays, cells were infected with the indicated HIV-1 molecular clone at an MOI of 0.5. Viral production was measured for supernatants harvested at 48 hr post-infection on HeLa-TZMbl indicator cells, as above. For env-pseudotyped viral vector entry assays, the same cells were infected with a fixed dose of HIV-1 viral vectors at an MOI of 0.2 for 48–72 hr prior to analysis for GFP expression by flow cytometry.

Activated CD4^+^ T cells, transduced with the appropriate shRNA lentiviral vectors, were infected at an MOI of 0.1; then 8–12 hr post-infection, media were replaced. Supernatants were harvested every 72, 120, and 168 hr post-infection, and virus particle production was assessed on HeLa-TZMbl cells as described previously.

### CD4 Competition Assays

U87-MG CD4^+^ CCR5^+^ cells were infected at an MOI of 0.5 with the indicated HIV-1 molecular clone/anti-human CD4 (SK3 clone, BioLegend) antibody mix. Anti-human CD4 antibody was used at concentrations of 100, 10, and 0 ng/mL. Then 48 hr post-infection, supernatants were harvested and used to infect HeLa-TZMbl cells, assaying for any dose-dependent reduction in virus release as detailed in the Supplemental Experimental Procedures.

### Ethics Statement

Ethical approval to use blood from healthy donors was granted by King’s College London Infectious Disease BioBank Local Research Ethics Committee (under the authority of the Southampton and South West Hampshire Research Ethics Committee—approval REC09/H0504/39), approval number SN-1/6/7/9.

## Author Contributions

All experiments were performed by T.L.F. with the help of H.W. and K.C. S.S.I., K.D., S.S., P.K., P.B., A.F., and B.H.H. provided reagents, data, and advice. T.L.F. and S.J.D.N. analyzed the data and wrote the manuscript.

## Supplemental Information

Document S1. Supplemental Experimental Procedures, Figures S1–S5, and Tables S1 and S2 Document S1. Supplemental Experimental Procedures, Figures S1–S5, and Tables S1 and S2

Document S2. Article plus Supplemental Information Document S2. Article plus Supplemental Information
