# Supplementary material for: Resistance of Transmitted Founder HIV-1 to IFITM-Mediated Restriction
Source: Cell Host Microbe. 2016 Oct 12;20(4):429–42. doi: 10.1016/j.chom.2016.08.006 (PMC5075283; doi:10.1016/j.chom.2016.08.006)
Supplement: Document S1. Supplemental Experimental Procedures, Figures S1–S5, and Tables S1 and S2 [file mmc1.pdf]

Cell Host & Microbe, Volume *20*

Supplemental Information

Resistance of Transmitted Founder HIV-1 to IFITM-Mediated Restriction

Toshana L. Foster, Harry Wilson, Shilpa S. Iyer, Karen Coss, Katie Doores, Sarah Smith, Paul Kellam, Andrés Finzi, Persephone Borrow, Beatrice H. Hahn, and Stuart J.D. Neil

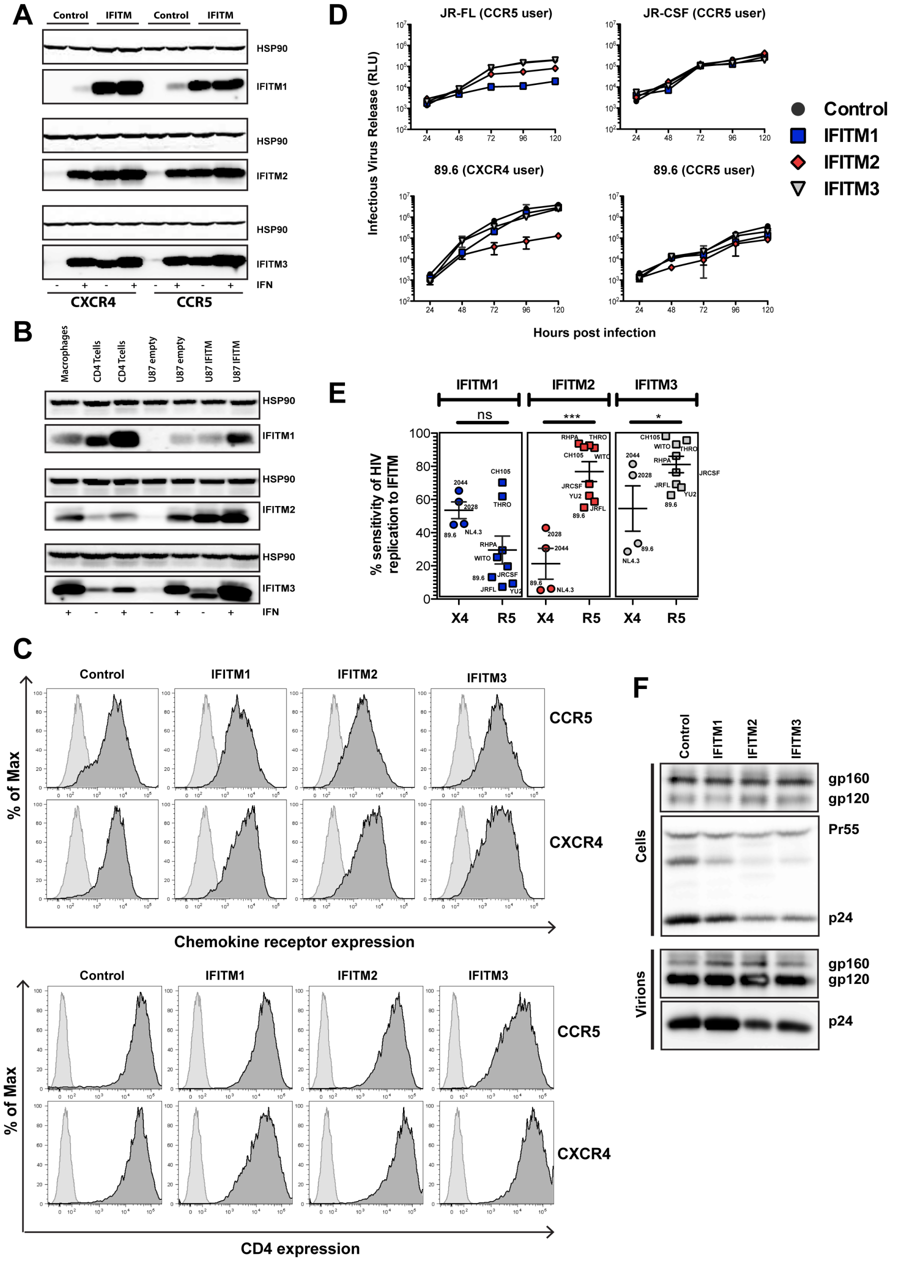

Figure S1, related to Figure 1. IFITM proteins impair HIV-1 infection.

(A) U87 cell lines that stably express CD4 and CCR5 or CXCR4 were transduced with IFITM1, 2 or 3. Control represents cells that were transduced with empty lentiviral vector pLHCX. Expression levels of IFITMs in the absence or presence of 1,000 U/ml interferon-α were determined by western blotting. HSP90 served as a loading control.

(B) The expression levels of IFITMs I stably transduced U87 cells was compared to that of interferon-α (500 U/ml) induced primary human macrophages and CD4+ T-cells. HSP90 served as a loading control.

(C) IFITM proteins do not affect the expression of the entry receptors CD4, CCR5 or CXCR4. Representative data of CCR5 or CXCR4 (upper panel) and CD4 (lower panel) surface expression in the absence or presence of IFITM proteins. Isotype control is in light grey. U87 cells stably expressing the entry receptors and the control empty vector or IFITM proteins were analysed for CD4 and chemokine receptor surface expression (dark grey) by flow cytometry.

(D) Virus replication of different virus isolates in the presence of IFITM proteins in U87/CD4/CoR cells. Data represent a summary of three independent experiments (see also Figure 1B).

(E) Panel as in Figure 1B with viral isolates annotated.

(F) Purified virus particles and cell lysates from U87 $CD4^{+}$-$CXCR4^{+}$ IFITM-expressing cells infected with proviral HIV-1 NL4.3 at an MOI of 0.5 were analysed for differences in envelope processing efficiency by immunoblotting using anti-gp120 and anti-p24.

A

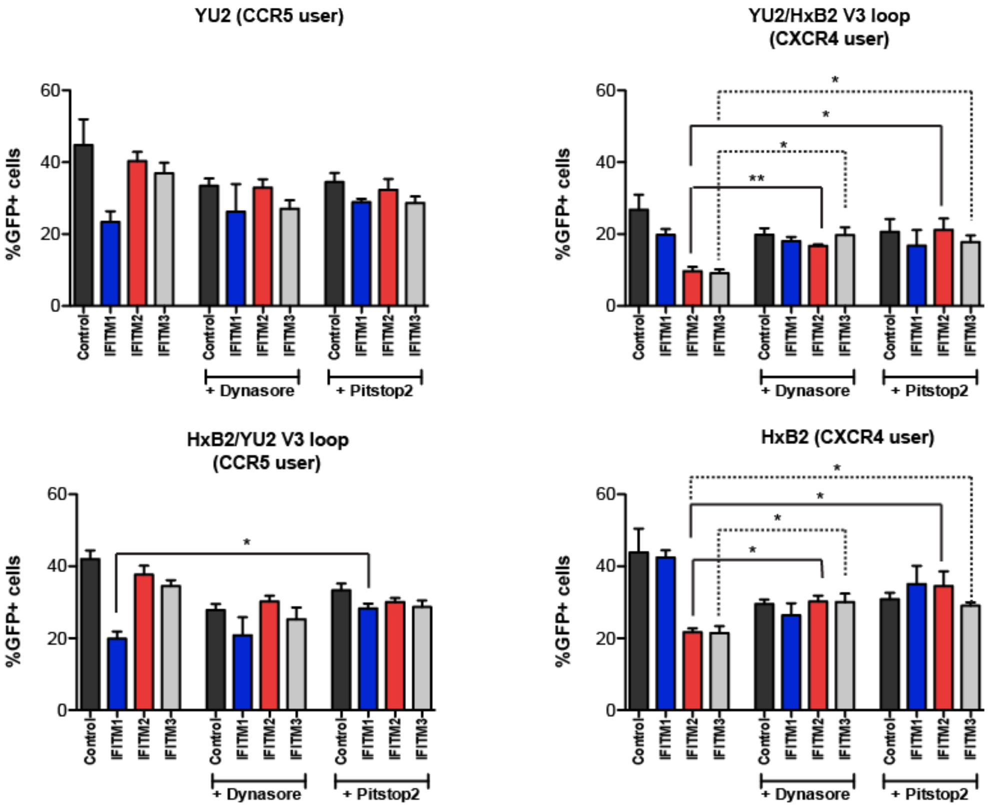

B

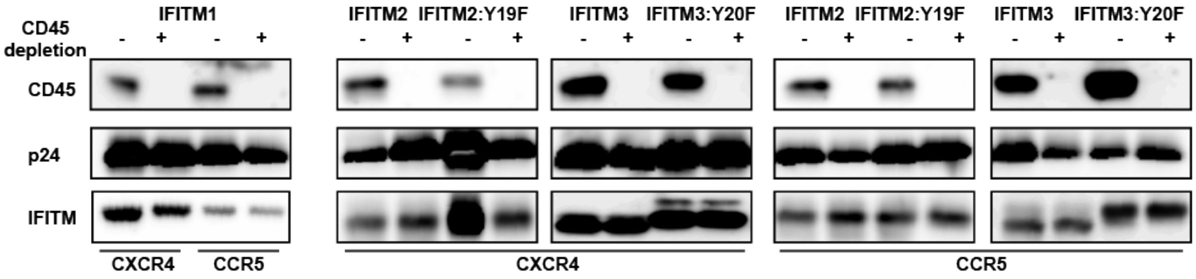

Figure S2, related to Figure 2. IFITM proteins are incorporated into HIV-1 virions but incorporation does not correlate with inhibition phenotypes observed.

(A) Absolute values for data shown in Figure 2F. All error bars represent ±SEM. *** $p<0.001$, ** $p<0.01$, *$p<0.05$, ns $p>0.05$; unpaired two-tailed test.

(B) U87 $CD4^+$-$CXCR4^+$ or $CD4^+$-$CCR5^+$ cells overexpressing the HA-tagged wild-type IFITM proteins or the mutants IFITM2-Y19F or IFITM3-Y20F were infected with 89.6 provirus. Viral supernatants were harvested and purified through a 20% sucrose cushion before CD45 depletion was conducted to remove exosomes on p24 matched samples. Western blotting was performed using anti-HA (to detect IFITMs), anti-p24 or anti-CD45.

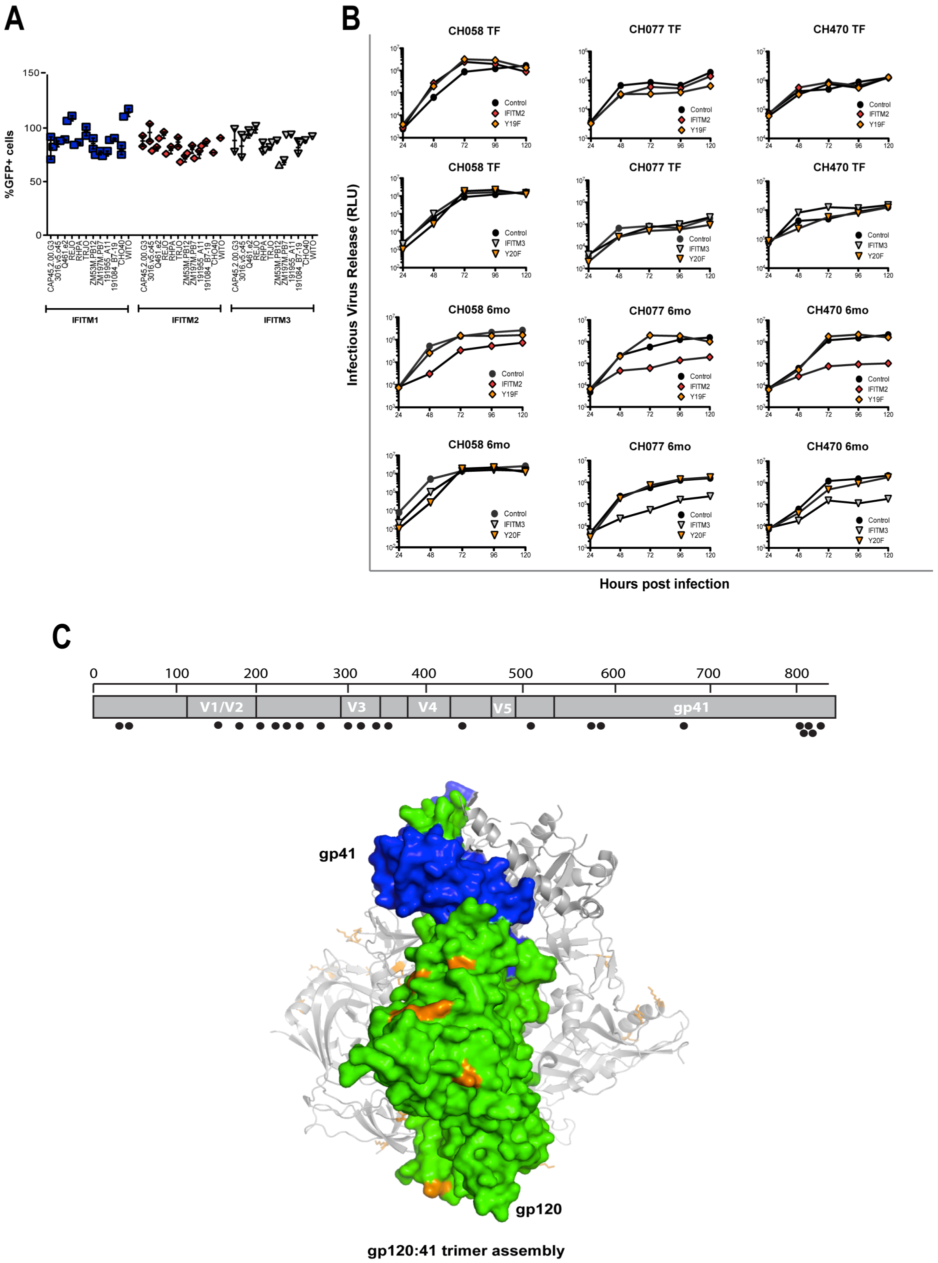

Figure S3, related to Figure 3. Gain in sensitivity to IFITM inhibition correlates with amino acid changes in T/F versus 6mo chronic pairs.

(A) Envelope pseudotyped HIV-1 vectors were produced for the following viruses: CAP45.2.00.G3, 3016.v5.c45, Q461.e2, REJO, RHPA, TRJO, ZM53M.PB12, ZM197M.PB7, 191955_A11, 191084_B7-19, CH040, WITO. U87 $CD4^{+}$-$CCR5^{+}$ stably expressing the IFITM 1, 2 or 3 proteins were infected for 48hrs before the percentage of infected cells was determined by flow cytometry.

(B) The replication phenotypes of TF and 6-mo chronic clones were determined by infection of U87 $CD4^{+}$-$CCR5^{+}$ stably expressing the wild-type IFITM 2 or 3 proteins as well as the mutants IFITM2-Y19F or IFITM3-Y20F. A time course of replication over a 5 day period was assayed, with supernatants harvested every 24 hrs. Infectious virus production was determined by infection of HeLa-TZMbl indicator cells.

(C) (Top) Diagrammatic representation of the amino acid differences between the envelope of the TF and 6-mo chronic consensus clones analyzed in this study, i.e. CH040, CH058, CH077, CH236, CH470 and CH850, based on Hxb2 reference sequencing numbering. Dots indicate amino acid position.

(Bottom) Cartoon representation (left) of the top view of the gp120:gp41 trimer. Gp120 is shown in green and gp41 in blue. Amino acid differences between the envelope of the TF and 6-mo clones as in Figure S3A are indicated in orange. The other gp120 and gp41 monomers that comprise the env trimer are shown in grey. Images were drawn using PDB code 5ACO in Pymol.

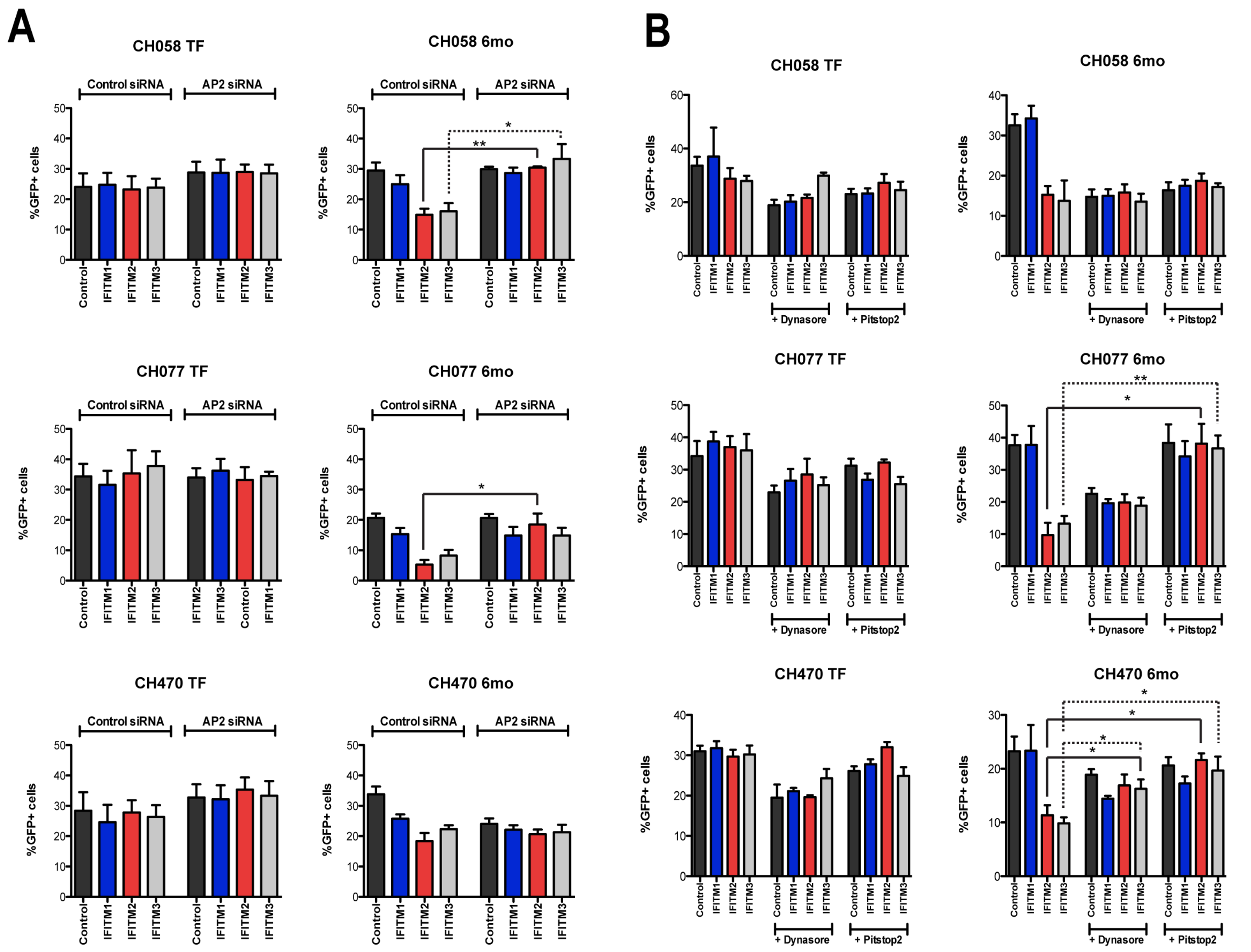

Figure S4, related to Figure 5. Blocking endocytosis by AP2 knockdown and by the use of inhibitors rescues IFITM restriction of 6mo chronic virus isolates.

(A) Absolute values for data shown in Figure 5A. All error bars represent ±SEM. *** p<0.001, ** p<0.01, *p<0.05, ns p>0.05; unpaired two-tailed test.

(B) Absolute values for data shown in Figure 5B. All error bars represent ±SEM. *** p<0.001, ** p<0.01, *p<0.05, ns p>0.05; unpaired two-tailed test.

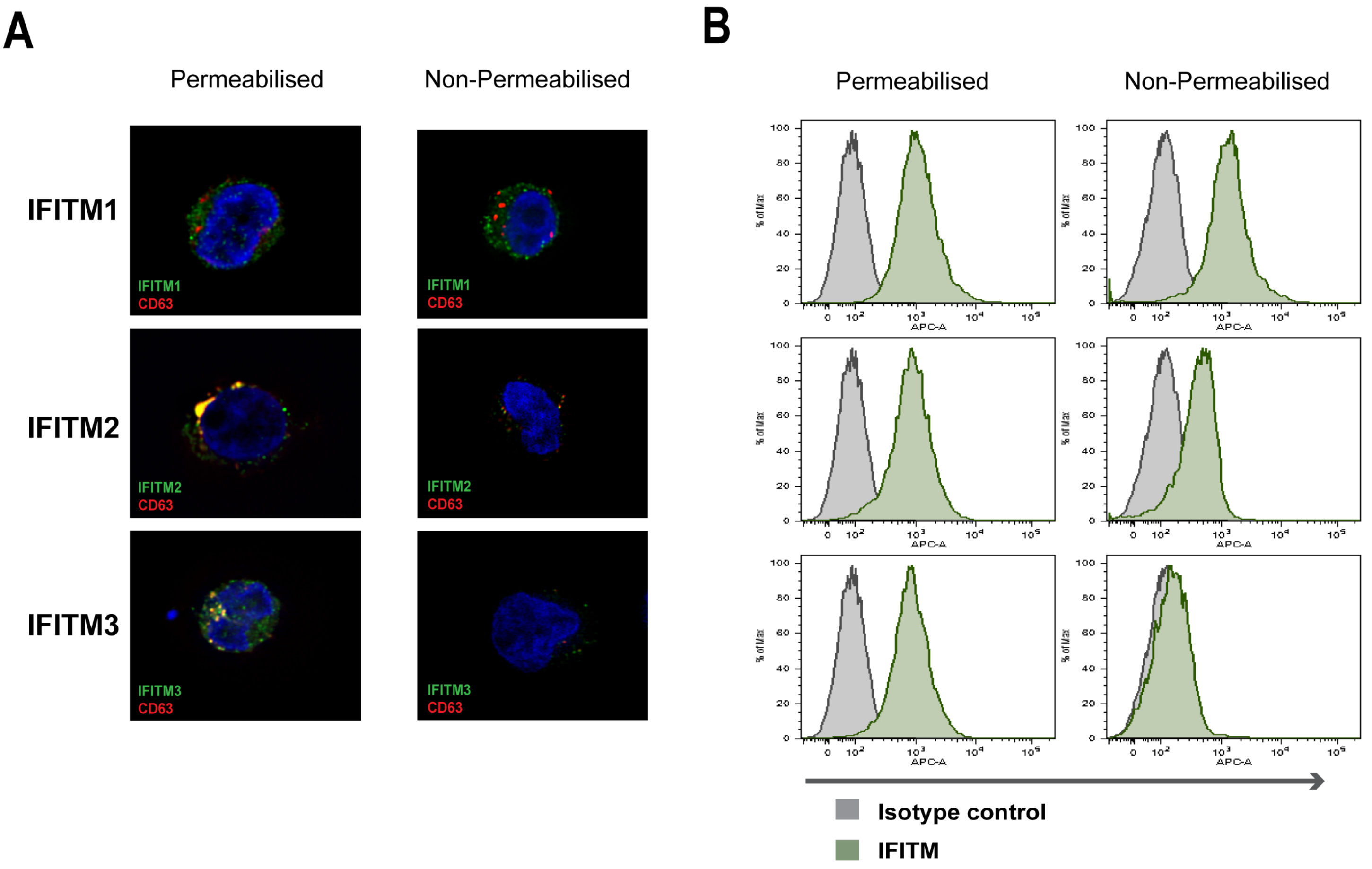

Figure S5, related to Figure 6. Cellular localisation of IFITM proteins in transduced Jurkat T cell lines.

(A) Permeabilised and non-permeabilised Jurkat cells transduced with the HA-tagged IFITMs 1, 2 or 3 proteins. Cells were stained with rabbit anti-HA (green) antibody followed by Alexa 488 conjugated anti-Rabbit IgG. Samples were co-stained with anti-CD63 (red) antibody followed by incubation with Alexa 594-conjugated IgG antibody to assess localization to late endosomes. Panels are of representative examples and images were deconvolved using the AutoQuant X3 software.

(B) Representative data of IFITM protein expression (green) in permeabilised or non-permeabilised Jurkat cell lines stably expressing the HA-tagged IFITMs 1, 2 or 3 proteins as assessed by flow cytometry. Isotype control is in light grey.

**CH058**	T232A **(V3)** * N338D **(C3)** * R579S **(NHR)** * A830T **(E)**
**CH077**	R4K T189N **(V1/V2)** K189E **(V1/V2)** * D229E **(C2)** * D345N **(C2)** R352K **(C3)** * E823G **(E)**
**CH236**	M167V **(V1/V2)** T308A **(V3)** * G509E **(C5)** G516E **(FP)** D676N **(TM)** R800K **(E)** V838I **(E)**
**CH470**	V275I **(C2)** * V588M **(NHR)** A859T **(E)**
**CH850**	I18V Y219H **(C2)** * D816N **(E)**

Table S1, related to Figures 3 and S3C. **Amino acid changes between T/F virus compared with its matched 6 month consensus clone**. Amino acid positions located in gp120 variable and constant regions are indicated with (V) and (C), respectively. Amino acid changes in the fusogenic peptide (FP), N-terminal heptad repeat (NHR), transmembrane domain (TM) or endodomain of gp41 are indicated.
* highlights amino acid positions, shown in Figure S3C.
See also Figure 3.

Table S2, related to Figure 4: Neutralisation escape mutations in Env of 6mo consensus sequences

**CH040 Nab**	G145E<br>E146 K/G<br>K160N<br>T295N<br>N300H<br>R327K<br>E332K
**CH077 Nab**	N136D<br>K186A<br>T187a (A/N)<br>N187b (S/D)<br>T188I<br>D230E
**CH058 Nab**	T232A<br>E336K<br>N339 (H/T/S/D)<br>K343Q/R<br>G410E<br>del 409-411

Supplemental Experimental Procedures

General cell culture

HEK293T cells were obtained from ATCC (American Tissue Culture Collection) and the HeLa-TZMbl reporter cell line, was kindly provided by J. Kappes through the NIH AIDS Reagents Repository Program (ARRP). Human glioblastoma U87-MG cells modified to express HIV-1 receptors CD4, CXCR4 or CCR5 were provided by M. Malim (Goujon et al., 2013)Cells were selected with 1 μg/ml puromycin and 100 μg/ml G418. 293T, HeLa-TZMbl and U87 cell lines were maintained in Dulbecco's modified Eagle's medium (DMEM), 10% fetal calf serum and 20μg/ml Gentamycin (Invitrogen, UK).

Isolation and culture of Primary cells

Human primary $CD4^+$ T cells were isolated from peripheral blood mononuclear cells (PBMCs) of healthy human donors. $CD4^+$ T cells were isolated by density gradient centrifugation through Lymphoprep (Axis-Shield) and obtained by negative selection using the Dynabeads Untouched Human CD4 T Cells kit (Life Technologies) according to the manufacturer's instructions. The purity of the isolated cell population was assessed by flow cytometry for CD4 and was reproducibly found to be > 95%. Cells were cultured in RPMI supplemented with 10% FCS, 30 U/ml recombinant IL-2 (Roche) and 20μg/ml gentamycin at 37°C in 5% CO_2 and activated within 48hrs using Dynabeads Human T-Activator CD3/CD28 beads (CD3/CD28 Dynabeads; Invitrogen) according to the manufacturer's instructions. Prior to infection or post-activation analysis, CD3/CD28 Dynabeads were removed using a DynaMag-2 magnet (Invitrogen), cells washed and resuspended in fresh RPMI-IL-2 supplemented media. When indicated, IFN-α (universal type 1 IFN, PBL InterferonSource) stimulation was performed using 500 U/ml for 24 h before immunoblotting or virus infection.

Ethics Statement

Ethical approval was granted by King's College London Infectious Disease BioBank Local Research Ethics Committee (under the authority of the Southampton and South West Hampshire Research Ethics Committee—approval REC09/H0504/39), approval number SN-1/6/7/9.

IFITM plasmid constructs

Human IFITM1, IFITM2 and IFITM3 were cloned into pLHCX retroviral vector (Clontech). Mutants IFITM2-Y19F and IFITM3-Y20F were generated by site-directed mutagenesis using the parental pLHCX IFITM1, 2 or 3 constructs as templates.

IFITM1, IFITM2, IFITM3 and Y19F or Y20F mutants thereof were all HA-tagged by PCR- based mutagenesis again using the parental pLHCX-IFITM1, 2 or 3 as templates.

HIV-1 infectious molecular clones

The proviral HIV-1 NL4.3, YU2, 89.6, JR-FL, JR-CSF, THRO, WITO, RHPA, and CH105 constructs were obtained from the National Institutes of Health (NIH) AIDS Reagent program.

Virus isolates 2024 and 2028 were kindly provided by P.Clapham.

The infectious transmitted/founder (T/F) HIV-1 molecular clones, their corresponding matched 6 month chronic pairs (6mo) and 6 month chronic neutralisation escape reversion clones (6mo Nab), of CH040, CH058, CH077, CH236, CH470 and CH850 were kindly provided by B.Hahn (co-author).

HIV-1 Env plasmids.

The pSVIII plasmids encoding the Env protein of interest of JR-FL, JR-CSF, CAP45.2.00.G3, 3016.v5.c45, Q461.e2, REJO, RHPA, TRJO, ZM53M.PB12, ZM197M.PB7, 191955_A11, 191084_B7-19, CH040, WITO were kindly provided by Katie Doores (co-author). The JR-FL G366E mutant was generated by site-directed mutagenesis using the parental pSVIII JR-FL Env plasmid as the template.

The following CAP256 envelopes in a pSVIII backbone were provided by P. Moore (Centre for the AIDS Programme of Research in South Africa) [Doria-Rose *et. al.,* 2014]: CAP256.1MO.C7J (1mo), CAP256.3MO.C9 (3mo), CAP256.8MO.31 (8mo), CAP256.12MO.1 (12mo), CAP256.14MO.5b (14mo), CAP256.21MO.A1 (21mo) and CAP256.39MO.10 (39mo).

The pCRV1 plasmids encoding the Env proteins of NL4.3, 89.6, YU2, CH058 T/F, CH058 6mo, CH077 T/F, CH077 6mo, CH470 T/F and CH470 6mo were generated by PCR amplification and insertion via EcoRI and NotI restriction sites.

pSVIII plasmids containing the chimeric envelopes of YU2 and Hxb2 with V3 loop substitution, i.e. YU2/Hxb2 V3 loop and Hxb2/YU2 V3 loop were kindly provided by J. Sodroski [ref].

HIV-1 molecular clone virus production

293T cells plated on 10cm dishes were transfected, using 1 mg/ml polyethyleneimine (Polysciences), typically with 10μg of the HIV-1 molecular clones. Media was changed 8-16 hours post transfection and viral supernatants harvested and filtered 48 hours post-transfection. For concentrated stocks, supernatants were incubated at 37°C for 2 hrs in the presence of 10 μM $MgCl_2$ and 20 U/ml DNAse (Roche) before concentration through a 20% sucrose cushion. Infectious end-point viral titers were determined on HeLa-TZMbl cells as previously described (Le Tortorec and Neil, 2009).

Production of HIV-1 env pseudotyped viral vectors

Generation of recombinant env-pseudotyped HIV-1 vectors, encoding GFP, was performed by co-transfection of 293T cells with pCRV1 HIV-1 gag-pol, pCSGW (packaging vector containing the eGFP reporter gene) and the pCVRV1 or pSVIII plasmid, as indicated, encoding the Env protein of interest. The ratio of the HIV-1 gag-pol plasmid DNA to pCSGW to pCRV1/pSVIII env plasmid DNA was optimised at a 2:3:1 ratio. Viral supernatants were harvested 48 hrs post transfection, filtered and titred on the appropriate U87-MG cell line by flow cytometry.

For neutralisation assays, pseudotyped virus was produced in 293T cells by co-transfecting the plasmids encoding Env with pSG3ΔEnv (an Env-deficient genomic backbone plasmid) in a 1:2 ratio using polyethylenimine (PEI) at 1 mg/ml. 48 hrs post transfection, viral supernatants were harvested and filtered. Viral titre was determined by titration of pseudotyped virus stocks onto HeLa-TZMbl cells and culturing for 48 hrs prior to calculating the $TC1D_{50}$ values according to the Reed and Meunch equation.

Generation of U87-MG IFITM stable cell lines

U87-MG CD4-CXCR4 and U87-MG CD4-CCR5 cell lines stably expressing the IFITMS 1, 2, 3, IFITM2-Y19F or IFITM3-Y20F proteins, with and without HA-tags, were generated by retroviral transduction. Cells were selected with 100 μg/ml hygromycin.

Expression of IFITM proteins was assessed by western blot. Cell lysates were subjected to SDS-PAGE, and Western blot assays were performed using rabbit-anti HSP90 (Santa Cruz), mouse anti-IFITM1 and rabbit anti-IFITM2 or 3 (ProteinTech) antibodies. When indicated, IFN-α (universal type 1 IFN, PBL InterferonSource) stimulation was performed using 1000 U ml^{-1} for 24 h before immunoblotting or virus infection.

Flow cytometry

Flow cytometry analyses were performed using a BD FACSCanto II flow-cytometer (Becton Dickinson) and FlowJo software. Env-pseudotyped HIV-1 viral vector infected cells were analysed for expression of GFP. CD4, CXCR4 and CCR5 surface expression in the presence of IFITM 1, 2 or 3 were determined by staining with APC conjugated mouse anti-human CD4 (BD Pharmingen), PE conjugated mouse CXCR4 (CD184) (BD Pharmingen) and PE conjugated mouse CCR5 (CD195) (BD Pharmingen), respectively.

Viral replication assays

U87 $CD4^+$ $CXCR4^+$ or $CD4^+$ $CCR5^+$ cells stably expressing IFITMs 1, 2 or 3 or mutants thereof were infected with the indicated HIV-1 molecular clone at a multiplicity of infection (MOI) of 0.05. Media was replaced 8 hrs post infection and culture supernatants were harvested and stored every 24 hours post infection for a total of 120 hrs. Infectious viral release at each time point was determined by infecting HeLa-TZMbl indicator cells and 48 hrs post infection assaying for virus release by measuring chemiluminescent β-galactosidase activity using the Tropix Galacto-Star system (Applied Biosystems) according to the manufacturer's instructions. For one-round virus release assays, cells were infected with the indicated HIV-1 molecular clone at an MOI of 0.5. Viral production was measured for supernatants harvested at 48 hrs post infection on HeLa-TZMbl indicator cells, as above.

For analysis of envelope content of physical virus particles, supernatants were filtered through a 0.22 μm filter (Merck Millipore) and pelleted through a 20% sucrose/PBS cushion at 28 000 rpm for 90 min at 4 °C. Virion and cell lysates were subjected to SDS-PAGE and Western blotted for HIV-1 p24CA using monoclonal antibody 183-H12-5C; kindly provided by B. Chesebro through the NIH AIDS Reagent program, and for HIV-1 gp160 and gp120 using antiserum to HIV-1 gp120 (ARP421) obtained from the Centre of AIDS reagents.

siRNA mediated AP2 knockdown

siRNA mediated knock down of AP2 in U87-MG $CD4^+$ $CXCR4^+$ or $CD4^+$ $CCR5^+$ control cells or cells expressing IFITMS 1, 2, 3, IFITM2-Y19F or IFITM3-Y20F was performed as previously described (Kueck and Neil, 2012). SMARTpool siRNA was purchased from Dharmacon (#L-008170-00-0005) and represents a pool of four different siRNAs
(1, GAACCGAAGCUGAACUACA; 2, AGUUUGAGCUUAUGAGGUA;
3, GCGAGAGGGUAUCAAGUAU; 4, GUUAAGCGGUCCAACAUUU) (Thermo Scientific). Non-targeting siRNA was used as a control (Thermo Scientific). Viral supernatants were harvested at 48 hrs and infectivity was determined by infecting HeLa-TZMbl indicator cells. Cell lysates were subjected to SDS-PAGE, and Western blot assays were performed using a rabbit-anti HSP90 (Santa Cruz), and mouse monoclonal anti-AP50 antibody (BD Biosciences).

Endocytosis Inhibitor studies

Dynamin inhibitor, Dynasore monohydrate (Sigma), and Clathrin inhibitor Pitstop2 (Abcam) were dissolved in dimethyl sulphosxide (DMSO). Cells were treated with 80 μM Dynasore, 30 μM Pitstop2 or DMSO alone for 30 minutes at 37 °C prior to infection with env-pseudotyped HIV-1 viral vectors for 48 hours. Following incubation, cells were analysed for viral vector entry by flow cytometry. AlexaFluor 488 conjugated Transferrin (Thermo Fisher Scientific) was used as a control for the inhibition studies. 50 μg/ml transferrin was added to Dynasore or Pitstop2 treated cells on coverslips for 10 minutes at room temperature. Cells were washed 3 x in the presence or absence of the endocytosis inhibitors before incubation at 37 °C for 30 minutes. Following incubations, cells were fixed in 4% paraformaldehyde/PBS and washed with 10 mM glycine/ PBS.

Cells were mounted on glass slides using ProLong AntiFade- 4',6-diamidino-2-phenylindole (DAPI) mounting solution (Molecular Probes, Invitrogen) and images were captured with a Nikon ESCLIPSE Ti inverted microscope. Z stacks were

taken of all cells, images deconvoluted using AutoQuant X3 and analyzed using the ImageJ software. Representative images are shown.

Lentiviral mediated shRNA knockdown of IFITM expression

Silencing of IFITM expression was mediated by lentiviral shRNA knockdown in primary human CD4+ T cells. IFITM1, IFITM2 and IFITM3 specific hairpins were cloned into pHRSIREN-PGK-GFP (Matheson et al., 2014) encoding a U6-shRNA cassette and GFP for flow cytometric analysis. The targeting (sense) sequences used were the following:

shIFITM1: GGTCCACCGTGATCAACAT

shIFITM2: GCCACGTACTCTATCTTCCAT

shIFITM3: GCCCACGTACTCCAACTTCCT

Control: shLacZ control: GTTATAGGCTCGCAAAAGG

VSV-g pseudotyped pHRSIREN lentiviral vectors were produced by co-transfection of 293T cells with pCRV1 HIV-1 *gag-pol*, pHRSIREN-PGK-GFP and pMD.G VSV-g (at a ratio of 2:3:1), and primary human CD4+ T cells were transduced with at an input equivalent to a MOI of 5, when titered on HeLa-TZMbl cells, and cultured for 48-72h before assay use. Transduction efficiency was assessed by flow cytometry (typically between 80 and 98% efficiency of transduction and silencing efficiency was assessed by Western blot. Cell lysates were subjected to SDS-PAGE, and Western blot assays were performed using rabbit-anti HSP90 (Santa Cruz), mouse anti-IFITM1 and rabbit anti-IFITM2 or 3 (ProteinTech) antibodies. When indicated, IFN-α (universal type 1 IFN, PBL InterferonSource) stimulation was performed using 500 U ml^{-1} for 24 h before immunoblotting or virus infection.

Primary cell infections

2×10^5 activated $CD4^+$ T cells, transduced with the appropriate shRNA lentiviral vectors, were infected at an MOI of 0.1; 8 to 12 hours post infection, media was replaced. Supernatants were harvested every 72, 120 and 168 hrs post infection and virus particle production was assessed on HeLa-TZMbl cells as described previously.

CRISPR knockout of IFITM expression in U87-MG cells

CRISPR oligos were designed to target IFITM1 (TGATCACGGTGGACCTTGGA), IFITM2 (CATCTCGTAGTTGGGAGGC), IFITM3 (GCTCAAGGAGGAGCACGAGG) and Luciferase (CCTCTAGAGGATGGAACCGC). Oligos were cloned into an adapted version of the LentiCRISPR v.2 plasmid (Addgene), where the puromycin gene was replaced with the green florescent protein (GFP) gene, according to the manufacturer's instructions. Viral stocks were then produced in 293T cells. Briefly, cells were transfected with pCRVI HIV gag-pol, pMD2.G VSV-g and the LentiCRISPR v.2 GFP of interest at a ratio of 2.5: 5: 2.5 using 1 mg/ml polyethyleneimine. Media was changed 16 hrs post transfection and supernatant was harvested and filtered 48 hrs post transfection. U87-$CD4^+$ $CCR5^+$ cells transduced with the LentiCRISPR virus at 1,600 rpm for 1 hr. Cells were cultured for a further 7 days allowing for efficient knockout of the target protein. CRISPR knockout efficiency was then determined by western blot, where cell lysates were subjected to SDS-PAGE, and assays performed using rabbit-anti HSP90 (Santa Cruz), mouse anti-IFITM1 and rabbit anti-IFITM2 or 3 (ProteinTech) antibodies. When indicated, IFN-α (universal type 1 IFN, PBL InterferonSource) stimulation was performed using 1000 U ml^{-1} for 24 h before immunoblotting to determine level of IFITM knockout. The effect of the CRISPR knockouts on virus replication was assessed by a time course assay where supernatant was harvested every 72, 120 and 168 hrs.

Immunofluorescence

2 x 10^5 U87-MG $CD4^+$ $CXCR4^+$ or $CD4^+$ $CCR5^+$ cells stably expressing HA-tagged IFITM 1, 2 or 3 proteins or mutants thereof were grown on coverslips precoated with 0.1% (w/v) poly-L-lysine (Sigma). Cells were fixed in 4% paraformaldehyde/PBS, washed with 10 mM glycine/ PBS, and permeabilized in 1% bovine serum albumin/ 0.1% Triton-X-100/ PBS for 15 min. Cells were stained using anti-rabbit polyclonal HA antibody (Rockland) in combination with sheep anti-human CD46 or EEA1 (AbD Serotec), followed by the appropriate secondary antibodies conjugated to Alexa 488 or 594 fluorophores (Molecular Probes, Invitrogen). Cells were mounted on glass slides using ProLong AntiFade- 4',6-diamidino-2-phenylindole (DAPI) mounting solution (Molecular Probes, Invitrogen)

and images were captured with a Nikon ESCLIPSE Ti inverted microscope. Z stacks were taken of all cells, images deconvoluted using AutoQuant X3 and analyzed using the ImageJ software. Representative images are shown.

CD45-depletion and IFITM virion incorporation assays

U87-MG $CD4^+$ $CXCR4^+$ or $CD4^+$ $CCR5^+$ Control cells or cells expressing IFITMS 1, 2, 3, IFITM2-Y19F or IFITM3-Y20F were plated onto 10 cm dishes at a density of 2 x 10^6 were infected with HIV-1 molecular clone 89.6 at an MOI of 3. 72 hrs post-infection, supernatants were harvested, filtered and pelleted through a 20% sucrose/PBS cushion at 28 000 rpm for 90 min at 4 °C. Virus pellets were resuspended in PBS and prior to CD45 depletion, viral particles were normalised to HIV-1 p24-Gag ELISA (Perkin Elmer) according to manufacturer's instructions.

Matched p24 quantities were incubated with anti-CD45 antibody coupled magnetic beads (Miltenyi) for 2 hrs. Beads were recovered on a DynaMag-2 magnet (Invitrogen) following manufacturer's instructions. CD45-depleted and non-depleted fractions were then precipitated with a final concentration of 10% TCA and immunoblotted using mouse anti-IFITM1 and rabbit anti-IFITM2 or 3 (ProteinTech) antibodies, using monoclonal antibody 183-H12-5C for HIV-1 p24CA; kindly provided by B. Chesebro through the NIH AIDS Reagent program and mouse anti- human CD45 antibody (Biolegend) for determining the degree of CD45 depletion.

CD4 competition assays

U87-MG $CD4^+$ $CCR5^+$ were infected at an MOI of 0.5 with the indicated HIV-1 molecular clone/anti-human CD4 (SK3 clone-Biolegend) antibody mix. Anti-human CD4 antibody was used at concentrations of 100, 10, 0 ng/ml. Cells were carefully washed and media was replaced 6 hours post infection. 48 hrs post infection, supernatants were harvested and used to infect HeLa-TZMbl cells, assaying for any dose-dependent reduction in virus release by measuring chemiluminescent β-galactosidase activity using the Tropix Galacto-Star system (Applied Biosystems) according to the manufacturer's instructions.

Statistical analysis

Datasets were assessed for the significance of inhibitory differences between X4 and R5 using viruses (Figure 1C) using unpaired two-tailed T tests. Datasets in Figure 3B were assessed for statistical significance between T/F and match 6mo chronic clones using paired two-tailed Mann-Whitney tests. All statistical analyses were carried out using GraphPad Prism v5.0.b. Levels of significance were determined as follows: *** $p<0.001$, ** $p<0.01$, *$p<0.05$, ns $p>0.05$.

Supplemental References

Goujon, C., Schaller, T., Galao, R.P., Amie, S.M., Kim, B., Olivieri, K., Neil, S.J., and Malim, M.H. (2013). Evidence for IFNalpha-induced, SAMHD1-independent inhibitors of early HIV-1 infection. Retrovirology *10*, 23.

Kueck, T., and Neil, S.J. (2012). A cytoplasmic tail determinant in HIV-1 Vpu mediates targeting of tetherin for endosomal degradation and counteracts interferon-induced restriction. PLoS Pathog *8*, e1002609.

Le Tortorec, A., and Neil, S.J. (2009). Antagonism to and intracellular sequestration of human tetherin by the human immunodeficiency virus type 2 envelope glycoprotein. J Virol *83*, 11966-11978.
