# Supplementary material for: Resistance of Transmitted Founder HIV-1 to IFITM-Mediated Restriction
Source: Cell Host Microbe. 2016 Oct 12;20(4):429–42. doi: 10.1016/j.chom.2016.08.006 (PMC5075283; doi:10.1016/j.chom.2016.08.006)
Supplement: Document S2. Article plus Supplemental Information [file mmc2.pdf]

Article

Cell Host & Microbe

Resistance of Transmitted Founder HIV-1 to IFITM-Mediated Restriction

Graphical Abstract

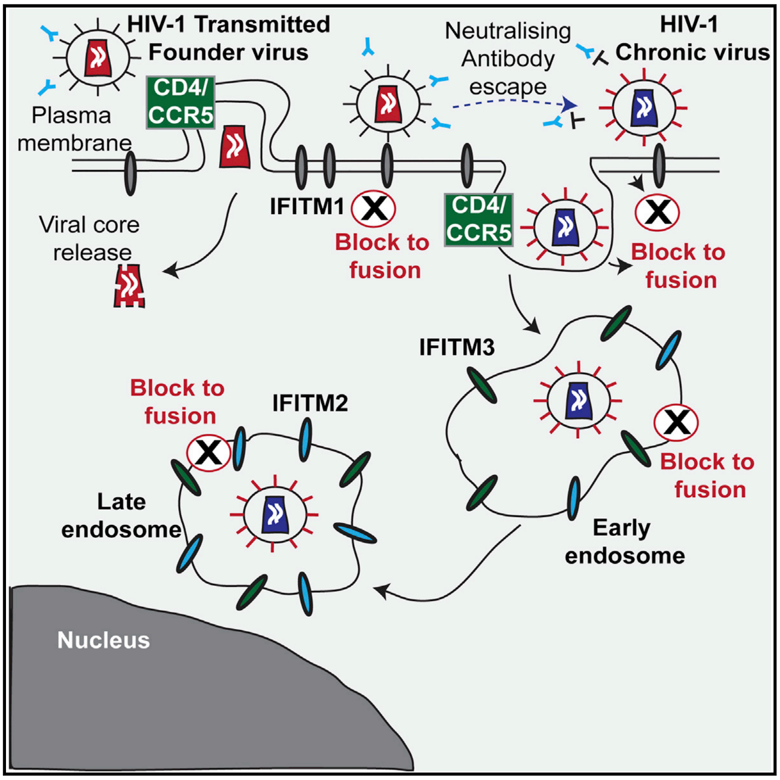

Highlights

- IFITM restriction of HIV-1 depends on virus co-receptor use and IFITM localization
- Transmitted founder viruses are resistant to IFITM restriction
- Escape from early neutralizing antibodies confers IFITM sensitivity
- IFITM restriction contributes to the increased IFN sensitivity of chronic viruses

Authors

Toshana L. Foster, Harry Wilson, Shilpa S. Iyer, ..., Persephone Borrow, Beatrice H. Hahn, Stuart J.D. Neil

Correspondence

stuart.neil@kcl.ac.uk

In Brief

IFITMs are interferon-induced proteins with broad antiviral activity. Foster et al. demonstrate that HIV-1 sensitivity to IFITMs depends on the viral entry route into target cells. Strikingly, transmitted HIV-1 strains are IFITM resistant, but viral envelope mutations that escape neutralizing antibody responses after infection lead to IFITM and interferon sensitivity.

Foster et al., 2016, Cell Host & Microbe *20*, 429–442
October 12, 2016 © 2016 The Author(s). Published by Elsevier Inc.
http://dx.doi.org/10.1016/j.chom.2016.08.006

Cell Host & Microbe
Article

Resistance of Transmitted Founder HIV-1 to IFITM-Mediated Restriction

Toshana L. Foster,[1] Harry Wilson,[1] Shilpa S. Iyer,[2] Karen Coss,[1] Katie Doores,[1] Sarah Smith,[3] Paul Kellam,[3] Andrés Finzi,[4] Persephone Borrow,[5] Beatrice H. Hahn,[2] and Stuart J.D. Neil[1,6,*]
[1]Department of Infectious Diseases, King's College London Faculty of Life Sciences and Medicine, Guy's Hospital, London SE1 9RT, UK
[2]Departments of Medicine and Microbiology, Perelman School of Medicine, University of Pennsylvania, Philadelphia, PA 19104, USA
[3]Wellcome Trust Sanger Centre, Hinxton, Cambridge CB10 1SA, UK
[4]Centre de Recherche du CHUM and Department of Microbiology, Infection, and Immunology, Université de Montréal, Montréal, QC H3T 1J4, Canada
[5]Nuffield Department of Medicine, University of Oxford, Oxford OX1 2JD, UK
[6]Lead Contact
*Correspondence: stuart.neil@kcl.ac.uk
http://dx.doi.org/10.1016/j.chom.2016.08.006

SUMMARY

Interferon-induced transmembrane proteins (IFITMs) restrict the entry of diverse enveloped viruses through incompletely understood mechanisms. While IFITMs are reported to inhibit HIV-1, their in vivo relevance is unclear. We show that IFITM sensitivity of HIV-1 strains is determined by the co-receptor usage of the viral envelope glycoproteins as well as IFITM subcellular localization within the target cell. Importantly, we find that transmitted founder HIV-1, which establishes de novo infections, is uniquely resistant to the antiviral activity of IFITMs. However, viral sensitivity to IFITMs, particularly IFITM2 and IFITM3, increases over the first 6 months of infection, primarily as a result of neutralizing antibody escape mutations. Additionally, the ability to evade IFITM restriction contributes to the different interferon sensitivities of transmitted founder and chronic viruses. Together, these data indicate that IFITMs constitute an important barrier to HIV-1 transmission and that escape from adaptive immune responses exposes the virus to antiviral restriction.

INTRODUCTION

Robust systemic type 1 interferon (IFN-1) responses are among the earliest host innate immune defenses during acute SIV and HIV-1 infection (Abel et al., 2005; Stacey et al., 2009). In primary CD4$^+$ T cells and macrophages, expression of IFN-induced genes (ISGs) restricts viral replication (Goujon and Malim, 2010), and treatment of rhesus macaques with IFN-1 increased the number of intrarectal challenges required to achieve systemic SIVmac infection and reduced the number of transmitted founder (TF) viruses (Sandler et al., 2014). While the virus encodes countermeasures against important ISGs, such as APOBEC3G and tetherin (BST2/CD317), other ISGs appear to restrict viral replication in cell culture with no obvious viral evasion mechanism, and thus their physiological relevance in the transmission and pathogenesis of HIV/AIDS remains unclear (Doyle et al., 2015).

One such family of ISGs, the IFN-induced transmembrane proteins 1–3 (IFITMs 1–3), has broad activity against diverse enveloped viruses, particularly influenza A virus (IAV) (reviewed in Smith et al., 2014). IFITMs are small (125–135 amino acids) membrane-spanning proteins whose topology is still a matter of debate. In the most favored conformation (Bailey et al., 2013; Ling et al., 2016; Weston et al., 2014), the N-terminal region is cytosolic, followed by a semi-transmembrane (TM) domain that remerges from the cytosolic face, which, after an intracellular loop containing essential palmitoylation sites (Yount et al., 2010), turns into a canonical TM helix that exposes the C terminus on the extracellular side. In humans IFITMs 2 and 3 are highly homologous with only ten amino acid differences between them. Both have longer N-terminal tails than IFITM1, in which an overlapping PPXY and YxxΦ site interacts with NEDD4 family ubiquitin ligases (Chesarino et al., 2015) and the clathrin adaptor AP-2, respectively (Jia et al., 2014). IFITM2 and 3 localize predominantly to different endosomal compartments at steady state (Weston et al., 2014). This is determined in part by the AP-2 binding, implying that they traffic via the cell surface (Jia et al., 2012, 2014; Weston et al., 2014). By contrast, IFITM1 lacks an obvious trafficking sequence and is primarily expressed at the plasma membrane. A human polymorphism defined by a SNP, rs12252-C, has been proposed to lead to an alternatively spliced variant of IFITM3 that truncates the N terminus after the YxxΦ motif, thereby reducing its antiviral activity against IAV and accounting for enhanced morbidity in the recent H1N1 swine flu pandemic (Everitt et al., 2012).

IFITM3 has been shown to restrict IAV entry at the stage of fusion in the endosome (Amini-Bavil-Olyaee et al., 2013; Desai et al., 2014; Li et al., 2013). Although the mechanism is not well understood, this restriction may be due to the effects of IFITM3 on membrane fluidity and/or cholesterol trafficking or biosynthesis (Amini-Bavil-Olyaee et al., 2013; Desai et al., 2014; Lin et al., 2013). In contrast to the effects of IFITMs on pH-dependent virus entry, their ability to restrict HIV-1 is less clear cut. The antiviral effects of IFITMs so far observed have been variously ascribed to virion incorporation during assembly (Compton

This is an open access article under the CC BY license (http://creativecommons.org/licenses/by/4.0/).

et al., 2014; Tartour et al., 2014) or inhibition of processing of the HIV-1 envelope glycoprotein (Yu et al., 2015), with little consensus as to their mechanism and sites of action. It is, however, difficult to rationalize these mechanisms with the clear inhibitory effects in the target cell for all other enveloped viruses thus far examined. Moreover, the effect that IFITMs might have on HIV-1 tropism has not been studied.

HIV-1 enters cells through engagement of its envelope glycoprotein with CD4 and a chemokine receptor co-receptor, CCR5 (R5) or CXCR4 (X4) (Wilen et al., 2012). CD4 interaction induces conformational changes in the surface (SU) subunit of Env, gp120, which exposes the co-receptor-binding site. Upon co-receptor engagement, further conformational changes in the TM gp41 subunit activate its membrane fusion capacity. R5 usage is essential for viral transmission, with a switch to X4 observed in ~50% of patients with subtype B infections but less common in other clades. Furthermore, while R5 and X4 viruses are both capable of infecting $CD4^{+}$ T cells, tropism of R5 strains for macrophages is more complex. Most R5 viruses, including TF viruses, cannot efficiently infect macrophages. Macrophage tropism is almost exclusively R5 dependent, but also requires adaptation in gp120 to use very low levels of CD4 (Duenas-Decamp et al., 2010). It is also not clear why, despite constitutive expression of CXCR4 on macrophages, most X4 viruses cannot infect them (Simmons et al., 1998). Finally, there are examples of HIV-1 and SIV restriction in certain cell types that appear dependent on the route of viral entry (Pineda et al., 2007; Schmitz et al., 2004). Although generally thought of as a virus that fuses at the cell surface, some HIV-1 strains fuse in endosomes in some cell types (Miyauchi et al., 2009).

Given these observations, we set out to examine whether IFITMs could restrict HIV-1 strains with differing receptor tropisms. In doing so, we found that IFITM restriction of HIV-1 is modulated by co-receptor usage and subcellular localization of the IFITM, suggesting different entry pathways depending on Env/receptor interactions. Furthermore, we found that TF viruses are uniquely IFITM resistant, a property that is lost during chronic infection, in part due to escape mutations acquired in response to autologous neutralizing responses.

RESULTS

IFITMs Differentially Restrict HIV-1 Isolates Depending on Co-receptor Tropism

To examine the role of IFITMs in restricting HIV-1 replication, we constructed U87 neuroblastoma cells (which do not express detectable IFITMs without IFN induction) encoding CD4 together with one of the two major co-receptors, CXCR4 or CCR5, to express IFITM1, IFITM2, and IFITM3, respectively (Figure S1A, available online). Importantly, the IFITM expression levels in these engineered cell lines were of a similar magnitude compared to those of monocyte-derived macrophages and $CD4^{+}$ T cells in the presence and absence of IFN-1 (Figure S1B). IFITM expression did not alter CD4 or co-receptor expression in these cells (Figure S1C). We then analyzed the infectivity of HIV-1 Env pseudotypes as well as replication-competent molecular clones in these cells (Figures 1A, 1B, S1D, and S1E). Testing both single-round infectivity and cumulative 96-hr replication, we found that X4-using viruses displayed a significantly greater sensitivity to IFITMs 2 and 3 than did the R5 viruses (Figures 1, S1D, and S1E). In contrast, most R5 viruses tested were more sensitive to IFITM1 than X4 viruses. However, this difference achieved statistical significance only after the removal of two outlier R5 strains (CH105 and THRO), the reasons for which will be addressed below. Intriguingly the R5/X4 isolate 89.6 displayed distinct restriction patterns dependent on whether it entered target cells using CXCR4 or CCR5 (Figures 1A, 1B, and S1D). In the former case it had a singular sensitivity to IFITM2, whereas in the latter case this was relieved in favor of greater IFITM1 sensitivity.

To formally demonstrate that co-receptor usage influences IFITM sensitivity, we pseudotyped lentiviral vectors with envelopes from prototypic R5 (YU2) and X4 (HxB2) using strains in which the determinant of co-receptor use, the V3 loop (Sullivan et al., 1998), had been exchanged. This resulted in exchange of the restriction phenotype between the two envelope proteins (Figure 1C). This indicates that a given IFITM's antiviral activity is modulated by the receptor requirements of the infecting virus. Importantly one-round infectivities and multiple-round replication results correlated, and we saw no differential effects of IFITM expression on envelope precursor processing to gp120 (Figure S1F), suggesting IFITM restriction only affected Env-mediated cell entry, in contrast to a previous report (Yu et al., 2015).

Subcellular Localization of IFITMs Correlates with Differential HIV-1 Restriction

The differential restriction of X4 and R5 tropic HIV-1 prompted us to examine whether IFITM localization underlies these phenotypes. Available antibodies cannot distinguish between human IFITMs by immunofluorescence, so we used C-terminally HA-tagged proteins stably expressed in U87 cells. Unlike IFITM1, which is predominantly found at the cell surface, IFITMs 2 and 3 localize to endosomal compartments (Figure 2A) (Weston et al., 2014). Endosomal localization of IFITM2 and 3 depends on a YXXΦ-binding site for the clathrin adaptor AP-2. When the Y residue is mutated to a phenylalanine, both proteins readily localize to the cell surface (Figure 2B). Using the V3 loop-swap viral pseudotypes, we found that we could reverse the restriction patterns observed above: R5 tropism was sensitive to surface-expressed IFITM2 and 3, whereas X4 tropic restriction was relieved (Figure 2C). Similarly, the restriction of replication of the dual tropic 89.6 virus by IFITM2 in a CXCR4 context was completely abolished upon its relocalization to the cell surface (Figure 2D). We could mirror these findings by reversing 89.6's IFITM2 sensitivity by knocking down AP-2 in CXCR4-expressing cells (Figure 2E). We also could show that the V3 swap variants that use X4 could be rescued from IFITM2 and 3 restriction by the inhibition of endocytosis with the dynamin inhibitor dynasore and the clathrin inhibitor Pitstop2 (Figure 2F). The differential restriction patterns depending on IFITM localization suggest that X4 HIV-1 isolates may fuse preferentially in different subcellular compartments compared to R5 viruses.

Recent publications suggest that IFITM incorporation into HIV-1 particles affects their infectivity and contributes to viral restriction (Compton et al., 2014; Tartour et al., 2014). We examined incorporation of IFITMs and mutants into CD45-depleted pelleted 89.6 virions (Coren et al., 2008). Despite the

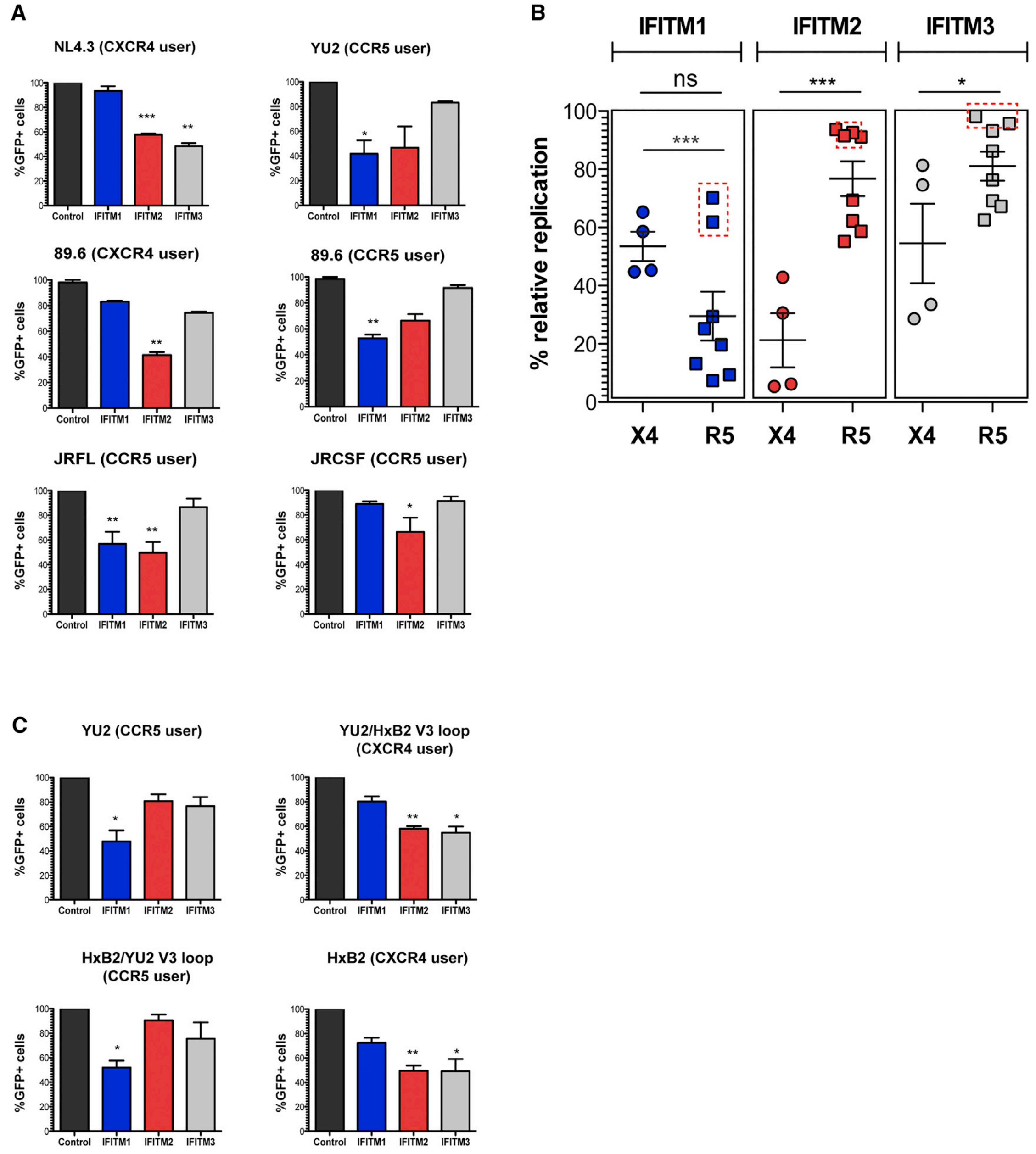

Figure 1. Sensitivity of HIV-1 to IFITM Inhibition Varies with Co-receptor Usage

(A) GFP-encoding HIV-1 vectors pseudotyped with the indicated envelope glycoproteins were used to infect U87/CD4/CCR5 or U87/CD4/CXCR4 cells stably expressing IFITMs 1, 2, or 3 or empty vector (control). The percentage of GFP$^+$ cells compared to control was determined by flow cytometry, and results shown represent a mean of independent experiments. Statistics were performed using an unpaired two-tailed t test (***$p < 0.001$; **$p < 0.01$; *$p < 0.05$; ns, $p > 0.05$).

(B) Cumulative viral replication at 96 hr in the presence of IFITMs differs for CXCR4-using or CCR5-using HIV-1 virus isolates. U87/CD4/CCR5 or U87/CD4/CXCR4 cells were infected at an MOI of 0.05, and replication was monitored by viral output on HeLa-TZMbl indicator cells (see also Figures S1D and S1E). Each virus point represents a mean of three independent experiments. Inset red dashed boxes: CCR5-using CH105 and RHPA are founder virus isolates that are resistant to IFITM inhibition. Removal of these outliers reveals data significance between CXCR4 and CCR5 users in the presence of IFITM1 (***$p < 0.001$; **$p < 0.01$; *$p < 0.05$; ns, $p > 0.05$, based on an unpaired two-tailed t test).

(C) Pseudotyped HIV vectors were produced with wild-type YU2 and Hxb2 envelopes or V3 loop swap variants and analyzed as in (A).

All error bars represent ± SEM (n = 3). See also Figure S1.

differential restriction of viral replication by endosomal IFITMs and mutants, we found no evidence that this correlated with virion incorporation, which remained constant (Figure S2B). Thus, restriction of HIV-1 entry depends on the localization of the IFITM in the target cell, and it suggests that viral sensitivity is the result of co-receptor-mediated targeting of the entry process to subcellular compartments where IFITMs are differentially expressed.

CellPress

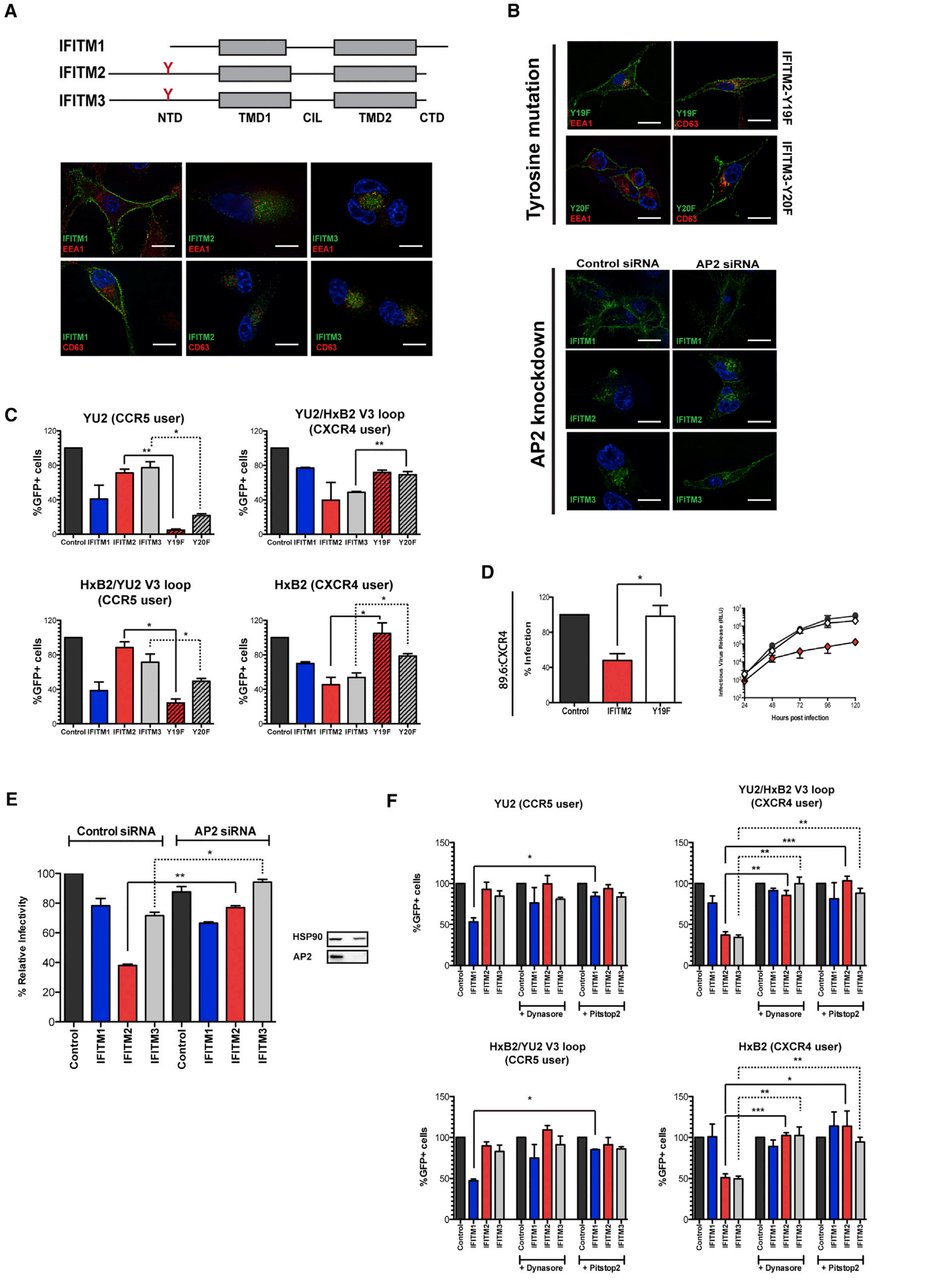

(legend on next page)

TF HIV-1 Strains Are Resistant to IFITMs

In the analysis shown in Figure 1, several viruses were almost completely resistant to IFITM restriction. In all cases these were TF viruses, which represent viruses that establish de novo infection following HIV-1 transmission (Salazar-Gonzalez et al., 2009). Using a panel of TF molecular clones (Figure 3A) as well as individual TF Envs (Figure S3A), we found that in U87CD4/CCR5 cells the great majority of these viruses were resistant to all three human IFITMs, with relocalization to the plasma membrane of IFITMs 2 and 3 having only minor effects (Figure S3B). For six TF viruses, matched 6-month consensus molecular clones from the same individual were available, all of which previously had been shown to be particularly sensitive to the antiviral effects of IFN-1 (Fenton-May et al., 2013). In all of these cases, we observed a striking increase in their sensitivities to IFITM2 and IFITM3 (Figures 3A and 3B), which was relieved upon IFITM2/3 relocalization to the plasma membrane (Figure S3B). All the TF/6-month pairs were singularly R5 tropic except for CH077, where the founder was able to use CXCR4 at a lower efficiency (not shown). The IFITM phenotypes could be transferred to lentiviral vectors pseudotyped with the respective envelope glycoproteins, indicating that amino acid changes in Env during the transition from acute to chronic infection were associated with IFITM-mediated restriction (Figure 3C). Furthermore, a panel of Envs derived from a clade C-infected patient, generated at the time of transmission through 39 months post-infection (Doria-Rose et al., 2014), exhibited progressive sensitization to IFITMs 2 and 3, with clear phenotypes appearing by 8 months (Figure 3D).

While there were no common amino acid changes in Env that were shared between the various TF/6-month pairs, most differences mapped to the external surfaces of the trimers and the variable loops (Figure S3C; Table S1). This raised the possibility that adaptive changes in Env that accrued in response to host immune responses during the course of the infection might explain the increased sensitivities to IFITMs 2 and 3. In three TF/6-month pairs, amino acid positions in the 6-month envelope previously have been identified to mediate evasion of early autologous neutralizing antibody (NAb) responses (Figure 4A; Table S2) (Bar et al., 2012). Importantly, experimental reversion of these amino acids in the 6-month molecular clones completely restored the IFITM resistance (Figure 4B).

The resistance of the 6-month viruses to surface-retained IFITM2 or 3 (Figure S3B) prompted us to seek evidence that alterations in viral entry modulated by receptor engagement could explain the increased IFITM sensitivities of the 6-month variants. First we found that the sensitivity of the 6-month viruses to IFITM2 or 3 could be abolished by AP-2 depletion (Figures 5A and S4A) or treatment of the cells with endocytosis inhibitors (Figures 5B and S4B). Since all the viruses were R5 tropic, we then compared TF/6-month pairs in the presence of limiting surface CD4 density with a blocking antibody. Intriguingly, for CH077, CH058, and CH470 TF clones and the corresponding NAb revertants, an IFITM2/3 restriction phenotype similar to the 6-month variant could be induced by limiting CD4 levels (Figure 5C). Conversely, the 6-month variants themselves maintained their restriction profile irrespective of entry inhibition by CD4 neutralization. Together these data argue that the primary engagement with CD4 at the plasma membrane is a major determinant of IFITM resistance in the TF viruses and the accrual of amino acid variations under immune pressure impacts on the cellular route of entry, leading to IFITM2 and 3 restriction.

IFITM Depletion in CD4$^+$ T Cells Rescues 6-Month Viral Isolates from IFN-1

A major challenge with ISGs that restrict HIV-1 in cultured cells is demonstrating that they have relevance in primary targets. The reported acquisition of IFN-1 sensitivity in the 6-month viruses (Fenton-May et al., 2013) prompted us to determine whether IFITMs were contributing factors. We first determined the localization of IFITMs in Jurkat T cells transduced with the same HA-tagged constructs used for microscopy in our U87 cells (Figures S5A and S5B). As in the U87 cells, IFITM1 was almost exclusively at the plasma membrane. Aside from some minor surface labeling, IFITM3 was found in intracellular endosomes. IFITM2 could be detected at the plasma membrane, but, upon permeablization, 3- to 4-fold more was stained in flow cytometry, indicating that the majority localization was again intracellular.

To assess the contribution of IFITMs to HIV-1 IFN-1 sensitivity, lentiviral vectors pseudotyped with TF or 6-month envs were used to transduce U87/CD4/CCR5 after pretreatment overnight with 500 U IFN-1. The 6-month envs displayed a greater reduction in one-round infectivity after IFN-1 treatment, indicating that

Figure 2. A Conserved N-Terminal Tyrosine Residue Is Crucial for IFITM Localization and Affects the IFITM Inhibition Phenotypes of HIV-1

(A) Schematic representation of human IFITM1, IFITM2, and IFITM3 proteins, indicating the localization of a conserved tyrosine at the N terminus of IFITMs 2 (Y19) and 3 (Y20). The localization of the IFITM proteins was assessed using U87 cells transduced with the indicated HA-tagged IFITM. Cells were stained with anti-HA (green) antibody and co-stained with early or late endosomal markers anti-EEA1 (red) or anti-CD63 (red).

(B) Top: localization of HA-tagged IFITM2-Y19F or IFITM3-Y20F in U87 cells as in (A). Bottom: localization of HA-tagged IFITM2 or IFITM3 in U87, with or without small interfering RNA (siRNA)-mediated knockdown of AP-2, is shown.

(C) As in Figure 1C, pseudotyped viruses were produced with wild-type YU2 and Hxb2 envelopes as well as V3 loop swaps. The percentage of infected cells, in the presence of either wild-type or mutant IFITM protein, was determined by flow cytometry (***$p < 0.001$; **$p < 0.01$; *$p < 0.05$; ns, $p > 0.05$, unpaired two-tailed t test).

(D) U87/CD4/CXCR4 cells expressing IFITM2 or IFITM2-Y19F were infected with 89.6 env-pseudotyped HIV-1 vector (left) or with full-length HIV-1 89.6 at an MOI of 0.05 (right), and they were analyzed as in Figures 1A and S1D, respectively (***$p < 0.001$, unpaired two-tailed t test).

(E) The effect of AP2 siRNA knockdown in U87/CD4/CXCR4 on the inhibition of 89.6 proviral replication. Cells were treated with control or AP2-specific SMARTpool siRNA and infected with 89.6. Then 48 hr post-infection, supernatants were assessed for viral production, and lysates were examined for the expression of AP2 and loading control HSP90 by western blot. Statistical significance was determined by using an unpaired two-tailed t test (***$p < 0.001$; **$p < 0.01$; *$p < 0.05$; ns, $p > 0.05$).

(F) Effect of endocytosis inhibitors dynasore and Pitstop2 on IFITM restriction of pseudotyped virus entry. U87/CD4/CoR cells expressing the indicated IFITM were exposed to dynasore (80 μM), Pitstop 2 (30 μM), or DMSO as a control, for 30 min prior to infection with the indicated env-pseudotyped viruses. The percentage of GFP$^+$ cells was determined by flow cytometry (***$p < 0.001$; **$p < 0.01$; *$p < 0.05$; ns, $p > 0.05$, unpaired two-tailed test).

All error bars represent ± SEM (n = 3). See also Figure S2.

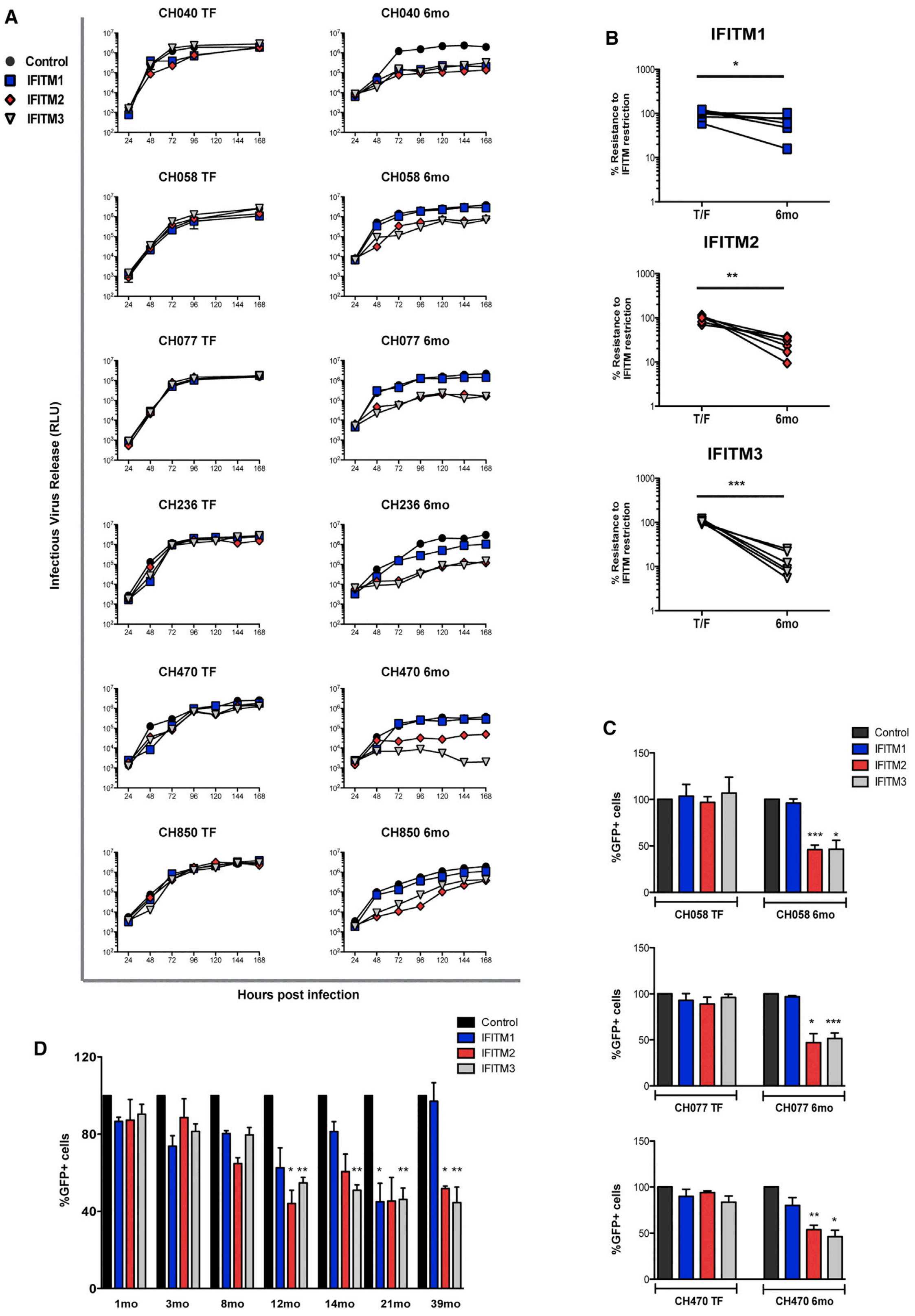

(legend on next page)

the envelopes contained an IFN sensitivity determinant (Figure 6A). We next employed CRISPR-Cas9 lentiviral vectors targeting individual IFITMs or an irrelevant control (luciferase). While each guide effectively knocked out the specific IFITM targeted, the degree of homology between them and, presumably, their chromosomal positioning and proximity resulted in effective deletion of IFITM2 by IFITM3 guides and vice versa, as well as reduced expression of both IFITMs 2 and 3 by the IFITM1 guide (Figure 6B). Using a TF/6-month pair, CH077, that only displayed IFITM2 and 3 sensitivity, we found that, in U87/CD4/CCR5 cells, only the 6-month variant displayed IFN-1 sensitivity (Figure 6C). Moreover, treatment with the IFITM CRISPR guides relieved this IFN-induced restriction, with no effect on the TF virus. This indicates that expression of IFITMs contributes to the different IFN sensitivities of TF and 6-month virus.

We next moved into primary human $CD4^+$ T cells using lentivirally delivered small hairpin RNAs (shRNAs). Again, due to their high degree of homology, effective shRNAs displayed a degree of cross-knockdown, particularly between IFITMs 2 and 3 (Figure 6D). However, using cells from three independent donors, we examined cumulative replication of CH040 and CH470 after 5 days in the presence or absence of 500 U/mL universal IFN-1. The TF variants of both viruses displayed a weak sensitivity to IFN-1 treatment, with little or no effect of any of the shRNAs. In contrast, replication of the 6-month variants was markedly reduced by IFN (Figure 6E). In the case of CH040, this IFN sensitivity was rescued by shRNAs targeting IFITMs 2 or 3, but not IFITM1, consistent with the replication of this virus in our engineered cells (Figure 3). For CH470, knockdown of IFITM1 also contributed to the rescue. Importantly, for CH040, the NAb revertant virus behaved like the TF virus, which is IFN resistant and unresponsive to IFITM knockdown (Figure 6E). Taken together, these data demonstrate that IFITMs are relevant HIV-1 antiviral factors in primary human cells and substantially contribute to the IFN-1 sensitivity of chronic phase viruses in a manner dependent on escape mutations of the envelope to host immune responses. These data also suggest that IFITM resistance in TF viruses is an important attribute in vivo.

DISCUSSION

We have re-examined the roles of IFITMs in the restriction of HIV-1 entry, and we present evidence that is consistent with them having a role in the innate immune response against the virus during the earliest stages of acute HIV-1 infection. Specifically, we found that TF HIV-1 is IFITM resistant but that the virus acquires sensitivity progressively over time. This increased IFITM sensitivity correlates with the acquisition of Env mutations previously shown to mediate escape from early autologous neutralization (Bar et al., 2012), and, thus, it explains at least some of the IFN-1 sensitivity previously shown to emerge during chronic infection (Fenton-May et al., 2013). Additionally, we document that the patterns of IFITM sensitivity of chronic and lab-adapted isolates of HIV-1 are determined by their co-receptor use and the subcellular localization of the IFITM itself. These data strongly suggest that co-receptor usage affects the route of cellular entry such that the virus fuses in different subcellular compartments.

The mechanism of action of IFITMs remains poorly understood. For most pH-dependent viruses, IFITM3 in particular induces the accumulation of virions in late endosomes because of a lack of proper envelope-mediated fusion. This has been proposed to be as a result of IFITM3 interaction with VAMP-associated protein (VAPA) leading to the dysregulation of sterol trafficking (Amini-Bavil-Olyaee et al., 2013), although this has yet to be confirmed by others. In keeping with the notion of membrane modification, the antifungal drug amphotericin B, which modulates cholesterol synthesis, inhibits IFITM3 restriction of IAV (Lin et al., 2013). By contrast, the effects of IFITMs on HIV-1 have been less well defined. While all have been shown to have effects on viral infectivity (Lu et al., 2011), the small differences observed in one-round infections have led to speculations of altered envelope processing/incorporation in the producer cells (Yu et al., 2015) or viral membrane incorporation (Compton et al., 2014; Tartour et al., 2014). In our cell lines expressing physiologically relevant IFITM levels, we saw no envelope-processing defects, and, despite the IFITM incorporation into nascent virions, the infectivity phenotypes we observed correlate only with the expression/localization of the IFITM in the target cell.

The dependence of viral restriction on IFITM localization has important implications for HIV-1 entry and tropism. The sequential engagement of CD4 and co-receptor leads to complex conformational changes in Env that activate membrane fusion (Wilen et al., 2012). Since these events are pH independent, it is widely assumed that fusion takes place at the plasma membrane. However, it has been proposed that HIV-1 enters by dynamin-dependent endocytic processes (Miyauchi et al., 2009). This is countered by observations that, while such entry may be observable, the endocytosis is not necessary for productive infection (Herold et al., 2014; Pelchen-Matthews et al., 1995). The restriction of X4 and some R5 viruses by IFITM2 or IFITM3, which is dependent on the AP-2-binding site, suggests that the

Figure 3. TF Virus Replication Is Resistant to IFITMs, with Sensitivity Arising at 6 Months

(A) U87/CD4/CCR5 IFITM-expressing cells were infected with the indicated TF molecular clones or cognate 6-month variant at an MOI of 0.05, and infection was assayed with supernatants harvested every 24 hr for 7 days. Viral production was measured on HeLa-TZMbl indicator cells. Data represent a summary of three independent experiments.

(B) Comparison of the relative cumulative replication in 3A at the 96-hr time point was analyzed by a paired Mann-Whitney test (***$p < 0.001$; **$p < 0.01$; *$p < 0.05$; ns, $p > 0.05$).

(C) HIV-1 vectors pseudotyped with envelopes of CH058, CH077, and CH470 TF and 6-month viruses were used to infect U87/CD4/CCR5 IFITM cells, analyzed as in Figure 1A. The percentage of infected cells was determined by flow cytometry, and results shown represent three independent infection experiments (***$p < 0.001$; **$p < 0.01$; *$p < 0.05$; ns, $p > 0.05$, unpaired two-tailed test).

(D) Sequential envelopes derived from the CAP256 patient (CAP256.1MO.C7J [1 month], CAP256.3MO.C9 [3 months], CAP256.8MO.31 [8 months], CAP256.12MO.1 [12 months], CAP256.14MO.5b [14 months], CAP256.21MO.A1 [21 months], and CAP256.39MO.10 [39 months]) were used to produce pseudotyped HIV-1 vectors and infect of U87/CD4/CCR5 IFITM cells (**$p < 0.01$; *$p < 0.05$; ns, $p > 0.05$, unpaired two-tailed test).

All error bars represent ± SEM ($n = 3$). See also Figure S3 and Table S1.

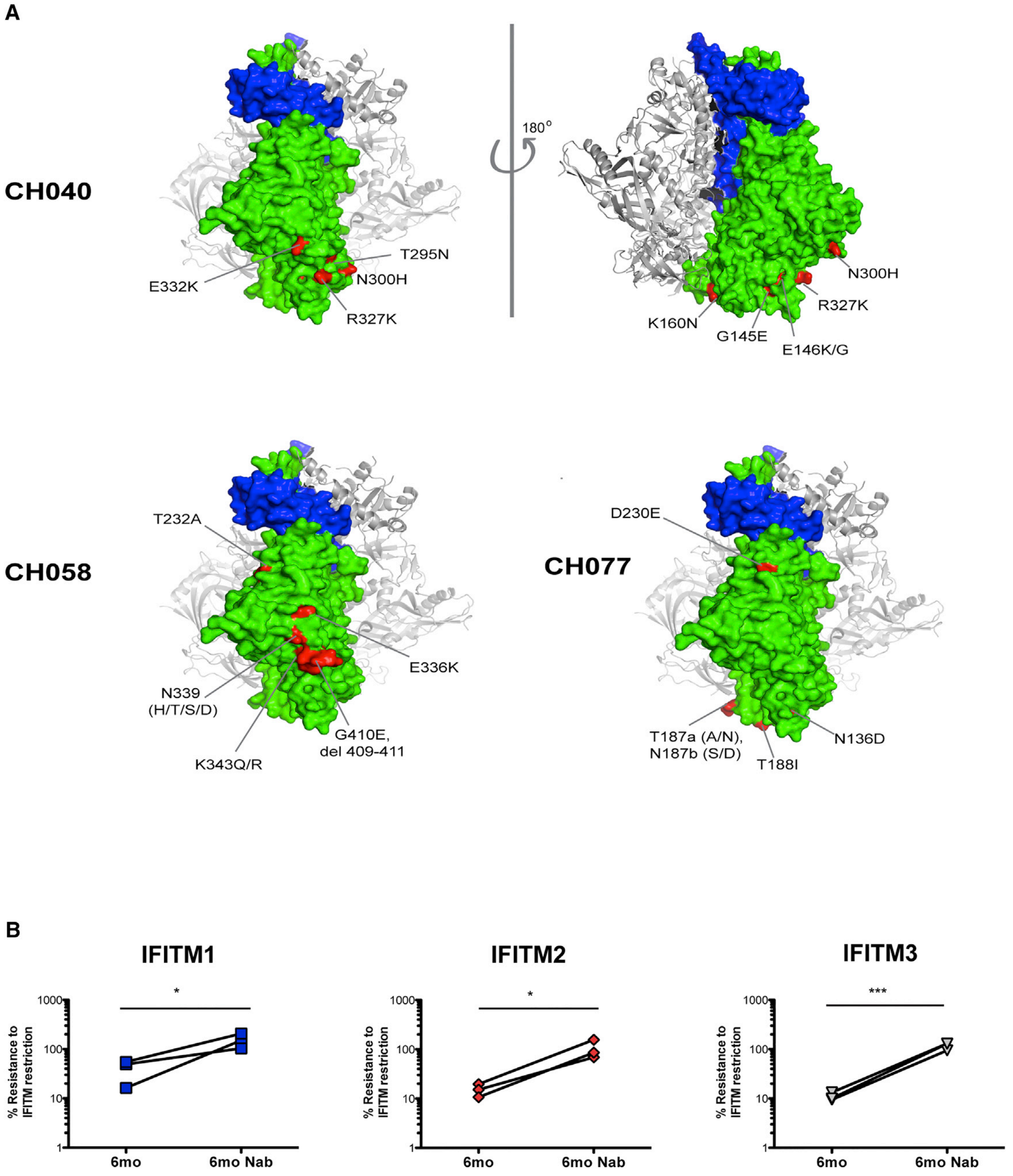

Figure 4. Reversion of Neutralization Escape Mutants in 6-Month Viruses Restores IFITM Resistance

(A) The gp120:gp41 trimer structures show the NAb escape mutations of CH040, CH058, and CH077 in red. Gp120, green; gp41, blue. Images were drawn using PDB: 5ACO in Pymol.

(B) U87/CD4/CCR5 IFITM cells were infected with the indicated 6-month chronic viruses or those containing NAb escape reversion mutations at an MOI of 0.05. Cumulative replication over a period of 96 hr was assayed for viral production on HeLa-TZMbl indicator cells as in Figures 3A and 3B.

Statistics were performed using a paired Mann-Whitney test (***$p < 0.001$; **$p < 0.01$; *$p < 0.05$; ns, $p > 0.05$). See also Table S2.

subcellular location of viral entry among different HIV-1 strains is variable. These observations do not necessarily mean that all IFITM2/3-sensitive viruses fuse in endosomes. Both IFITMs 2 and 3 accumulate in endosomes after AP-2-mediated internalization from the plasma membrane, and so there will always be a pool at the cell surface. However, they will be associated with endocytic domains such as clathrin-coated pits; by contrast, the tyrosine-mutated IFITM2/3 will not be. Therefore, the pattern of IFITM restriction implies that different viruses enter cells at spatially distinct localizations. That this is dependent on X4 usage or CD4 density (in the case of the 6-month viruses) suggests that receptor engagement and/or trafficking is a prime determinant of entry site. It is known that CCR5 and CXCR4 traffic differently when engaged in ligand-dependent signaling

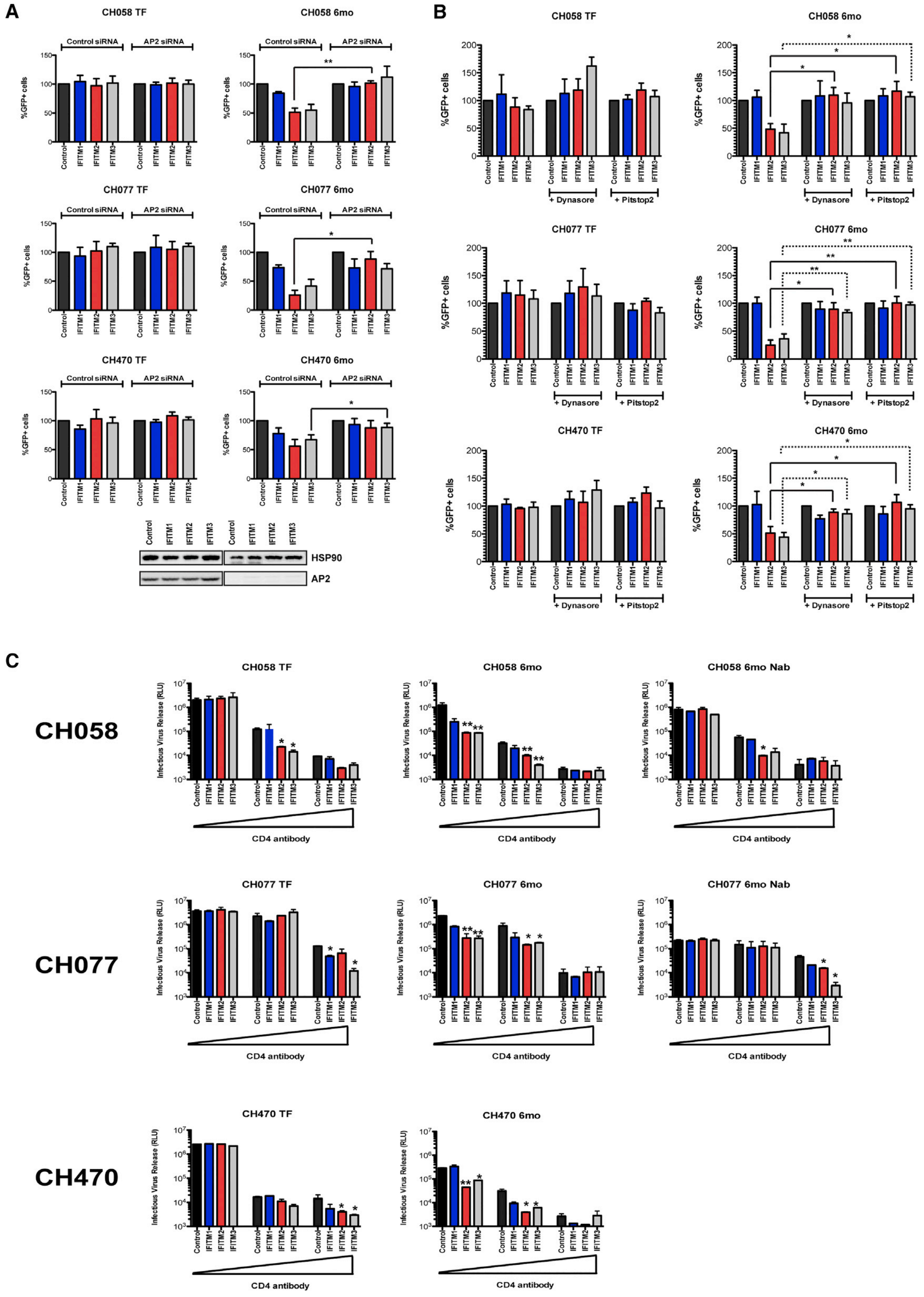

Figure 5. Effect of Endocytosis Inhibitors and Surface CD4 Levels on IFITM Restriction of TF and 6-Month Viral Entry

(A) U87/CD4/CCR5 IFITM cells were treated with control or AP-2-specific siRNAs for 48 hr and then infected with the indicated HIV-1-pseudotyped vector. GFP^+ cells were analyzed as in Figure 1A (***p < 0.001; **p < 0.01; *p < 0.05; ns, p > 0.05, unpaired two-tailed test). Knockdown was assessed by western blot for AP-2μ.

(B) As in (A) but the cells were pretreated with DMSO, dynasore, or Pitstop2.

(legend continued on next page)

(Signoret et al., 1998), and there is much suggestive evidence that co-receptor engagement by incoming HIV-1 also induces signaling (Wu and Yoder, 2009). Interestingly, the entry of the R5 user YU2 could be rescued from IFITM1 by endocytic blockade, indicating that modulation of receptor trafficking in the plane of the plasma membrane may affect viral entry site at the cell surface. Our data therefore suggest that the patterns of IFITM restriction can be used to dissect the role of endocytic trafficking in HIV-1 entry, which has important implications for understanding cell tropism and co-receptor switching.

Unlike the restriction factors APOBEC3G, tetherin, and SAMHD1, which are directly counteracted by lentiviral-encoded accessory proteins, there is no direct evidence for the relevance of any other specific ISG-mediated restriction in vivo, despite abundant evidence that many can target specific stages of the replication cycle in cultured systems (Doyle et al., 2015). However, the acquisition of IFN-1 sensitivity by chronic viruses is highly suggestive that some must do so at physiological expression levels (Fenton-May et al., 2013; Parrish et al., 2013). ISGs that are directly antiviral often target viral structures (the cell-derived membrane) or processes (reverse transcription), which cannot simply be mutated (Doyle et al., 2015). As such, the entry pathway of the virus is a perfect target for the host.

The most striking observation in our study is that, while we see varying IFITM sensitivities for lab-adapted and chronic strains of HIV-1, the TF virus envelope proteins are almost uniformly resistant to their activities. The acquisition of sensitivity to IFITMs over the ensuing months strongly suggests that their avoidance is a requirement for HIV-1 to be successfully transmitted. The mapping to residues exposed on the outer faces of the envelope trimers and the timing of the acquisition itself suggest that immune escape mutations might be driving IFITM sensitivity. Broadly NAbs are generally observed only after several years of infection (Burton and Mascola, 2015). However, complement-fixing anti-Env antibodies are detectable coincident with the onset of T cell immunity (Aasa-Chapman et al., 2004, 2005). Furthermore, while exhibiting limited breadth, autologous neutralizing responses in the first 6 months exert sufficient pressure on the envelope to select for escape variants that become fixed in the viral population (Bar et al., 2012; Moody et al., 2015). Any adaptive mutation in a given viral protein may lead to a functional change. NAbs in particular can block HIV-1 receptor engagement (i.e., blocking the CD4-binding site) or can prevent receptor-binding-induced structural changes required for cell fusion (exemplified by MPER-binding Abs) (Burton and Mascola, 2015). Thus, escape from NAbs may impact on these receptor-driven rearrangements and, by extension, the route of cellular entry as we saw for the 6-month viruses.

In keeping with the above, Envs from chronic viruses have been shown to be less sensitive to CD4bs NAbs and to exhibit differences in CCR5 engagement compared to Envs from TF viruses (Wilen et al., 2011). Whether IFITM sensitivity is a general feature of NAb escape or specific to only certain epitopes and whether these immune pressures select for additional compensatory changes require further study. It has been demonstrated that TF viruses have ~1.5-fold more virion-associated Env than chronic viral isolates (Parrish et al., 2013). Whether this is a reflection of increased virion incorporation or trimer stability is not clear. Since IFITM sensitivity is modulated by CD4 engagement, it is possible that Env density is a contributing factor to IFITM resistance. However, previous studies of fusion kinetics between TF and chronic envelopes have not found a significant difference (Wilen et al., 2011). Furthermore, while not analyzed here, we cannot exclude the possibility that Env escape mutations in response to CD8+ T cell pressure in the same patients (Liu et al., 2013) could not have also had a structural impact that led to IFITM sensitivity.

TF viruses entering a naive host do so in the absence of pre-existing adaptive immunity, but they must avoid innate responses to allow sufficient time to establish a systemic infection. Thus, efficient cell entry into activated mucosal $CD4^+$ T cells is an absolute requirement for the virus. We suggest that viruses that successfully transmit are those that can fuse efficiently at the cell surface of cells expressing high CD4 levels. This would endow such viruses with a relative resistance against IFN-induced factors such as IFITMs, and it would particularly facilitate cell-to-cell spread in the face of the robust IFN-1 response that occurs during acute infection (Stacey et al., 2009). However, these requirements change during chronic infection when the virus has to escape from pressures by the adaptive immune system.

Amino acid changes in Env that allow escape from such adaptive responses may impact envelope function in such a way that viral entry routes and, thus, restriction by IFITMs may change. It is interesting to note that for three TF/6-month pairs used here, NAb escape mutations were associated with a reduced ability to replicate in CD4 T cells (Bar et al., 2012). Since activated CD4 T cells express at least some IFITMs, it may well be that fitness costs incurred by immune escape mutations reflect the associated loss of effectively counteracting antiviral restriction. In the case of the TF/6-month pairs tested here, IFITM restriction in primary $CD4^+$ T cells correlates with the IFN sensitivity of the chronic virus. This would argue that, after the establishment of chronic infection, the selective pressure of factors such as IFITMs is no longer sufficient to impact viral replication or, more likely, the counter-selection by adaptive immune responses is so strong that the effects of IFN-1-induced factors like IFITMs on viral replication become tolerable.

Interestingly, there is evidence that IFN resistance increases again during the late stages of HIV-1 infection (Fenton-May et al., 2013). Understanding whether this inversely correlates with the strength of the adaptive immune response will be particularly interesting. We therefore propose that IFITM restriction and its inverse relationship with adaptive immune escape might be a paradigm for demonstrating that a given ISG that inhibits HIV-1 in vitro is likely to be of relevance in vivo.

Our data indicate that IFITMs represent major effectors of the innate immune response to HIV-1 in vivo. Because of their

(C) U87/CD4/CCR5 IFITM-expressing cells were infected with TF viruses and their matched chronic pairs at an MOI of 0.5 in the presence of 0, 10, or 100 ng/mL of CD4-blocking antibody SK3 for 6 hr. Then 48 hr post-infection, virus production was measured by infection of HeLa-TZMbl indicator cells (**$p < 0.01$; *$p < 0.05$; ns, $p > 0.05$, unpaired two-tailed t test).
All error bars represent ± SEM (n = 3). See also Figure S4.

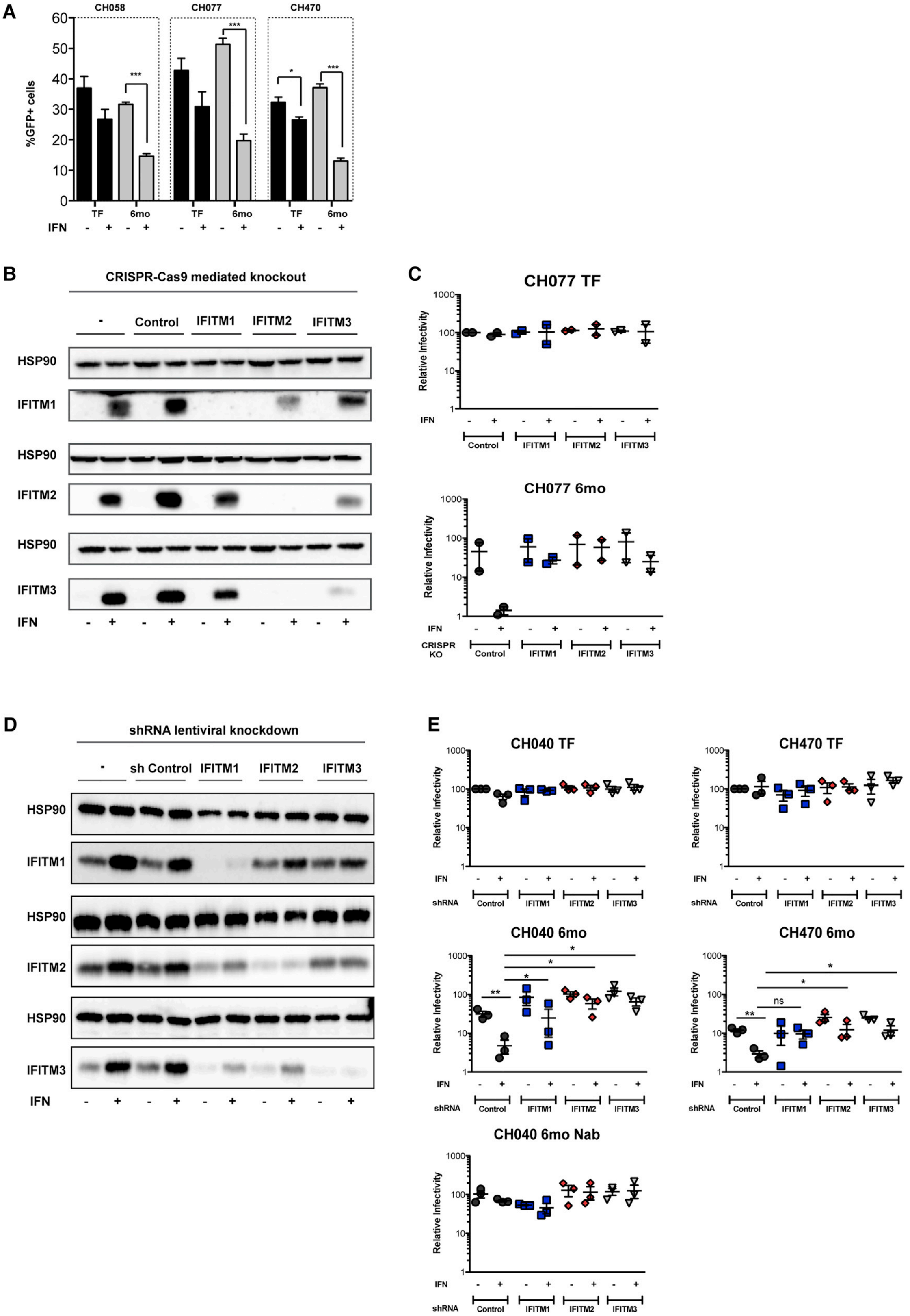

(legend on next page)

sequence similarity, we were unable to find potent shRNAs that could selectively knockdown endogenous IFITMs individually in primary cells. However, the patterns of restriction observed in ectopically expressing cell lines would suggest that the endosomal IFITMs 2 and 3 are the most contributing to the IFN sensitivity of chronic viruses. The rs12252-C polymorphism in IFITM3 is predicted to result in a variant lacking the tyrosine-based endocytic signal. This potentially would lead to surface expression of IFITM3 (Everitt et al., 2012). Recently this polymorphism was associated with faster disease HIV-1 progression in China (Zhang et al., 2015). Interestingly, there was no enhanced susceptibility to infection between different *Ifitm3* genotypes. This observation is entirely consistent with our data that both TF and 6-month viruses are resistant to surface IFITM3. We predict that the viruses that escape Env-directed adaptive immune responses in subjects carrying the rs12215-C polymorphism will be less susceptible to host innate restriction, enhancing their replication and therefore disease progression.

EXPERIMENTAL PROCEDURES

Plasmids and Reagents

Full details of HIV-1 molecular clones, HIV-1 Env plasmids, and IFITM expression constructs are described in the Supplemental Experimental Procedures. Human IFITM1, IFITM2, and IFITM3 were cloned into pLHCX retroviral vector (Clontech Laboratories). Mutants IFITM2-Y19F and IFITM3-Y20F were generated by site-directed mutagenesis using the parental pLHCX IFITM1, 2, or 3 constructs as templates. IFITM1, IFITM2, IFITM3, and Y19F or Y20F mutants thereof were all HA tagged by PCR-based mutagenesis, again using the parental pLHCX-IFITM1, 2, or 3 as templates.

Cell Culture

The 293T-, HeLa-TZMbl-, and U87-based cell lines were cultured, transfected, or infected as described in the Supplemental Experimental Procedures. Human primary $CD4^+$ T cells were isolated from peripheral blood mononuclear cells (PBMCs) of healthy human donors and cultured and infected as outlined in the Supplemental Experimental Procedures.

Virus and HIV-1 Env Pseudotyped Viral Vector Production

To generate virus and vector stocks, 293T cells were transfected with 10 μg HIV-1 molecular clones plasmid or three-plasmid mix of pCSGW (GFP-encoding vector genome), pCRV1-HIV-1 GagPol packaging vector, and pSVIII or pCRV1 plasmids encoding various HIV-1 envelope glycoproteins. Supernatants were harvested and filtered 48 hr post-transfection and titers were calculated by standard methods. Full details are given in the Supplemental Experimental Procedures.

shRNA Lentiviral Knockdown and CRISPR Knockout of IFITM Expression

Silencing of IFITM expression was mediated either by lentiviral shRNA knockdown in primary human $CD4^+$ T cells or CRISPR knockout in U87 $CD4^+$ $CCR5^+$ cells, as outlined in the Supplemental Experimental Procedures.

Infections

U87/CD4/$CXCR4^+$ or U87/CD4/CCR5 cells stably expressing IFITMs 1, 2, or 3 or mutants thereof were infected with the indicated HIV-1 molecular clone at an MOI of 0.05. Media were replaced 8 hr post-infection, and culture supernatants were harvested every 24 hr post-infection for a total of 120 hr. Infectious viral release was determined by infecting HeLa-TZMbl indicator cells and 48 hr post-infection assaying for virus release by measuring chemiluminescent β-galactosidase activity, using the Tropix Galacto-Star system (Applied Biosystems) according to the manufacturer's instructions. For one-round virus release assays, cells were infected with the indicated HIV-1 molecular clone at an MOI of 0.5. Viral production was measured for supernatants harvested at 48 hr post-infection on HeLa-TZMbl indicator cells, as above. For env-pseudotyped viral vector entry assays, the same cells were infected with a fixed dose of HIV-1 viral vectors at an MOI of 0.2 for 48–72 hr prior to analysis for GFP expression by flow cytometry.

Activated $CD4^+$ T cells, transduced with the appropriate shRNA lentiviral vectors, were infected at an MOI of 0.1; then 8–12 hr post-infection, media were replaced. Supernatants were harvested every 72, 120, and 168 hr post-infection, and virus particle production was assessed on HeLa-TZMbl cells as described previously.

CD4 Competition Assays

U87-MG $CD4^+$ $CCR5^+$ cells were infected at an MOI of 0.5 with the indicated HIV-1 molecular clone/anti-human CD4 (SK3 clone, BioLegend) antibody mix. Anti-human CD4 antibody was used at concentrations of 100, 10, and 0 ng/mL. Then 48 hr post-infection, supernatants were harvested and used to infect HeLa-TZMbl cells, assaying for any dose-dependent reduction in virus release as detailed in the Supplemental Experimental Procedures.

Ethics Statement

Ethical approval to use blood from healthy donors was granted by King's College London Infectious Disease BioBank Local Research Ethics Committee (under the authority of the Southampton and South West Hampshire Research Ethics Committee—approval REC09/H0504/39), approval number SN-1/6/7/9.

SUPPLEMENTAL INFORMATION

Supplemental Information includes Supplemental Experimental Procedures, five figures, and two tables and can be found with this article online at http://dx.doi.org/10.1016/j.chom.2016.08.006.

Figure 6. IFITM2 and IFITM3, in Particular, Contribute to the Inhibition of HIV-1 Replication in Primary $CD4^+$ T Cells

(A) HIV-1 vectors pseudotyped with TF and 6-month envelope variants were used to challenge U87/CD4/CCR5 cells that had been pretreated overnight with 1,000 U/mL universal IFN-1. Infected cells were analyzed by flow cytometry 48 hr later (*$p < 0.05$; unpaired; unpaired two-tailed t test).

(B) U87/CD4/CCR5 cells were transduced with IFITM-specific or control lentiCRISPR-p2A-GFP vectors sufficiently to achieve 80%–98% GFP^+. The efficiency of CRISPR knockout of IFITM expression, in the absence or presence of IFN-1 (1,000 U/mL), was determined by western blotting. HSP90 served as a loading control.

(C) Cells from (B) were challenged with CH077 at an MOI of 0.05 in the presence of absence of 1,000 U/mL IFN-1, and cumulative replication at 96 hr was determined as previously described.

(D) Primary $CD4^+$ T cells were transduced with shRNAs targeting IFITM1, 2, or 3 or a control shRNA and cultured for 72 hr. Transduced cells were cultured with or without 500 U/mL IFN-1 for 24 hr before determining the efficiency of IFITM knockdown by western blot analysis. HSP90 served as a loading control.

(E) $CD4^+$ T cells, expressing control or IFITM-specific shRNAs, were infected with the indicated TF and chronic matched-pair viruses at an MOI of 0.05. A time course of replication, in the presence or absence of 500 U/mL universal IFN-1, was assayed with supernatants harvested every 24 hr for a total of 5 days. Infectious virus was determined by infection of HeLa-TZMbl indicator cells. Data shown are representative of the replication values at 96 hr, and IFITM shRNA knockdown data are expressed relative to the virus-specific TF control sample. Data shown are representative of three independent donors and independent experiments.

Statistical significance between indicated pairs was determined using an unpaired two-tailed t test (**$p < 0.01$; *$p < 0.05$; ns, $p > 0.05$). All error bars represent ± SEM (n = 3). See also Figure S5.

CellPress

AUTHOR CONTRIBUTIONS

All experiments were performed by T.L.F. with the help of H.W. and K.C. S.S.I., K.D., S.S., P.K., P.B., A.F., and B.H.H. provided reagents, data, and advice. T.L.F. and S.J.D.N. analyzed the data and wrote the manuscript.

ACKNOWLEDGMENTS

We thank Joseph Sodroski for the V3 loop swap envelopes, Dr. Penny Moore for the CAP256 envelope panel, and the NIH AIDS Reagents Repository for reagents. This study was supported by a Wellcome Trust Senior Research Fellowship (WT098049AIA) and a European Research Council (ERC) grant (281698) to S.J.D.N. and grants from the NIH (R01 AI 114266 and R01 AI 111789) to P.B. and B.H.H.

Received: June 15, 2016
Revised: August 4, 2016
Accepted: August 23, 2016
Published: September 15, 2016

REFERENCES

Aasa-Chapman, M.M., Hayman, A., Newton, P., Cornforth, D., Williams, I., Borrow, P., Balfe, P., and McKnight, A. (2004). Development of the antibody response in acute HIV-1 infection. AIDS *18*, 371–381.

Aasa-Chapman, M.M., Holuigue, S., Aubin, K., Wong, M., Jones, N.A., Cornforth, D., Pellegrino, P., Newton, P., Williams, I., Borrow, P., and McKnight, A. (2005). Detection of antibody-dependent complement-mediated inactivation of both autologous and heterologous virus in primary human immunodeficiency virus type 1 infection. J. Virol. *79*, 2823–2830.

Abel, K., Rocke, D.M., Chohan, B., Fritts, L., and Miller, C.J. (2005). Temporal and anatomic relationship between virus replication and cytokine gene expression after vaginal simian immunodeficiency virus infection. J. Virol. *79*, 12164–12172.

Amini-Bavil-Olyaee, S., Choi, Y.J., Lee, J.H., Shi, M., Huang, I.C., Farzan, M., and Jung, J.U. (2013). The antiviral effector IFITM3 disrupts intracellular cholesterol homeostasis to block viral entry. Cell Host Microbe *13*, 452–464.

Bailey, C.C., Kondur, H.R., Huang, I.C., and Farzan, M. (2013). Interferon-induced transmembrane protein 3 is a type II transmembrane protein. J. Biol. Chem. *288*, 32184–32193.

Bar, K.J., Tsao, C.Y., Iyer, S.S., Decker, J.M., Yang, Y., Bonsignori, M., Chen, X., Hwang, K.K., Montefiori, D.C., Liao, H.X., et al. (2012). Early low-titer neutralizing antibodies impede HIV-1 replication and select for virus escape. PLoS Pathog. *8*, e1002721.

Burton, D.R., and Mascola, J.R. (2015). Antibody responses to envelope glycoproteins in HIV-1 infection. Nat. Immunol. *16*, 571–576.

Chesarino, N.M., McMichael, T.M., and Yount, J.S. (2015). E3 ubiquitin ligase NEDD4 promotes influenza virus infection by decreasing levels of the antiviral protein IFITM3. PLoS Pathog. *11*, e1005095.

Compton, A.A., Bruel, T., Porrot, F., Mallet, A., Sachse, M., Euvrard, M., Liang, C., Casartelli, N., and Schwartz, O. (2014). IFITM proteins incorporated into HIV-1 virions impair viral fusion and spread. Cell Host Microbe *16*, 736–747.

Coren, L.V., Shatzer, T., and Ott, D.E. (2008). CD45 immunoaffinity depletion of vesicles from Jurkat T cells demonstrates that exosomes contain CD45: no evidence for a distinct exosome/HIV-1 budding pathway. Retrovirology *5*, 64.

Desai, T.M., Marin, M., Chin, C.R., Savidis, G., Brass, A.L., and Melikyan, G.B. (2014). IFITM3 restricts influenza A virus entry by blocking the formation of fusion pores following virus-endosome hemifusion. PLoS Pathog. *10*, e1004048.

Doria-Rose, N.A., Schramm, C.A., Gorman, J., Moore, P.L., Bhiman, J.N., DeKosky, B.J., Ernandes, M.J., Georgiev, I.S., Kim, H.J., Pancera, M., et al.; NISC Comparative Sequencing Program (2014). Developmental pathway for potent V1V2-directed HIV-neutralizing antibodies. Nature *509*, 55–62.

Doyle, T., Goujon, C., and Malim, M.H. (2015). HIV-1 and interferons: who's interfering with whom? Nat. Rev. Microbiol. *13*, 403–413.

Duenas-Decamp, M.J., Peters, P.J., Repik, A., Musich, T., Gonzalez-Perez, M.P., Caron, C., Brown, R., Ball, J., and Clapham, P.R. (2010). Variation in the biological properties of HIV-1 R5 envelopes: implications of envelope structure, transmission and pathogenesis. Future Virol. *5*, 435–451.

Everitt, A.R., Clare, S., Pertel, T., John, S.P., Wash, R.S., Smith, S.E., Chin, C.R., Feeley, E.M., Sims, J.S., Adams, D.J., et al.; GenISIS Investigators; MOSAIC Investigators (2012). IFITM3 restricts the morbidity and mortality associated with influenza. Nature *484*, 519–523.

Fenton-May, A.E., Dibben, O., Emmerich, T., Ding, H., Pfafferott, K., Aasa-Chapman, M.M., Pellegrino, P., Williams, I., Cohen, M.S., Gao, F., et al. (2013). Relative resistance of HIV-1 founder viruses to control by interferon-alpha. Retrovirology *10*, 146.

Goujon, C., and Malim, M.H. (2010). Characterization of the alpha interferon-induced postentry block to HIV-1 infection in primary human macrophages and T cells. J. Virol. *84*, 9254–9266.

Herold, N., Anders-Ößwein, M., Glass, B., Eckhardt, M., Müller, B., and Kräusslich, H.G. (2014). HIV-1 entry in SupT1-R5, CEM-ss, and primary CD4+ T cells occurs at the plasma membrane and does not require endocytosis. J. Virol. *88*, 13956–13970.

Jia, R., Pan, Q., Ding, S., Rong, L., Liu, S.L., Geng, Y., Qiao, W., and Liang, C. (2012). The N-terminal region of IFITM3 modulates its antiviral activity by regulating IFITM3 cellular localization. J. Virol. *86*, 13697–13707.

Jia, R., Xu, F., Qian, J., Yao, Y., Miao, C., Zheng, Y.M., Liu, S.L., Guo, F., Geng, Y., Qiao, W., and Liang, C. (2014). Identification of an endocytic signal essential for the antiviral action of IFITM3. Cell. Microbiol. *16*, 1080–1093.

Li, K., Markosyan, R.M., Zheng, Y.M., Golfetto, O., Bungart, B., Li, M., Ding, S., He, Y., Liang, C., Lee, J.C., et al. (2013). IFITM proteins restrict viral membrane hemifusion. PLoS Pathog. *9*, e1003124.

Lin, T.Y., Chin, C.R., Everitt, A.R., Clare, S., Perreira, J.M., Savidis, G., Aker, A.M., John, S.P., Sarlah, D., Carreira, E.M., et al. (2013). Amphotericin B increases influenza A virus infection by preventing IFITM3-mediated restriction. Cell Rep. *5*, 895–908.

Ling, S., Zhang, C., Wang, W., Cai, X., Yu, L., Wu, F., Zhang, L., and Tian, C. (2016). Combined approaches of EPR and NMR illustrate only one transmembrane helix in the human IFITM3. Sci. Rep. *6*, 24029.

Liu, M.K., Hawkins, N., Ritchie, A.J., Ganusov, V.V., Whale, V., Brackenridge, S., Li, H., Pavlicek, J.W., Cai, F., Rose-Abrahams, M., et al.; CHAVI Core B (2013). Vertical T cell immunodominance and epitope entropy determine HIV-1 escape. J. Clin. Invest. *123*, 380–393.

Lu, J., Pan, Q., Rong, L., He, W., Liu, S.L., and Liang, C. (2011). The IFITM proteins inhibit HIV-1 infection. J. Virol. *85*, 2126–2137.

Miyauchi, K., Kim, Y., Latinovic, O., Morozov, V., and Melikyan, G.B. (2009). HIV enters cells via endocytosis and dynamin-dependent fusion with endosomes. Cell *137*, 433–444.

Moody, M.A., Gao, F., Gurley, T.C., Amos, J.D., Kumar, A., Hora, B., Marshall, D.J., Whitesides, J.F., Xia, S.M., Parks, R., et al. (2015). Strain-specific V3 and CD4 binding site autologous HIV-1 neutralizing antibodies select neutralization-resistant viruses. Cell Host Microbe *18*, 354–362.

Parrish, N.F., Gao, F., Li, H., Giorgi, E.E., Barbian, H.J., Parrish, E.H., Zajic, L., Iyer, S.S., Decker, J.M., Kumar, A., et al. (2013). Phenotypic properties of transmitted founder HIV-1. Proc. Natl. Acad. Sci. USA *110*, 6626–6633.

Pelchen-Matthews, A., Clapham, P., and Marsh, M. (1995). Role of CD4 endocytosis in human immunodeficiency virus infection. J. Virol. *69*, 8164–8168.

Pineda, M.J., Orton, B.R., and Overbaugh, J. (2007). A TRIM5alpha-independent post-entry restriction to HIV-1 infection of macaque cells that is dependent on the path of entry. Virology *363*, 310–318.

Salazar-Gonzalez, J.F., Salazar, M.G., Keele, B.F., Learn, G.H., Giorgi, E.E., Li, H., Decker, J.M., Wang, S., Baalwa, J., Kraus, M.H., et al. (2009). Genetic identity, biological phenotype, and evolutionary pathways of transmitted/founder viruses in acute and early HIV-1 infection. J. Exp. Med. *206*, 1273–1289.

Sandler, N.G., Bosinger, S.E., Estes, J.D., Zhu, R.T., Tharp, G.K., Boritz, E., Levin, D., Wijeyesinghe, S., Makamdop, K.N., del Prete, G.Q., et al. (2014). Type I interferon responses in rhesus macaques prevent SIV infection and slow disease progression. Nature *511*, 601–605.

Schmitz, C., Marchant, D., Neil, S.J., Aubin, K., Reuter, S., Dittmar, M.T., and McKnight, A. (2004). Lv2, a novel postentry restriction, is mediated by both capsid and envelope. J. Virol. *78*, 2006–2016.

Signoret, N., Rosenkilde, M.M., Klasse, P.J., Schwartz, T.W., Malim, M.H., Hoxie, J.A., and Marsh, M. (1998). Differential regulation of CXCR4 and CCR5 endocytosis. J. Cell Sci. *111*, 2819–2830.

Simmons, G., Reeves, J.D., McKnight, A., Dejucq, N., Hibbitts, S., Power, C.A., Aarons, E., Schols, D., De Clercq, E., Proudfoot, A.E., and Clapham, P.R. (1998). CXCR4 as a functional coreceptor for human immunodeficiency virus type 1 infection of primary macrophages. J. Virol. *72*, 8453–8457.

Smith, S., Weston, S., Kellam, P., and Marsh, M. (2014). IFITM proteins-cellular inhibitors of viral entry. Curr. Opin. Virol. *4*, 71–77.

Stacey, A.R., Norris, P.J., Qin, L., Haygreen, E.A., Taylor, E., Heitman, J., Lebedeva, M., DeCamp, A., Li, D., Grove, D., et al. (2009). Induction of a striking systemic cytokine cascade prior to peak viremia in acute human immunodeficiency virus type 1 infection, in contrast to more modest and delayed responses in acute hepatitis B and C virus infections. J. Virol. *83*, 3719–3733.

Sullivan, N., Sun, Y., Binley, J., Lee, J., Barbas, C.F., 3rd, Parren, P.W., Burton, D.R., and Sodroski, J. (1998). Determinants of human immunodeficiency virus type 1 envelope glycoprotein activation by soluble CD4 and monoclonal antibodies. J. Virol. *72*, 6332–6338.

Tartour, K., Appourchaux, R., Gaillard, J., Nguyen, X.N., Durand, S., Turpin, J., Beaumont, E., Roch, E., Berger, G., Mahieux, R., et al. (2014). IFITM proteins are incorporated onto HIV-1 virion particles and negatively imprint their infectivity. Retrovirology *11*, 103.

Weston, S., Czieso, S., White, I.J., Smith, S.E., Kellam, P., and Marsh, M. (2014). A membrane topology model for human interferon inducible transmembrane protein 1. PLoS ONE *9*, e104341.

Wilen, C.B., Parrish, N.F., Pfaff, J.M., Decker, J.M., Henning, E.A., Haim, H., Petersen, J.E., Wojcechowskyj, J.A., Sodroski, J., Haynes, B.F., et al. (2011). Phenotypic and immunologic comparison of clade B transmitted/founder and chronic HIV-1 envelope glycoproteins. J. Virol. *85*, 8514–8527.

Wilen, C.B., Tilton, J.C., and Doms, R.W. (2012). HIV: cell binding and entry. Cold Spring Harb. Perspect. Med. *2*, a006866.

Wu, Y., and Yoder, A. (2009). Chemokine coreceptor signaling in HIV-1 infection and pathogenesis. PLoS Pathog. *5*, e1000520.

Yount, J.S., Moltedo, B., Yang, Y.Y., Charron, G., Moran, T.M., López, C.B., and Hang, H.C. (2010). Palmitoylome profiling reveals S-palmitoylation-dependent antiviral activity of IFITM3. Nat. Chem. Biol. *6*, 610–614.

Yu, J., Li, M., Wilkins, J., Ding, S., Swartz, T.H., Esposito, A.M., Zheng, Y.M., Freed, E.O., Liang, C., Chen, B.K., and Liu, S.L. (2015). IFITM proteins restrict HIV-1 infection by antagonizing the envelope glycoprotein. Cell Rep. *13*, 145–156.

Zhang, Y., Makvandi-Nejad, S., Qin, L., Zhao, Y., Zhang, T., Wang, L., Repapi, E., Taylor, S., McMichael, A., Li, N., et al. (2015). Interferon-induced transmembrane protein-3 rs12252-C is associated with rapid progression of acute HIV-1 infection in Chinese MSM cohort. AIDS *29*, 889–894.

Cell Host & Microbe, Volume *20*

Supplemental Information

Resistance of Transmitted Founder HIV-1 to IFITM-Mediated Restriction

Toshana L. Foster, Harry Wilson, Shilpa S. Iyer, Karen Coss, Katie Doores, Sarah Smith, Paul Kellam, Andrés Finzi, Persephone Borrow, Beatrice H. Hahn, and Stuart J.D. Neil

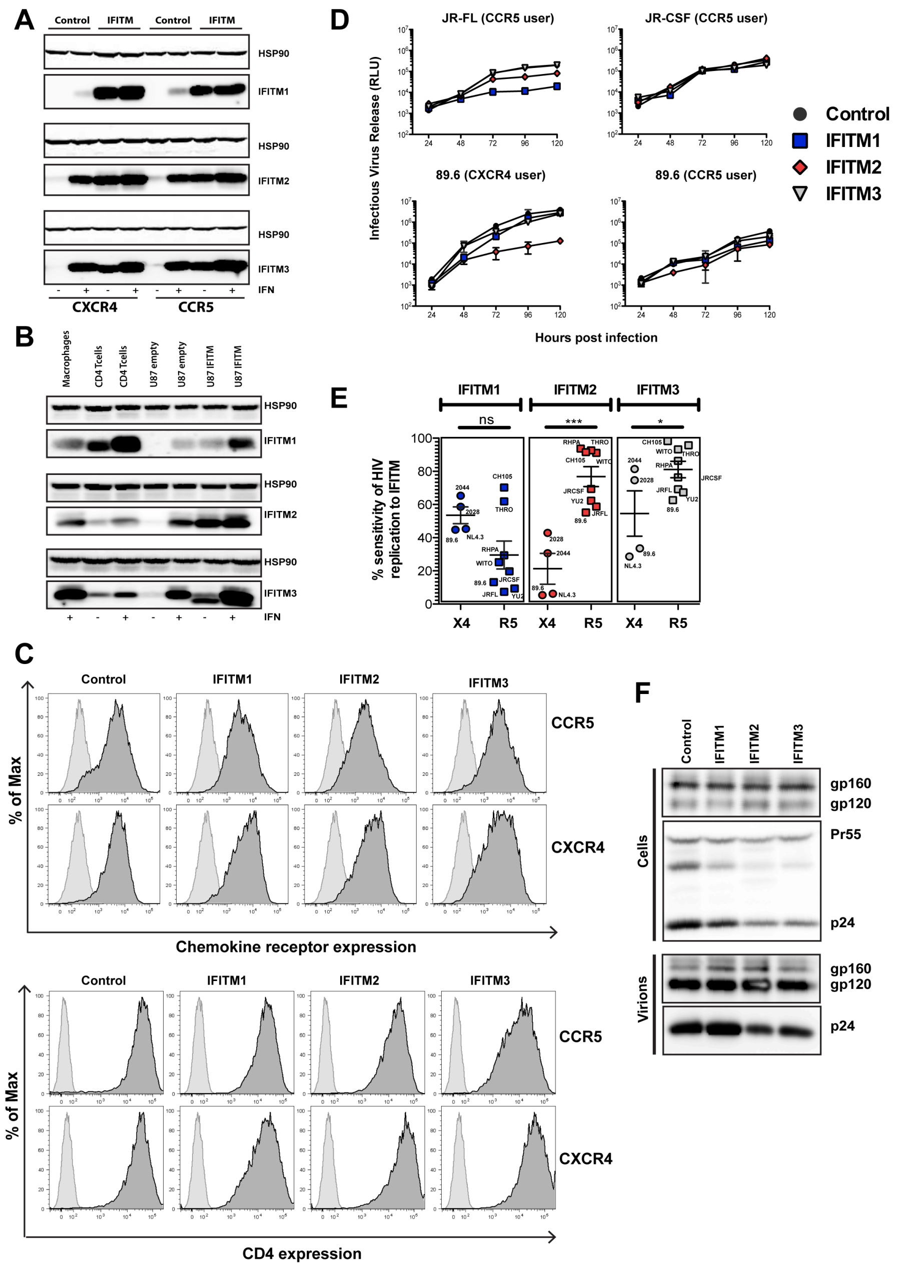

Figure S1, related to Figure 1. IFITM proteins impair HIV-1 infection.

(A) U87 cell lines that stably express CD4 and CCR5 or CXCR4 were transduced with IFITM1, 2 or 3. Control represents cells that were transduced with empty lentiviral vector pLHCX. Expression levels of IFITMs in the absence or presence of 1,000 U/ml interferon-α were determined by western blotting. HSP90 served as a loading control.

(B) The expression levels of IFITMs I stably transduced U87 cells was compared to that of interferon-α (500 U/ml) induced primary human macrophages and CD4+ T-cells. HSP90 served as a loading control.

(C) IFITM proteins do not affect the expression of the entry receptors CD4, CCR5 or CXCR4. Representative data of CCR5 or CXCR4 (upper panel) and CD4 (lower panel) surface expression in the absence or presence of IFITM proteins. Isotype control is in light grey. U87 cells stably expressing the entry receptors and the control empty vector or IFITM proteins were analysed for CD4 and chemokine receptor surface expression (dark grey) by flow cytometry.

(D) Virus replication of different virus isolates in the presence of IFITM proteins in U87/CD4/CoR cells. Data represent a summary of three independent experiments (see also Figure 1B).

(E) Panel as in Figure 1B with viral isolates annotated.

(F) Purified virus particles and cell lysates from U87 $CD4^{+}$-$CXCR4^{+}$ IFITM-expressing cells infected with proviral HIV-1 NL4.3 at an MOI of 0.5 were analysed for differences in envelope processing efficiency by immunoblotting using anti-gp120 and anti-p24.

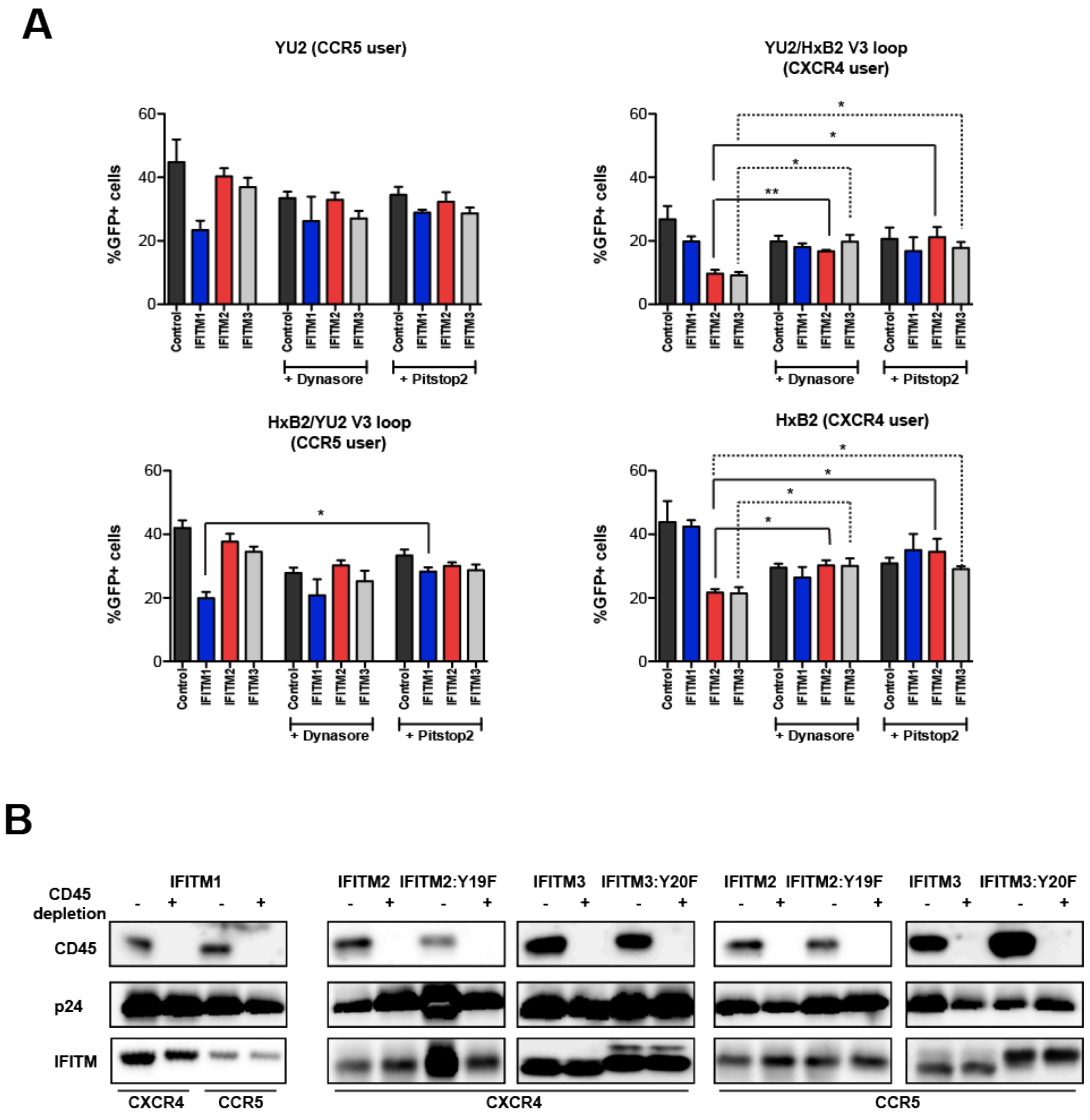

Figure S2, related to Figure 2. IFITM proteins are incorporated into HIV-1 virions but incorporation does not correlate with inhibition phenotypes observed.

(A) Absolute values for data shown in Figure 2F. All error bars represent ±SEM. *** p<0.001, ** p<0.01, *p<0.05, ns p>0.05; unpaired two-tailed test.

(B) U87 CD4$^+$-CXCR4$^+$ or CD4$^+$-CCR5$^+$ cells overexpressing the HA-tagged wild-type IFITM proteins or the mutants IFITM2-Y19F or IFITM3-Y20F were infected with 89.6 provirus. Viral supernatants were harvested and purified through a 20% sucrose cushion before CD45 depletion was conducted to remove exosomes on p24 matched samples. Western blotting was performed using anti-HA (to detect IFITMs), anti-p24 or anti-CD45.

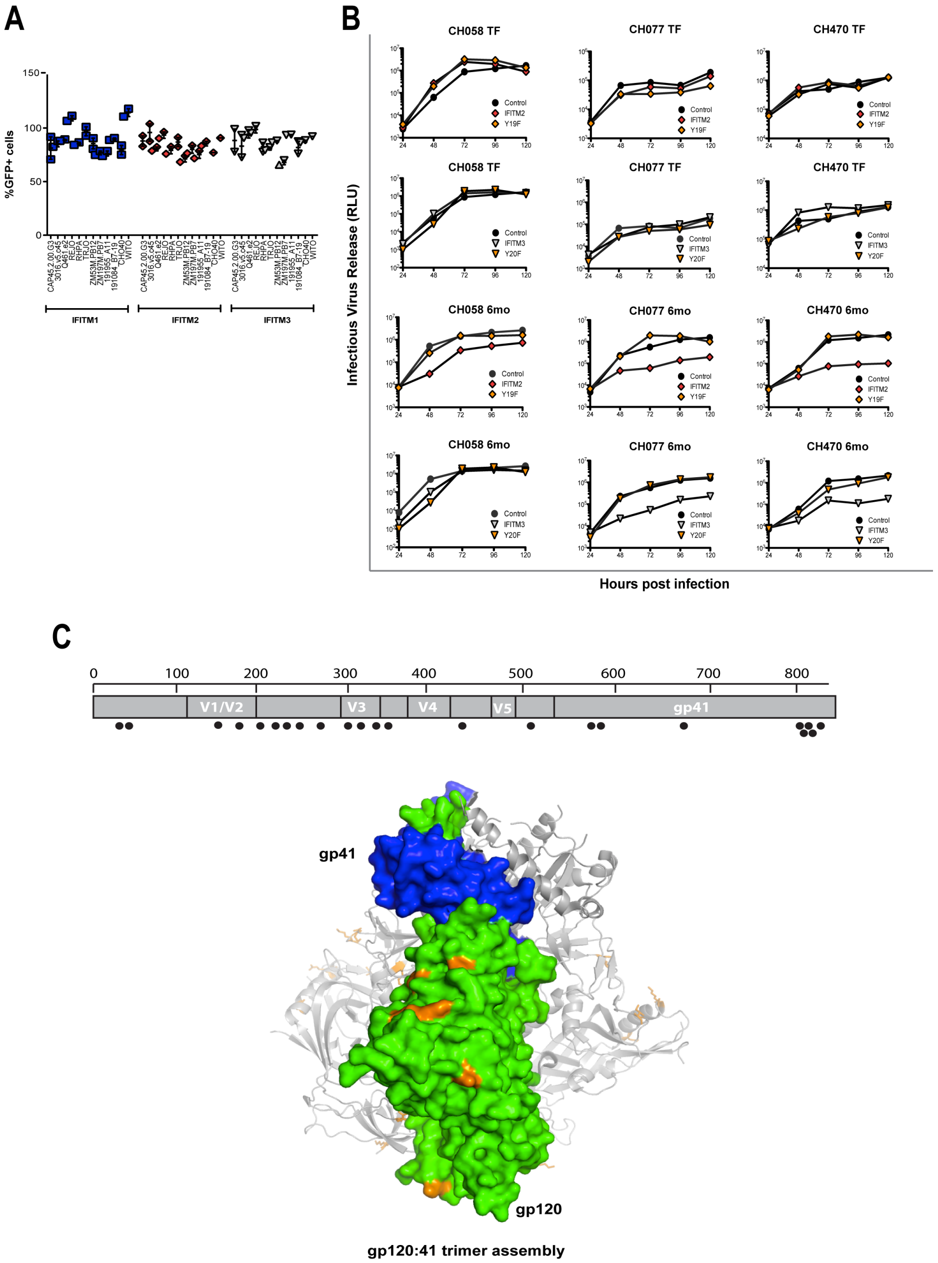

Figure S3, related to Figure 3. Gain in sensitivity to IFITM inhibition correlates with amino acid changes in T/F versus 6mo chronic pairs.

(A) Envelope pseudotyped HIV-1 vectors were produced for the following viruses: CAP45.2.00.G3, 3016.v5.c45, Q461.e2, REJO, RHPA, TRJO, ZM53M.PB12, ZM197M.PB7, 191955_A11, 191084_B7-19, CH040, WITO. U87 CD4^{+}-CCR5^{+} stably expressing the IFITM 1, 2 or 3 proteins were infected for 48hrs before the percentage of infected cells was determined by flow cytometry.

(B) The replication phenotypes of TF and 6-mo chronic clones were determined by infection of U87 CD4^{+}-CCR5^{+} stably expressing the wild-type IFITM 2 or 3 proteins as well as the mutants IFITM2-Y19F or IFITM3-Y20F. A time course of replication over a 5 day period was assayed, with supernatants harvested every 24 hrs. Infectious virus production was determined by infection of HeLa-TZMbl indicator cells.

(C) (Top) Diagrammatic representation of the amino acid differences between the envelope of the TF and 6-mo chronic consensus clones analyzed in this study, i.e. CH040, CH058, CH077, CH236, CH470 and CH850, based on Hxb2 reference sequencing numbering. Dots indicate amino acid position.

(Bottom) Cartoon representation (left) of the top view of the gp120:gp41 trimer. Gp120 is shown in green and gp41 in blue. Amino acid differences between the envelope of the TF and 6-mo clones as in Figure S3A are indicated in orange. The other gp120 and gp41 monomers that comprise the env trimer are shown in grey. Images were drawn using PDB code 5ACO in Pymol.

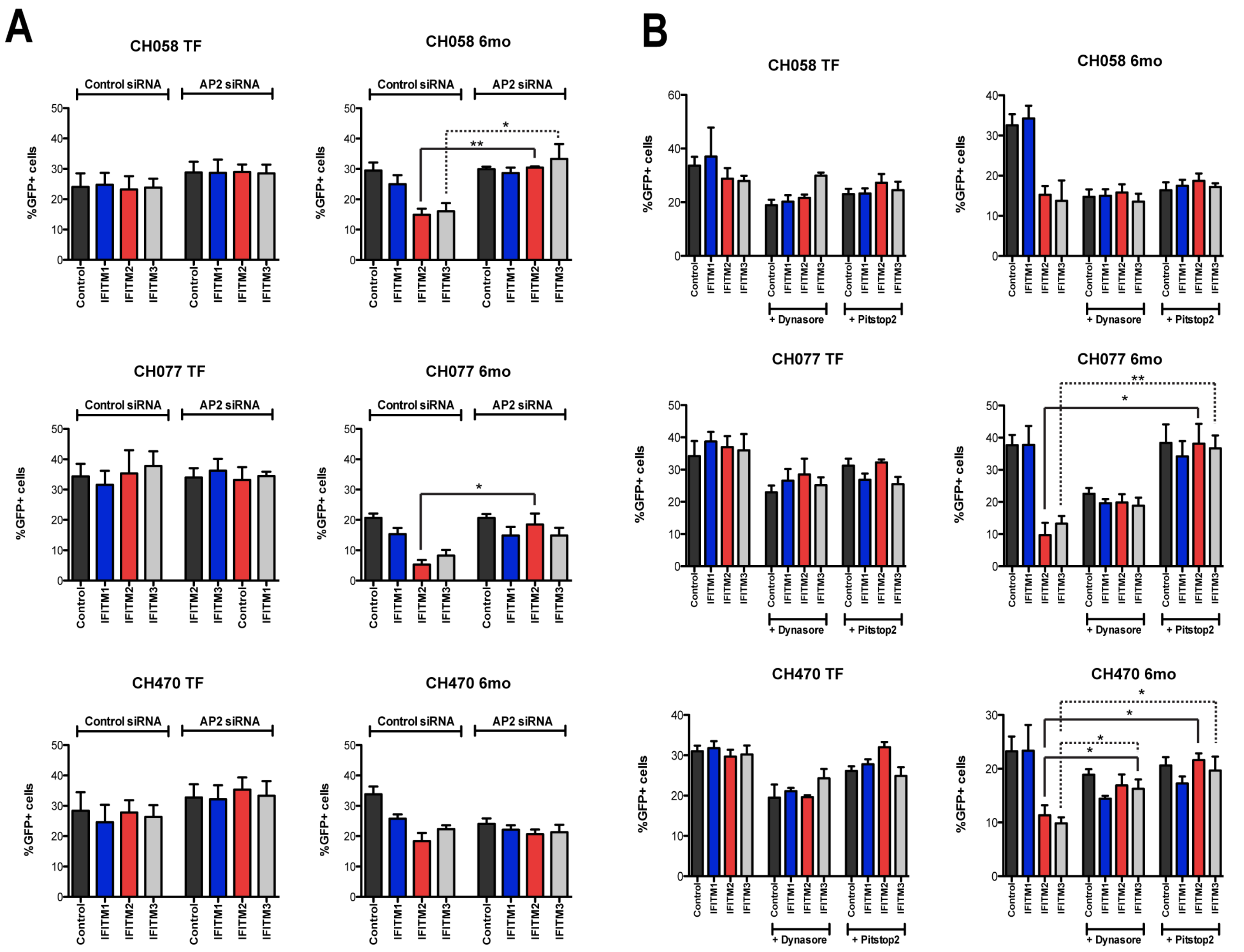

Figure S4, related to Figure 5. Blocking endocytosis by AP2 knockdown and by the use of inhibitors rescues IFITM restriction of 6mo chronic virus isolates.

(A) Absolute values for data shown in Figure 5A. All error bars represent ±SEM. *** p<0.001, ** p<0.01, *p<0.05, ns p>0.05; unpaired two-tailed test.

(B) Absolute values for data shown in Figure 5B. All error bars represent ±SEM. *** p<0.001, ** p<0.01, *p<0.05, ns p>0.05; unpaired two-tailed test.

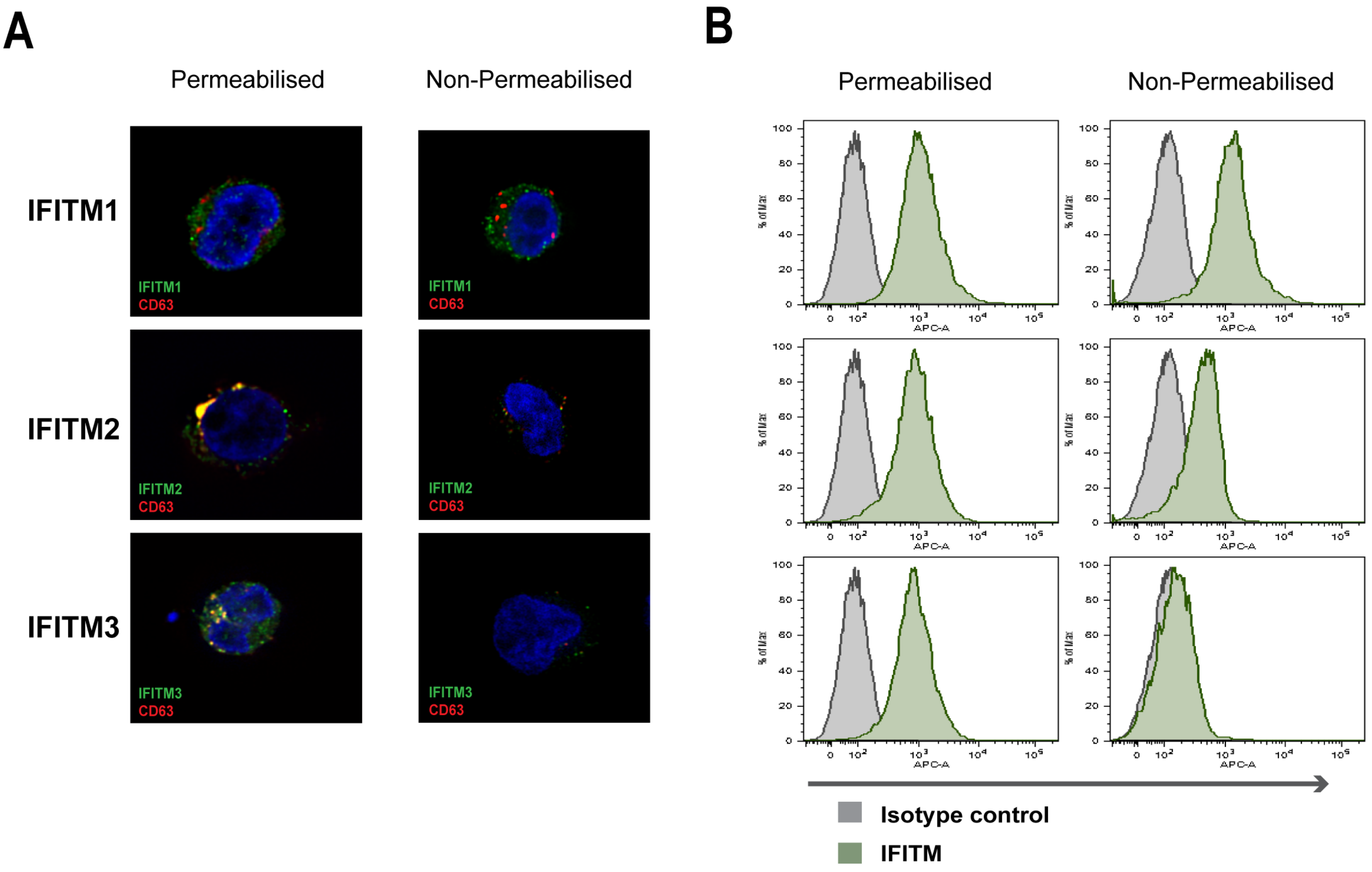

Figure S5, related to Figure 6. Cellular localisation of IFITM proteins in transduced Jurkat T cell lines.

(A) Permeabilised and non-permeabilised Jurkat cells transduced with the HA-tagged IFITMs 1, 2 or 3 proteins. Cells were stained with rabbit anti-HA (green) antibody followed by Alexa 488 conjugated anti-Rabbit IgG. Samples were co-stained with anti-CD63 (red) antibody followed by incubation with Alexa 594-conjugated IgG antibody to assess localization to late endosomes. Panels are of representative examples and images were deconvolved using the AutoQuant X3 software.

(B) Representative data of IFITM protein expression (green) in permeabilised or non-permeabilised Jurkat cell lines stably expressing the HA-tagged IFITMs 1, 2 or 3 proteins as assessed by flow cytometry. Isotype control is in light grey.

**CH058**	T232A **(V3)** *<br>N338D **(C3)** *<br>R579S **(NHR)** *<br>A830T **(E)**
**CH077**	R4K<br>T189N **(V1/V2)**<br>K189E **(V1/V2)** *<br>D229E **(C2)** *<br>D345N **(C2)**<br>R352K **(C3)** *<br>E823G **(E)**
**CH236**	M167V **(V1/V2)**<br>T308A **(V3)** *<br>G509E **(C5)**<br>G516E **(FP)**<br>D676N **(TM)**<br>R800K **(E)**<br>V838I **(E)**
**CH470**	V275I **(C2)** *<br>V588M **(NHR)**<br>A859T **(E)**
**CH850**	I18V<br>Y219H **(C2)** *<br>D816N **(E)**

Table S1, related to Figures 3 and S3C. **Amino acid changes between T/F virus compared with its matched 6 month consensus clone**. Amino acid positions located in gp120 variable and constant regions are indicated with (V) and (C), respectively. Amino acid changes in the fusogenic peptide (FP), N-terminal heptad repeat (NHR), transmembrane domain (TM) or endodomain of gp41 are indicated.
* highlights amino acid positions, shown in Figure S3C.
See also Figure 3.

Table S2, related to Figure 4: Neutralisation escape mutations in Env of 6mo consensus sequences

**CH040 Nab**	G145E<br>E146 K/G<br>K160N<br>T295N<br>N300H<br>R327K<br>E332K
**CH077 Nab**	N136D<br>K186A<br>T187a (A/N)<br>N187b (S/D)<br>T188I<br>D230E
**CH058 Nab**	T232A<br>E336K<br>N339 (H/T/S/D)<br>K343Q/R<br>G410E<br>del 409-411

Supplemental Experimental Procedures

General cell culture

HEK293T cells were obtained from ATCC (American Tissue Culture Collection) and the HeLa-TZMbl reporter cell line, was kindly provided by J. Kappes through the NIH AIDS Reagents Repository Program (ARRP). Human glioblastoma U87-MG cells modified to express HIV-1 receptors CD4, CXCR4 or CCR5 were provided by M. Malim (Goujon et al., 2013)Cells were selected with 1 μg/ml puromycin and 100 μg/ml G418. 293T, HeLa-TZMbl and U87 cell lines were maintained in Dulbecco's modified Eagle's medium (DMEM), 10% fetal calf serum and 20μg/ml Gentamycin (Invitrogen, UK).

Isolation and culture of Primary cells

Human primary $CD4^+$ T cells were isolated from peripheral blood mononuclear cells (PBMCs) of healthy human donors. $CD4^+$ T cells were isolated by density gradient centrifugation through Lymphoprep (Axis-Shield) and obtained by negative selection using the Dynabeads Untouched Human CD4 T Cells kit (Life Technologies) according to the manufacturer's instructions. The purity of the isolated cell population was assessed by flow cytometry for CD4 and was reproducibly found to be > 95%. Cells were cultured in RPMI supplemented with 10% FCS, 30 U/ml recombinant IL-2 (Roche) and 20μg/ml gentamycin at 37°C in 5% CO_2 and activated within 48hrs using Dynabeads Human T-Activator CD3/CD28 beads (CD3/CD28 Dynabeads; Invitrogen) according to the manufacturer's instructions. Prior to infection or post-activation analysis, CD3/CD28 Dynabeads were removed using a DynaMag-2 magnet (Invitrogen), cells washed and resuspended in fresh RPMI-IL-2 supplemented media. When indicated, IFN-α (universal type 1 IFN, PBL InterferonSource) stimulation was performed using 500 U/ml for 24 h before immunoblotting or virus infection.

Ethics Statement

Ethical approval was granted by King's College London Infectious Disease BioBank Local Research Ethics Committee (under the authority of the Southampton and South West Hampshire Research Ethics Committee—approval REC09/H0504/39), approval number SN-1/6/7/9.

IFITM plasmid constructs

Human IFITM1, IFITM2 and IFITM3 were cloned into pLHCX retroviral vector (Clontech). Mutants IFITM2-Y19F and IFITM3-Y20F were generated by site-directed mutagenesis using the parental pLHCX IFITM1, 2 or 3 constructs as templates.

IFITM1, IFITM2, IFITM3 and Y19F or Y20F mutants thereof were all HA-tagged by PCR- based mutagenesis again using the parental pLHCX-IFITM1, 2 or 3 as templates.

HIV-1 infectious molecular clones

The proviral HIV-1 NL4.3, YU2, 89.6, JR-FL, JR-CSF, THRO, WITO, RHPA, and CH105 constructs were obtained from the National Institutes of Health (NIH) AIDS Reagent program.

Virus isolates 2024 and 2028 were kindly provided by P.Clapham.

The infectious transmitted/founder (T/F) HIV-1 molecular clones, their corresponding matched 6 month chronic pairs (6mo) and 6 month chronic neutralisation escape reversion clones (6mo Nab), of CH040, CH058, CH077, CH236, CH470 and CH850 were kindly provided by B.Hahn (co-author).

HIV-1 Env plasmids.

The pSVIII plasmids encoding the Env protein of interest of JR-FL, JR-CSF, CAP45.2.00.G3, 3016.v5.c45, Q461.e2, REJO, RHPA, TRJO, ZM53M.PB12, ZM197M.PB7, 191955_A11, 191084_B7-19, CH040, WITO were kindly provided by Katie Doores (co-author). The JR-FL G366E mutant was generated by site-directed mutagenesis using the parental pSVIII JR-FL Env plasmid as the template.

The following CAP256 envelopes in a pSVIII backbone were provided by P. Moore (Centre for the AIDS Programme of Research in South Africa) [Doria-Rose *et. al.,* 2014]: CAP256.1MO.C7J (1mo), CAP256.3MO.C9 (3mo), CAP256.8MO.31 (8mo), CAP256.12MO.1 (12mo), CAP256.14MO.5b (14mo), CAP256.21MO.A1 (21mo) and CAP256.39MO.10 (39mo).

The pCRV1 plasmids encoding the Env proteins of NL4.3, 89.6, YU2, CH058 T/F, CH058 6mo, CH077 T/F, CH077 6mo, CH470 T/F and CH470 6mo were generated by PCR amplification and insertion via EcoRI and NotI restriction sites.

pSVIII plasmids containing the chimeric envelopes of YU2 and Hxb2 with V3 loop substitution, i.e. YU2/Hxb2 V3 loop and Hxb2/YU2 V3 loop were kindly provided by J. Sodroski [ref].

HIV-1 molecular clone virus production

293T cells plated on 10cm dishes were transfected, using 1 mg/ml polyethyleneimine (Polysciences), typically with 10μg of the HIV-1 molecular clones. Media was changed 8-16 hours post transfection and viral supernatants harvested and filtered 48 hours post-transfection. For concentrated stocks, supernatants were incubated at 37°C for 2 hrs in the presence of 10 μM $MgCl_2$ and 20 U/ml DNAse (Roche) before concentration through a 20% sucrose cushion. Infectious end-point viral titers were determined on HeLa-TZMbl cells as previously described (Le Tortorec and Neil, 2009).

Production of HIV-1 env pseudotyped viral vectors

Generation of recombinant env-pseudotyped HIV-1 vectors, encoding GFP, was performed by co-transfection of 293T cells with pCRV1 HIV-1 gag-pol, pCSGW (packaging vector containing the eGFP reporter gene) and the pCVRV1 or pSVIII plasmid, as indicated, encoding the Env protein of interest. The ratio of the HIV-1 gag-pol plasmid DNA to pCSGW to pCRV1/pSVIII env plasmid DNA was optimised at a 2:3:1 ratio. Viral supernatants were harvested 48 hrs post transfection, filtered and titred on the appropriate U87-MG cell line by flow cytometry.

For neutralisation assays, pseudotyped virus was produced in 293T cells by co-transfecting the plasmids encoding Env with pSG3ΔEnv (an Env-deficient genomic backbone plasmid) in a 1:2 ratio using polyethylenimine (PEI) at 1 mg/ml. 48 hrs post transfection, viral supernatants were harvested and filtered. Viral titre was determined by titration of pseudotyped virus stocks onto HeLa-TZMbl cells and culturing for 48 hrs prior to calculating the $TC1D_{50}$ values according to the Reed and Meunch equation.

Generation of U87-MG IFITM stable cell lines

U87-MG CD4-CXCR4 and U87-MG CD4-CCR5 cell lines stably expressing the IFITMS 1, 2, 3, IFITM2-Y19F or IFITM3-Y20F proteins, with and without HA-tags, were generated by retroviral transduction. Cells were selected with 100 μg/ml hygromycin.

Expression of IFITM proteins was assessed by western blot. Cell lysates were subjected to SDS-PAGE, and Western blot assays were performed using rabbit-anti HSP90 (Santa Cruz), mouse anti-IFITM1 and rabbit anti-IFITM2 or 3 (ProteinTech) antibodies. When indicated, IFN-α (universal type 1 IFN, PBL InterferonSource) stimulation was performed using 1000 U ml^{-1} for 24 h before immunoblotting or virus infection.

Flow cytometry

Flow cytometry analyses were performed using a BD FACSCanto II flow-cytometer (Becton Dickinson) and FlowJo software. Env-pseudotyped HIV-1 viral vector infected cells were analysed for expression of GFP. CD4, CXCR4 and CCR5 surface expression in the presence of IFITM 1, 2 or 3 were determined by staining with APC conjugated mouse anti-human CD4 (BD Pharmingen), PE conjugated mouse CXCR4 (CD184) (BD Pharmingen) and PE conjugated mouse CCR5 (CD195) (BD Pharmingen), respectively.

Viral replication assays

U87 CD4$^+$ CXCR4$^+$ or CD4$^+$ CCR5$^+$ cells stably expressing IFITMs 1, 2 or 3 or mutants thereof were infected with the indicated HIV-1 molecular clone at a multiplicity of infection (MOI) of 0.05. Media was replaced 8 hrs post infection and culture supernatants were harvested and stored every 24 hours post infection for a total of 120 hrs. Infectious viral release at each time point was determined by infecting HeLa-TZMbl indicator cells and 48 hrs post infection assaying for virus release by measuring chemiluminescent β-galactosidase activity using the Tropix Galacto-Star system (Applied Biosystems) according to the manufacturer's instructions. For one-round virus release assays, cells were infected with the indicated HIV-1 molecular clone at an MOI of 0.5. Viral production was measured for supernatants harvested at 48 hrs post infection on HeLa-TZMbl indicator cells, as above.

For analysis of envelope content of physical virus particles, supernatants were filtered through a 0.22 μm filter (Merck Millipore) and pelleted through a 20% sucrose/PBS cushion at 28 000 rpm for 90 min at 4 °C. Virion and cell lysates were subjected to SDS-PAGE and Western blotted for HIV-1 p24CA using monoclonal antibody 183-H12-5C; kindly provided by B. Chesebro through the NIH AIDS Reagent program, and for HIV-1 gp160 and gp120 using antiserum to HIV-1 gp120 (ARP421) obtained from the Centre of AIDS reagents.

siRNA mediated AP2 knockdown

siRNA mediated knock down of AP2 in U87-MG CD4$^+$ CXCR4$^+$ or CD4$^+$ CCR5$^+$ control cells or cells expressing IFITMS 1, 2, 3, IFITM2-Y19F or IFITM3-Y20F was performed as previously described (Kueck and Neil, 2012). SMARTpool siRNA was purchased from Dharmacon (#L-008170-00-0005) and represents a pool of four different siRNAs
(1, GAACCGAAGCUGAACUACA; 2, AGUUUGAGCUUAUGAGGUA;
3, GCGAGAGGGUAUCAAGUAU; 4, GUUAAGCGGUCCAACAUUU) (Thermo Scientific). Non-targeting siRNA was used as a control (Thermo Scientific). Viral supernatants were harvested at 48 hrs and infectivity was determined by infecting HeLa-TZMbl indicator cells. Cell lysates were subjected to SDS-PAGE, and Western blot assays were performed using a rabbit-anti HSP90 (Santa Cruz), and mouse monoclonal anti-AP50 antibody (BD Biosciences).

Endocytosis Inhibitor studies

Dynamin inhibitor, Dynasore monohydrate (Sigma), and Clathrin inhibitor Pitstop2 (Abcam) were dissolved in dimethyl sulphosxide (DMSO). Cells were treated with 80 μM Dynasore, 30 μM Pitstop2 or DMSO alone for 30 minutes at 37 °C prior to infection with env-pseudotyped HIV-1 viral vectors for 48 hours. Following incubation, cells were analysed for viral vector entry by flow cytometry. AlexaFluor 488 conjugated Transferrin (Thermo Fisher Scientific) was used as a control for the inhibition studies. 50 μg/ml transferrin was added to Dynasore or Pitstop2 treated cells on coverslips for 10 minutes at room temperature. Cells were washed 3 x in the presence or absence of the endocytosis inhibitors before incubation at 37 °C for 30 minutes. Following incubations, cells were fixed in 4% paraformaldehyde/PBS and washed with 10 mM glycine/ PBS.

Cells were mounted on glass slides using ProLong AntiFade- 4',6-diamidino-2-phenylindole (DAPI) mounting solution (Molecular Probes, Invitrogen) and images were captured with a Nikon ESCLIPSE Ti inverted microscope. Z stacks were

taken of all cells, images deconvoluted using AutoQuant X3 and analyzed using the ImageJ software. Representative images are shown.

Lentiviral mediated shRNA knockdown of IFITM expression

Silencing of IFITM expression was mediated by lentiviral shRNA knockdown in primary human CD4+ T cells. IFITM1, IFITM2 and IFITM3 specific hairpins were cloned into pHRSIREN-PGK-GFP (Matheson et al., 2014) encoding a U6-shRNA cassette and GFP for flow cytometric analysis. The targeting (sense) sequences used were the following:

shIFITM1: GGTCCACCGTGATCAACAT

shIFITM2: GCCACGTACTCTATCTTCCAT

shIFITM3: GCCCACGTACTCCAACTTCCT

Control: shLacZ control: GTTATAGGCTCGCAAAAGG

VSV-g pseudotyped pHRSIREN lentiviral vectors were produced by co-transfection of 293T cells with pCRV1 HIV-1 *gag-pol*, pHRSIREN-PGK-GFP and pMD.G VSV-g (at a ratio of 2:3:1), and primary human CD4+ T cells were transduced with at an input equivalent to a MOI of 5, when titered on HeLa-TZMbl cells, and cultured for 48-72h before assay use. Transduction efficiency was assessed by flow cytometry (typically between 80 and 98% efficiency of transduction and silencing efficiency was assessed by Western blot. Cell lysates were subjected to SDS-PAGE, and Western blot assays were performed using rabbit-anti HSP90 (Santa Cruz), mouse anti-IFITM1 and rabbit anti-IFITM2 or 3 (ProteinTech) antibodies. When indicated, IFN-α (universal type 1 IFN, PBL InterferonSource) stimulation was performed using 500 U ml^{-1} for 24 h before immunoblotting or virus infection.

Primary cell infections

2×10^5 activated $CD4^+$ T cells, transduced with the appropriate shRNA lentiviral vectors, were infected at an MOI of 0.1; 8 to 12 hours post infection, media was replaced. Supernatants were harvested every 72, 120 and 168 hrs post infection and virus particle production was assessed on HeLa-TZMbl cells as described previously.

CRISPR knockout of IFITM expression in U87-MG cells

CRISPR oligos were designed to target IFITM1 (TGATCACGGTGGACCTTGGA), IFITM2 (CATCTCGTAGTTGGGAGGC), IFITM3 (GCTCAAGGAGGAGCACGAGG) and Luciferase (CCTCTAGAGGATGGAACCGC). Oligos were cloned into an adapted version of the LentiCRISPR v.2 plasmid (Addgene), where the puromycin gene was replaced with the green florescent protein (GFP) gene, according to the manufacturer's instructions. Viral stocks were then produced in 293T cells. Briefly, cells were transfected with pCRVI HIV gag-pol, pMD2.G VSV-g and the LentiCRISPR v.2 GFP of interest at a ratio of 2.5: 5: 2.5 using 1 mg/ml polyethyleneimine. Media was changed 16 hrs post transfection and supernatant was harvested and filtered 48 hrs post transfection. U87-$CD4^+$ $CCR5^+$ cells transduced with the LentiCRISPR virus at 1,600 rpm for 1 hr. Cells were cultured for a further 7 days allowing for efficient knockout of the target protein. CRISPR knockout efficiency was then determined by western blot, where cell lysates were subjected to SDS-PAGE, and assays performed using rabbit-anti HSP90 (Santa Cruz), mouse anti-IFITM1 and rabbit anti-IFITM2 or 3 (ProteinTech) antibodies. When indicated, IFN-α (universal type 1 IFN, PBL InterferonSource) stimulation was performed using 1000 U ml^{-1} for 24 h before immunoblotting to determine level of IFITM knockout. The effect of the CRISPR knockouts on virus replication was assessed by a time course assay where supernatant was harvested every 72, 120 and 168 hrs.

Immunofluorescence

2 x 10^5 U87-MG $CD4^+$ $CXCR4^+$ or $CD4^+$ $CCR5^+$ cells stably expressing HA-tagged IFITM 1, 2 or 3 proteins or mutants thereof were grown on coverslips precoated with 0.1% (w/v) poly-L-lysine (Sigma). Cells were fixed in 4% paraformaldehyde/PBS, washed with 10 mM glycine/ PBS, and permeabilized in 1% bovine serum albumin/ 0.1% Triton-X-100/ PBS for 15 min. Cells were stained using anti-rabbit polyclonal HA antibody (Rockland) in combination with sheep anti-human CD46 or EEA1 (AbD Serotec), followed by the appropriate secondary antibodies conjugated to Alexa 488 or 594 fluorophores (Molecular Probes, Invitrogen). Cells were mounted on glass slides using ProLong AntiFade- 4',6-diamidino-2-phenylindole (DAPI) mounting solution (Molecular Probes, Invitrogen)

and images were captured with a Nikon ESCLIPSE Ti inverted microscope. Z stacks were taken of all cells, images deconvoluted using AutoQuant X3 and analyzed using the ImageJ software. Representative images are shown.

CD45-depletion and IFITM virion incorporation assays

U87-MG $CD4^+$ $CXCR4^+$ or $CD4^+$ $CCR5^+$ Control cells or cells expressing IFITMS 1, 2, 3, IFITM2-Y19F or IFITM3-Y20F were plated onto 10 cm dishes at a density of 2 x 10^6 were infected with HIV-1 molecular clone 89.6 at an MOI of 3. 72 hrs post-infection, supernatants were harvested, filtered and pelleted through a 20% sucrose/PBS cushion at 28 000 rpm for 90 min at 4 °C. Virus pellets were resuspended in PBS and prior to CD45 depletion, viral particles were normalised to HIV-1 p24-Gag ELISA (Perkin Elmer) according to manufacturer's instructions.

Matched p24 quantities were incubated with anti-CD45 antibody coupled magnetic beads (Miltenyi) for 2 hrs. Beads were recovered on a DynaMag-2 magnet (Invitrogen) following manufacturer's instructions. CD45-depleted and non-depleted fractions were then precipitated with a final concentration of 10% TCA and immunoblotted using mouse anti-IFITM1 and rabbit anti-IFITM2 or 3 (ProteinTech) antibodies, using monoclonal antibody 183-H12-5C for HIV-1 p24CA; kindly provided by B. Chesebro through the NIH AIDS Reagent program and mouse anti- human CD45 antibody (Biolegend) for determining the degree of CD45 depletion.

CD4 competition assays

U87-MG $CD4^+$ $CCR5^+$ were infected at an MOI of 0.5 with the indicated HIV-1 molecular clone/anti-human CD4 (SK3 clone-Biolegend) antibody mix. Anti-human CD4 antibody was used at concentrations of 100, 10, 0 ng/ml. Cells were carefully washed and media was replaced 6 hours post infection. 48 hrs post infection, supernatants were harvested and used to infect HeLa-TZMbl cells, assaying for any dose-dependent reduction in virus release by measuring chemiluminescent β-galactosidase activity using the Tropix Galacto-Star system (Applied Biosystems) according to the manufacturer's instructions.

Statistical analysis

Datasets were assessed for the significance of inhibitory differences between X4 and R5 using viruses (Figure 1C) using unpaired two-tailed T tests. Datasets in Figure 3B were assessed for statistical significance between T/F and match 6mo chronic clones using paired two-tailed Mann-Whitney tests. All statistical analyses were carried out using GraphPad Prism v5.0.b. Levels of significance were determined as follows: *** $p<0.001$, ** $p<0.01$, *$p<0.05$, ns $p>0.05$.

Supplemental References

Goujon, C., Schaller, T., Galao, R.P., Amie, S.M., Kim, B., Olivieri, K., Neil, S.J., and Malim, M.H. (2013). Evidence for IFNalpha-induced, SAMHD1-independent inhibitors of early HIV-1 infection. Retrovirology *10*, 23.

Kueck, T., and Neil, S.J. (2012). A cytoplasmic tail determinant in HIV-1 Vpu mediates targeting of tetherin for endosomal degradation and counteracts interferon-induced restriction. PLoS Pathog *8*, e1002609.

Le Tortorec, A., and Neil, S.J. (2009). Antagonism to and intracellular sequestration of human tetherin by the human immunodeficiency virus type 2 envelope glycoprotein. J Virol *83*, 11966-11978.
